# Unsupervised clustering for sepsis identification in large-scale patient data: a model development and validation study

**DOI:** 10.1186/s40635-025-00744-w

**Published:** 2025-03-20

**Authors:** Na Li, Kiarash Riazi, Jie Pan, Kednapa Thavorn, Jennifer Ziegler, Bram Rochwerg, Hude Quan, Hallie C. Prescott, Peter M. Dodek, Bing Li, Alain Gervais, Allan Garland

**Affiliations:** 1 Department of Community Health Sciences, Cumming School of Medicine, University of Calgary, CWPH 5E34, 3280 Hospital Dr. NW, Calgary, AB T2N 4Z6 Canada; 2 Centre for Health Informatics, University of Calgary, Alberta, Canada; 3 Department of Computing and Software, McMaster University, Hamilton, ON Canada; 4 Department of Medicine, McMaster University, Hamilton, ON Canada; 5 Ottawa Hospital Research Institute, Ottawa, ON Canada; 6 School of Epidemiology and Public Health, University of Ottawa, Ottawa, ON Canada; 7 Department of Medicine, University of Manitoba, Winnipeg, MB Canada; 8 Department of Community Health Sciences, University of Manitoba, Winnipeg, MB Canada; 9 Department of Health Research Methods, Evidence and Impact, McMaster University, Hamilton, ON Canada; 10 Department of Medicine, University of Michigan, Ann Arbor, MI USA; 11 Division of Critical Care Medicine and Center for Advancing Health Outcomes, St. Paul’S Hospital and University of British Columbia, Vancouver, BC Canada; 12 Alberta Health Services Analytics and Strategy for Patient-Oriented Research (SPOR), Calgary, AB Canada; 13 Centre de Recherche du CIUSSS de l’Estrie-CHUS, Université de Sherbrooke, Sherbrooke, Québec Canada

**Keywords:** Sepsis identification, Electronic health records, Unsupervised learning, Clustering, Adult sepsis event

## Abstract

**Background:**

Sepsis is a major global health problem. However, it lacks a true reference standard for case identification, complicating epidemiologic surveillance. Consensus definitions have changed multiple times, clinicians struggle to identify sepsis at the bedside, and differing identification algorithms generate wide variation in incidence rates. The two current identification approaches use codes from administrative data, or electronic health record (EHR)-based algorithms such as the Center for Disease Control Adult Sepsis Event (ASE); both have limitations. Here our primary purpose is to report initial steps in developing a novel approach to identifying sepsis using unsupervised clustering methods. Secondarily, we report preliminary analysis of resulting clusters, using identification by ASE criteria as a familiar comparator.

**Methods:**

This retrospective cohort study used hospital administrative and EHR data on adults admitted to intensive care units (ICUs) at five Canadian medical centres (2015–2017), with split development and validation cohorts. After preprocessing 592 variables (demographics, encounter characteristics, diagnoses, medications, laboratory tests, and clinical management) and applying data reduction, we presented 55 principal components to eight different clustering algorithms. An automated elbow method determined the optimal number of clusters, and the optimal algorithm was selected based on clustering metrics for consistency, separation, distribution and stability. Cluster membership in the validation cohort was assigned using an XGBoost model trained to predict cluster membership in the development cohort. For cluster analysis, we prospectively subdivided clusters by their fractions meeting ASE criteria (≥ 50% ASE-majority clusters vs. ASE-minority clusters), and compared their characteristics.

**Results:**

There were 3660 patients in the development cohort and 3012 in the validation cohort, of which 21.5% (development) and 19.1% (validation) were ASE (+). The Robust and Sparse K-means Clustering (RSKC) method performed best. In the development cohort, it identified 48 clusters of hospitalizations; 11 ASE-majority clusters contained 22.4% of all patients but 77.8% of all ASE (+) patients. 34.9% of the 209 ASE (−) patients in the ASE-majority clusters met more liberal ASE criteria for sepsis. Findings were consistent in the validation cohort.

**Conclusions:**

Unsupervised clustering applied to diverse, large-scale medical data offers a promising approach to the identification of sepsis phenotypes for epidemiological surveillance.

**Supplementary Information:**

The online version contains supplementary material available at 10.1186/s40635-025-00744-w.

## Take-home message

This study is the first to develop and explore unsupervised clustering methods for sepsis identification without a priori labeling, using large-scale medical record data from adults admitted to intensive care units at five Canadian medical centres, 2015–2017. The best-performing algorithm emerged from evaluation of eight clustering methods to identify sepsis, representing a promising direction for real-world sepsis identification.

## Introduction

The World Health Organization (WHO) recognizes surveillance as essential to improve disease prevention and outcomes [1, 2]. It recognizes sepsis as a global health priority, and in 2017 called on countries to implement epidemiologic surveillance to monitor sepsis incidence and outcomes.

Estimated to affect 49 million people annually, and cause 20% of global deaths [3], there are important controversies about sepsis definition and identification. In the most recent consensus-based conceptualization, sepsis is “life-threatening organ dysfunction caused by a dysregulated host response to infection” [4]. However, sepsis is a syndrome lacking a true reference standard for case identification, and consensus definitions have changed multiple times over the past 25 years [4, 5]. Clinicians struggle to identify sepsis at the bedside [6, 7], and differing identification algorithms generate wide variation in incidence rates [8].

The two identification approaches currently used for sepsis epidemiological studies are code-based algorithms using hospital administrative data [9, 10] and electronic health record (EHR)-based algorithms such as the Adult Sepsis Event (ASE) criteria [11, 12]. Both begin by defining sepsis as concomitant infection and acute organ dysfunction, and seek to identify hospitalized patients having both. Implicitly for code-based approaches, and explicitly for the EHR-based approaches, there are minimum thresholds for the degree of organ dysfunction, e.g., the ASE criteria defines acute hepatic dysfunction as total bilirubin ≥ 34.2 μmol/L and increased 100% from baseline [12]. The ASE criteria also include a minimum duration for antimicrobial therapy, and a maximum interval between appearance of infection and acute organ dysfunction. However, such thresholds overlook that fact that the processes underlying infection-induced acute organ dysfunction constitute a continuum from very mild to very severe. By missing the full clinical range, threshold-based identification algorithms almost certainly underestimate the population burden of sepsis [13, 14].

Here, we describe the initial developmental steps of a novel approach to identification of sepsis for epidemiologic purposes, applying unsupervised machine learning clustering algorithms to population-based health data. These algorithms can detect patterns in large data sets, making them well-suited for identifying the full spectrum of sepsis subphenotypes, seeking to move toward more holistic identification without any a priori definition of a complex condition lacking a true reference standard [15, 16]. Real-time identification of patients with sepsis or at high risk of sepsis, with the goal of reducing sepsis mortality through early recognition [17], is distinct from epidemiologic surveillance, and is not part of our work.

For this first report of our novel approach, our primary aim was to describe and externally validate our unsupervised learning methods, emphasizing technical considerations of cluster consistency, separation, distribution, stability and reproducibility. Our secondary aim was to perform analyses on the initial clustering results to begin assessing the potential of unsupervised clustering for identifying sepsis. For this secondary purpose, using ASE criteria as a comparator—not a reference standard—we subdivided and compared clusters by their fraction meeting the ASE criteria (ASE-majority vs. ASE-minority clusters). We hypothesized that within ASE-majority clusters, many ASE (−) members would be similar to the ASE (+) members, being abnormal but not meeting the set ASE thresholds.

## Methods

We conducted a retrospective cohort study including the first hospitalization of adult patients that required ICU admission in the Calgary Health Region of Canada between April 2015 and March 2017. This region includes two teaching hospitals, and three other large urban hospitals.

We linked inpatient and outpatient administrative and electronic data from multiple sources: the Discharge Abstract Database (DAD), National Ambulatory Care Reporting System (NACRS), Sunrise Clinical Manager (SCM) EHR, and eCritical Alberta (eTable A1). The study was approved by the Conjoint Health Research Ethics Board of the University of Calgary (REB21-0921).

**Fig. 1:**
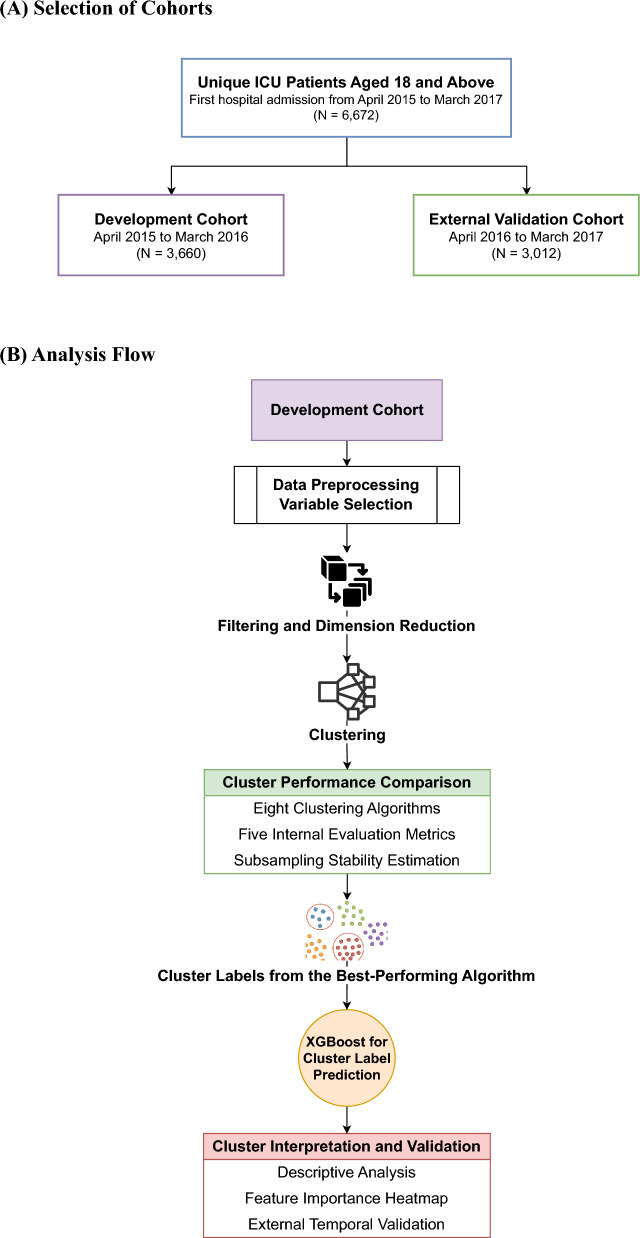
Cohorts and analysis flow

We serially performed data preparation, variable selection, model building, interpretation, and external validation (Fig. 1). Modelling comprised two parts, yielding a prediction tool without using any a priori labelling of patients. First, unsupervised clustering identified subgroups sharing similarities in underlying relationships among variables. Being independent of pre-assigned definitions of clinical entities such as sepsis (‘labels’) allowed the data to “speak for itself” in subdividing patients into subgroups. Then, using the same variables, we applied supervised, eXtreme Gradient Boosting (XGBoost) [18] to create a multi-class model predicting membership in the clusters derived from unsupervised clustering. This allowed for the use of diagnostic tools available after XGBoost to better understand the nature of, and differences between, clusters, and enabled external validation. Analyses were performed using R version 4.3.1 and SAS® Enterprise Guide®7.1.

### Data pre-processing and variable reduction

We pre-processed 592 patient variables, encompassing encounter characteristics, demographics, diagnoses, medications, laboratory tests, and clinical management (eTable C2). As our goal was not real-time identification, we included variables across the timespan of the hospital encounter. We assessed for the 31 chronic comorbid conditions described by Elixhauser et al. [19] identified from hospital administrative data, using established ICD-10-CA coding [20]. We converted categorical variables into binary indicators, and standardized continuous variables to prevent domination by extreme value ranges [21]. We imputed missing values as the mean of available, non-missing values (eTables C1, C2) [22, 23]. Vital signs and laboratory tests measured multiple times during hospitalization were summarized by calculating the minimum, maximum, and standard deviation over time (see eTable C2), reflecting extremes and fluctuations in each patient’s physiological state. We removed pre-processed variables if: they had < 1% prevalence; there was evidence of high collinearity with others; they were determined as clinically irrelevant or redundant based on a quality assurance review by clinical experts (e.g., blood–urea–nitrogen, often recorded under different names in EHRs, such as “BUN” and “UREA”, was considered duplicative; the variables were reconciled and the duplicate was excluded); they were timing variables as we included the relevant derived time intervals (e.g., admission/discharge date removed, length of stay retained); and if they were direct outcome variables (ASE sepsis indicator, death). The expert review served solely as quality assurance to verify clinical relevance and identify data quality issues, without influencing variable selection or the data-driven modeling process.

After pre-processing we performed variable reduction via the Kaiser–Meyer–Olkin test (KMO) [24], followed by Principal Component Analysis (PCA) [25]. KMO assesses whether PCA is suitable for a data set, with low values indicating ineffective PCA, we removed variables with KMO < 0.6 [26]. PCA replaces the original variables with a smaller number of linear combinations (principal components) encompassing most of the original information content. We retained principal components to include 95% of total variance [26]. We chose these thresholds based on established guidelines in PCA and exploratory factor analysis [26, 27]. Although KMO of 0.6 is considered on the lower end [28], it allows us to preserve the richness of the data set and explore the complex variable relationships among ICU patients while maintaining established statistical standards.

### Clustering analysis for patient segmentation

We assessed eight unsupervised clustering algorithms (eTables A2, A3). These included distance-based approaches (K-means [29] and Mini-Batch K-means [30]) which excel at identifying clusters with a minimal within-cluster variance), Weighted K-means [31] for its feature prioritization capabilities, Robust and Sparse K-means Clustering (RSKC) [32] for its resilience to outliers and high-dimensional data, probability-based Gaussian Mixture Modeling (GMM) [33], adept at handling clusters of varying shapes and sizes, Clustering LARge Applications (CLARA) [34] for its efficiency with large data sets, Self-Organizing Maps (SOM) [35] for managing complex data structures, and regularized Deep Clustering with Auto-Encoder (DCAE) [36] for its proficiency in feature extraction of high-dimensional data.

We used an automated elbow method to determine the optimal number of clusters for each method [37]. This runs an algorithm across a range of cluster counts (here 2–120), seeking the maximum within-cluster similarity, measured as the sum of squared intra-cluster distances. The optimal number is where the rate of decrease in this measure sharply falls, resembling an elbow on a scree plot [38].

### Cluster performance evaluation

We used the first year of data for the model development and applied five internal clustering metrics to compare cluster consistency, separation, and distribution from the algorithms. Silhouette coefficient [39] measures cluster consistency, evaluating how similar an observation is to its cluster vs. the others; higher values indicate better matching to its own cluster. Davies–Bouldin (DB) index [40] measures cluster separation by averaging the maximum similarity across all clusters; lower values indicate more distinct clusters. Calinski–Harabasz (CH) index [41] is the ratio of cumulative between-cluster to within-cluster dispersion; higher values indicate better-defined clusters. Shannon Diversity index [42] measures the data set diversity by evaluating how evenly data points are distributed among clusters; higher values signify more balanced distribution among clusters, with no single cluster disproportionately dominating. Gini index [43] measures inequality in cluster sizes; ranging from 0 (all clusters equal in size) to 1 (one cluster includes all subjects). We ranked the eight algorithms using each of the five metrics, with lower ranks indicating better performance; overall ranking was the sum of the individual ranks.

Moving forward only with the two algorithms with the best (lowest) overall ranking, we selected the globally best-performing algorithm as the one with the best clustering stability, which helps ensure result reliability and robustness [44]. We evaluated clustering stability with the subsampling stability estimation method [45, 46]; generating 200 subsamples each containing a random 80% of the original data. Then, each clustering algorithm was applied 100 times on each subsample to test the impact of initialization and parameter settings. We quantified stability as the Adjusted Rand Index (ARI) [47], and Normalized Mutual Information (NMI) [48], which measures agreement between different clustering runs on the same subsample. Means and standard deviations (SD) for these metrics were calculated across subsamples, with clustering stability assessed by the coefficient of variation (CV), with lower CV indicating better stability. A diagram of the cluster performance evaluation strategy is shown in eFigure A1.

### Assessing differential characteristics of clusters

For each cluster output produced by the XGBoost model, we used the Shapley Additive exPlanation values (SHAP) [49] seeking to identify the most impactful features and the key characteristics that distinguish it from the others. We also compared categories of primary diagnosis by cluster. For this purpose we used ICD-10-CA-coded diagnoses in hospital administrative data. Specifically we used the Most Responsible Hospital Diagnosis (MRHD) from the DAD, defined as the diagnosis responsible for the greatest portion of the length of stay [50], which we categorized into the 21 ICD-10 chapters [51, 52]. We did not use admission diagnosis, because: (a) it is not a required field in Canadian hospital abstracts, and (b) that designation is known to have poor abstraction–reabstraction consistentcy [53].

### Cluster interpretation

We calculated the percentage of ASE (+) cases in each cluster, and subdivided them into the ASE-majority clusters (≥ 50% ASE) and the ASE-minority clusters (< 50% ASE). Within the ASE-majority clusters, we compared ASE (−) to ASE (+) patients using standardized differences interpreted as [54]: negligible (0 ≤ *d* ≤ 0.2), small (0.2 < *d* ≤ 0.4), medium (0.4 < *d* ≤ 0.8), and large (*d* > 0.8). We compared distributions of key laboratory results using density plots and boxplots. To help visualize cluster separation, we plotted individuals on the first two principal components.

We compared our new method of identifying sepsis patients with the ASE criteria. We assessed whether ASE (−) members of the ASE-majority clusters met more liberal thresholds for one or more of: organ dysfunction, interval between infection and organ dysfunction, and antimicrobial duration (eTable A5). This sensitivity analysis was performed to investigate whether the observed heterogeneity in ASE status within clusters was due to the strict nature of the original ASE thresholds rather than differences in the underlying clinical characteristics captured by our clustering approach. We also compared the ASE (+) members of the ASE-majority clusters and the ASE-minority clusters.

### Clinical expert evaluation of clusters

Two clinical ICU experts (AG, JZ), working independently and blinded to outcome variables (e.g., ASE label, sepsis/septic shock ICD codes, and mortality), assessed the 48 clusters, by the best-performing algorithm from the development cohort. They assigned them to clinical categories and identified distinguishing clinical characteristics within categories, using summary statistics of patient characteristics including 110 comorbidity conditions and 68 demographic, laboratory, and medication variables. They then met virtually to reach consensus on the final labels and characteristics.

### External validation

We conducted external validation using the second year of data. We used the XGBoost model, trained to predict cluster membership in the development cohort, to assign all validation cohort subjects to one of the 48 clusters. The model was developed using the same 72 variables that passed the KMO filtering criterion, with the goals of predicting cluster membership likelihood for each patient and creating a reusable model for new data. We applied Bayesian Optimization for automatic XGBoost hyperparameter tuning, such as the learning rate, tree depth, subsample ratio of the training instances, and number of cross validation folds, to maximize performance while avoiding overfitting [55]. The trained model assigned cluster labels to the validation cohort based on predicted probabilities, with each patient assigned to the cluster corresponding to the highest probability. Proportions of ASE (+) cases by cluster were compared between the two cohorts [56]. We assessed for differences between the development and external validation cohorts as standardized differences [54].

## Results

### Primary aim: clustering methods

The development and validation cohorts comprised, respectively, 3660 and 3012 unique ICU patients, with similar characteristics (Table 1, eTable B1). Ten variables had > 0.6% missing data related to laboratory tests, with highest missingness (40.3%) for serum lactate (eTable C2).

**Table 1:** Patient characteristics of patient cohorts. Values are n (%) unless otherwise indicated

Characteristic N (%) unless otherwise indicated	Development cohort April 2015–March 2016 N = 3660	External validation cohort April 2016–March 2017 N = 3012	Standardized difference between the two cohorts
Age, median (interquartile range [IQR])	62 (52–73)	62 (52–72)	0.03
Female	1315 (36)	956 (32)	0.09
Clinical characteristics during hospitalization			
Admitted via emergency department	2430 (66)	1,944 (65)	0.04
ICU length of stay, median (IQR)	3 (2–5)	3 (2–5)	0.04
Hospital length of stay, days, median (IQR)	8 (5–17)	7 (4–15)	0.09
Received invasive mechanical ventilation	1427 (39)	1198 (40)	0.02
Blood culture collected	1348 (37)	960 (32)	− 0.10
Received antimicrobial medications	2163 (59)	1682 (56)	− 0.07
For 2 or more consecutive days	1979 (54)	1528 (51)	− 0.07
Received intravenous vasopressor therapy	684 (19)	481 (16)	− 0.07
Highest serum lactate (in mmol/L), median (IQR)	2.6 (1.6–4.8)	2.6 (1.7–4.7)	0.02
Highest serum creatinine (in μmol/L), median (IQR)	95 (77–128)	97 (80–123)	0.03
Highest serum total bilirubin (in μmol/L), median (IQR)	11 (7–19)	11 (7–18)	0.05
Lowest serum platelet count (in 10^9^/L), median (IQR)	166 (120–212)	168 (121–212)	− 0.02
In-hospital mortality	419 (11)	320 (11)	− 0.03
Sepsis cases by comparator criteria			
Adult Sepsis Event (ASE) definition	787 (21.5)	576 (19.1)	− 0.06

Thresholds for the absolute values of standardized differences: 0 ≤ *d* ≤ 0.2 negligible effect size; 0.2 < *d* ≤ 0.4 small effect size (*); 0.4 < *d* ≤ 0.8 medium effect size (**); *d* > 0.8 large effect size (***). See eTable B1 for additional characteristics

Of 592 original variables, 231 met pre-processing criteria, of which 72 passed the KMO filtering criterion, leading to 55 principal components that included 95% of total original variance that were used in all subsequent modelling, but cannot be simply interpreted in terms of the original variables. Organized in descending order of original information content, the first two components explained, respectively, 22.3% and 7.3% of total variance.

In the development cohort, the optimal number of clusters was 48 for all eight clustering algorithms (eTable B2, eFigure B1). Over the five internal evaluation metrics, K-means and RSKC were the top-ranked algorithms (Table 2). As RSKC demonstrated much greater subsampling stability compared to K-means (eFigure B2), it was used exclusively for all subsequent analyses.

**Table 2:** Internal cluster validation statistics in the development cohort of different clustering methods

Method	Silhouette coefficient (rank^1^)	Davies–Bouldin index (rank^2^)	Calinski–Harabasz index (rank^3^)	Shannon diversity index (rank^4^)	Gini index (rank^5^)	Final rank^6^
**K-Means**	− **0.133 (2)**	**8.330 (4)**	**73.175 (2)**	**3.568 (4)**	**0.481 (1)**	**1**
**RSKC**	− **0.153 (4)**	**7.874 (3)**	**56.49 (4)**	**3.701 (2)**	**0.487 (2)**	**2**
CLARA	− 0.123 (1)	6.319 (2)	55.568 (5)	2.900 (7)	0.523 (6)	3
SOM	− 0.174 (5)	10.963 (7)	110.067 (1)	3.353 (5)	0.492 (4)	4
Regularized DCAE	− 0.142 (3)	9.538 (6)	58.503 (3)	3.712 (1)	0.529 (8)	5
GMM	− 0.189 (6)	8.638 (5)	54.859 (6)	3.646 (3)	0.510 (5)	6
Mini Batch K-Means	− 0.230 (7)	4.214 (1)	53.357 (7)	2.595 (8)	0.528 (7)	6
Weighted K-Means	− 0.614 (8)	17.693 (8)	1.473 (8)	3.229 (6)	0.491 (3)	8

The bolded values indicate the top two algorithms with the best overall rankings across five internal evaluation metrics, which were subsequently selected for clustering stability assessment

*GMM* Gaussian Mixture Model, *CLARA* Clustering large Applications, *RSKC* Robust and Sparse K-means Clustering, *SOM* Self-Organized Map, *DCAE* Deep Clustering with Auto-Encoder

^1^Silhouette coefficient [39] measures cluster consistency, higher values being better

^2^Davies–Bouldin index [40] measures cluster separation, lower values are better

^3^Calinski–Harabasz index [41] assesses cluster dispersion, higher values being better

^4^Shannon Diversity index [42] measures data set diversity, higher values are better

^5^Gini index [43] measures inequality in cluster sizes, with lower values being better

^6^Final ranking was determined by summing all five ranking orders and was ordered from smallest to largest to identify the top-performing algorithms

### Secondary aim: cluster analysis

In the development cohort, 787 (21.5%) patients were ASE (+) (Table 1). Eleven clusters containing 821 patients comprised the ASE-majority clusters; of which 612 (74.5%) were ASE (+), representing 77.8% of all ASE (+) subjects in the cohort (eTables B4, B6, B7). In contrast, of 2839 patients in the 37 ASE-minority clusters, only 175 (6.2%) were ASE (+), representing 22.2% of all ASE (+) subjects.

Consistent with the clinical heterogeneity of sepsis, the XGBoost feature importance heatmap and top features by SHAP found no evidence of consistent patterns or dominant features in the ASE-majority clusters (eFigure B3, eTable B4). Regarding the most common category of MRHD, there was more variation within the ASE-majority vs. ASE-minority clusters, as manifested in two ways. First (1st and 4th data columns of eTable B5), just three diagnostic categories contained the most common MRHD for all 37 ASE-minority clusters, with diseases of the circulatory system being that category for 81% of 37 clusters. This is consistent with the known dominance of cardiovascular disorders as reasons for admission to hospitals and ICUs [57, 58]. On the other hand, among the 11 ASE-majority clusters, five different categories were represented (respiratory; infectious; circulatory; digestive; external causes including injury and poisonings), with none representing more than 37% of those 11 clusters. Second (2nd and 5th data columns of eTable B5), within the most common categories of MRHD, patients were less concentrated for ASE-majority vs. ASE-minority clusters. Third (3rd and 6th data columns of eTable B5), ASE cases were more concentrated in ASE-majority clusters than in ASE-minority clusters within the most common categories of MRHD.

Compared to the 37 other clusters, patients in ASE-majority clusters had: (a) slightly lower age at admission; (b) higher rates of culture collection, (c) more antimicrobial, mechanical ventilation, and vasopressors use, (d) longer lengths of stay, and (e) laboratory values with more extreme derangements and greater variability over the course of hospitalization (eTable B6). On the first two principal components, there was good separation between the ASE-majority and ASE-minority clusters (eFigure B4A).

In the ASE-majority clusters, 209 patients (25.5%) were ASE (−). For most variables, standardized differences between ASE (−) and ASE (+) patients were negligible (Table 3, eTable B3). Non-negligibly different variables were: rates of blood culture and use of antimicrobials, values for creatinine, bilirubin, eGFR, oxygenation, number of organ failures, and ICU length of stay (Table 3). We found evidence that many ASE (−) patients in the ASE-majority clusters would meet liberalized ASE criteria. First, on the first two principal components, there was great overlap between ASE (+) and ASE (−) patients, indicating their similarity (red and green dots, eFigure B4B). Second, many ASE (−) patients had acute organ dysfunction but below ASE thresholds, e.g., 12 patients had creatinine rises of 150–200%. Beyond specific thresholds, differences in continuous parameters of organ function between ASE (+) and ASE (−) patients were relatively small (eFigure B5). Third, changing the maximum interval between blood culture and the first organ dysfunction from 2–3 or 4 days reclassified 30–59 patients to ASE (+). Fourth, liberalizing the duration of antimicrobials from 4 to 3 days captured an additional 13 patients. Including all liberalized ASE criteria (Table 4, eTable A5) re-categorized 34.9% (73/209) of the ASE (−) patients in ASE-majority clusters as having sepsis.

**Table 3:** Development cohort patient characteristics between ASE (+) (Adult Sepsis Event) and ASE (−) cases among the 11 ASE-majority clusters. Values are n (%) unless otherwise indicated

Characteristics	All *N* = 821	ASE (−) *N* = 209	ASE (+) *N* = 612	Standardized difference
Age, median (interquartile range [IQR])	61 (51–72)	61 (52–75)	61 (50–71)	0.09
Female	351 (42.8)	83 (39.7)	268 (43.8)	0.08
Clinical characteristics during hospitalization				
Admitted via emergency department	651 (79.3)	147 (70.3)	504 (82.4)	− 0.28*
ICU length of stay, median (IQR)	7 (4–12)	6 (3–11)	7 (4–12)	− 0.22*
Blood culture collected	779 (94.9)	167 (79.9)	612 (100.0)	0.93**
Received antimicrobial medications	814 (99.1)	202 (96.7)	612 (100.0)	0.37*
for 2 or more consecutive days	810 (98.7)	198 (94.7)	612 (100.0)	0.46**
Died during hospitalization	255 (31.1)	54 (25.8)	201 (32.8)	0.15
Acute organ dysfunction per ASE criteria: (Any one of the following ± 2 days of blood culture)				
Number of acute organ dysfunctions, median (IQR)	3 (2–4)	2 (1–3)	3 (2–4)	− 0.69**
Respiratory (invasive mechanical ventilation)	432 (52.6)	87 (41.6)	345 (56.4)	0.30*
Renal	452 (55.1)	93 (44.5)	359 (58.7)	0.28*
Liver	135 (16.4)	21 (10.0)	114 (18.6)	0.25*
Hematologic	291 (35.4)	61 (29.2)	230 (37.6)	0.18
Cardiac dysfunction (received intravenous vasopressor)	493 (60.0)	85 (40.7)	408 (66.7)	0.53**
Elevated lactate	676 (82.3)	148 (70.8)	528 (86.3)	0.38*
Lab test results, median (IQR)				
Highest serum lactate (in mmol/L)	4.0 (2.3–7.8)	3.7 (1.8–7.4)	4.2 (2.5–8.0)	− 0.13
Highest serum creatinine (in μmol/L)	146 (92–253)	125 (83–225)	156 (97–269)	− 0.03
Highest/lowest serum creatinine ratio	2.1 (1.7–3.3)	1.9 (1.7–2.6)	2.2 (1.7–3.6)	− 0.25*
Lowest estimated glomerular filtration rate (eGFR)	40 (19–71)	51 (23–79)	37 (18–69)	0.21*
Lowest/highest eGFR ratio	0.5 (0.3–0.7)	0.6 (0.4–0.7)	0.5 (0.3–0.7)	0.31*
Highest serum total bilirubin (in μmol/L)	15 (9–31)	12 (7–24)	16 (10–39)	− 0.20
Highest/lowest serum total bilirubin ratio	2.0 (1.3–3.5)	1.6 (1.0–2.9)	2.2 (1.3–3.7)	− 0.24*
Lowest serum platelet count (in 10^9^/L)	119 (72–171)	133 (83–180)	117 (67–168)	0.16
Lowest/highest serum platelet count ratio	0.3 (0.2–0.5)	0.3 (0.2–0.5)	0.3 (0.2–0.5)	0.10
Lowest P/F ratio value	138 (82–202)	158 (102–228)	132 (78–193)	0.28*

Thresholds for the absolute values of standardized differences: 0 ≤ *d* ≤ 0.2 negligible effect size; 0.2 < *d* ≤ 0.4 small effect size (*); 0.4 < *d* ≤ 0.8 medium effect size (**); *d* > 0.8 large effect size (***). The highest (or lowest) lab test result was the maximum (or minimum) value during the entire hospitalization. The terms ‘highest/lowest ratio’ and ‘lowest/highest ratio’ refer to the ratios of the maximum to minimum lab results, and vice versa, during the entire hospitalization. See eTable B3 for additional characteristics

**Table 4:** Number (%) of the 209 Adult Sepsis Event-negative [ASE (−)] patients in the 11 ASE-majority clusters who would meet the altered definition of sepsis with less stringent thresholds

Altered ASE criteria	*N* (%)
Altered organ failure laboratory value thresholds	
a. Creatinine rise > 1.5-fold instead of > 2.0-fold or eGFR decline > 25% instead of > 50%	12 (5.7)
b. Maximal total bilirubin > 25.7 mM instead of > 34.2 with rise > 1.5-fold instead of > 2.0-fold	4 (1.9)
c. Lactate > 1.5 mM instead of > 2.0	11 (5.3)
d. Platelets < 150 × 10^9^/L instead of 100 with platelet decline > 25% instead of > 50%	8 (3.8)
e. All of a, b, c and d	26 (12.4)
Altered interval threshold between blood culture and first organ failure	
f. Maximum of 3 instead of 2 day interval	30 (14.4)
g. Maximum of 4 instead of 2 day interval	59 (28.2)
Altered minimum threshold for # antimicrobial days	
h. 3 days instead of 4	13 (6.2)
i. additional “or” criterion of up to and including day of leaving hospital alive to other than different acute hospital	9 (4.3)
Combined modified threshold criteria	
j. All of a, b, c, d, f, h, and i	73 (34.9)

Among all ASE (+) patients in the development cohort, there were dramatic differences between the 612 patients in the ASE-majority clusters and the 175 patients in the ASE-minority clusters (eTable B8). The latter had fewer acute organ dysfunctions and lower severity of illness.

The clinical evaluation process assigned the 48 clusters into eight categories (eTable B9): septic shock (7 clusters), sepsis without septic shock (3 clusters), respiratory (2 clusters), substance abuse/overdose (8 clusters), acute myocardial infarction (10 clusters), post-operative cardiovascular disorders (4 clusters), mixed cardiovascular disorders (10 clusters), and mixed disorders (4 clusters). Among the 10 clusters clinically categorized as sepsis or septic shock, 9 (90.0%) were ASE-majority clusters; the remaining cluster had the highest proportion of ASE (+) cases (46.4%) among the ASE-minority clusters, just below the 50% cutoff value. For the two ASE-majority clusters that were not categorized as sepsis or septic shock, one was assigned to substance abuse/overdose and the other to mixed disorders.

The external validation cohort included 576 (19.1%) ASE (+) patients (Table 1). Proportions of ASE (+) patients in the validation and development cohorts were tightly correlated (eFigure B7; Pearson correlation 0.973, *p* < 0.0001). Nine of the 11 ASE-majority clusters maintained ≥ 50% ASE (+) in the validation cohort; the two exceptions had 48.5% and 48.7% ASE-positivity. No clusters outside the ASE-majority clusters had > 50% ASE-positivity. Negligible differences were found on comparing key variables between patients in the ASE-majority clusters of the validation vs. development cohorts (eTable B10), except for use of invasive mechanical ventilation. Similar to the development cohort, only a small fraction (6.2%) of validation cohort patients outside the ASE-majority clusters were ASE (+).

## Discussion

In this multi-hospital cohort of over 6000 adult ICU patients, we report on the initial stages of developing and evaluating a novel approach to identifying sepsis hospitalizations, using unsupervised machine learning to automatically separate patients into a diverse collection of clusters without a priori labelling. After processing 592 variables, 55 principal components were presented to the clustering algorithms. Based on six evaluation metrics, the RSKC algorithm showed optimal clustering performance.

Prospectively subdividing these clusters by their fraction meeting ASE criteria (≥ 50% ASE-majority clusters vs. < 50% ASE-minority clusters; used as a familiar comparator, not a reference standard), there were noteworthy findings. The ASE-majority clusters contained 22% of all patients but 78% of all ASE (+) patients in the cohort; furthermore, many ASE (−) patients in the ASE-majority clusters were similar to its ASE (+) patients on most parameters, and met more liberal criteria for infection and acute organ dysfunction. In the threefold larger collection of ASE-minority clusters, only 6% of patients were ASE (+), representing 22% of all ASE (+) subjects in the cohort; with these ASE (+) patients having, on average, much lower severity of illness compared to ASE (+) patients in the ASE-majority clusters.

To assess clinical relevance, we performed an independent, blinded clinical evaluation of the data-driven clusters and assigned them to clinically meaningful categories. Although no firm clinical conclusions were drawn from this preliminary evaluation, it demonstrated that the data-driven clusters align with recognized clinical phenotypes for ICU patients. We acknowledge that these findings are exploratory and serve as a proof-of-concept. We judged, a *priori*, that further development of our approach will be required to separate out clinical subphenotypes that exist along the full spectrum of infection-induced acute organ dysfunction. However, as sepsis is a syndrome with large variability [59] and lacking a reference standard for operational identification of cases, these early findings are encouraging in that they strongly suggest: (a) automated clustering methods can effectively identify sepsis phenotypes, and (b) many sepsis cases, particularly milder ones, are missed by identification algorithms containing currently used thresholds for infection and organ dysfunction.

This approach seeks self-defined patient clusters interpretable as sepsis subphenotypes along an expanded clinical spectrum, instead of starting with a clinical definition and seeking patients who meet it. This spectrum may extend from the most severe requiring life support, to cases with minor organ dysfunction who receive antimicrobials and do well in regular hospital wards, or even as outpatients. Accordingly, with further refinement and wide application across healthcare settings, this approach may have the potential for a profoundly different approach to sepsis identification, and perhaps even to understanding sepsis. Here, in our initial assessment of this approach, we restricted consideration to an ICU cohort for reasons of efficiency and practicality, i.e., (i) the incidence of sepsis is expected to higher in an ICU than any other clinical setting, and (ii) the ASE criteria gave been mainly studied in ICU cohorts.

With one exception, prior studies seeking to systematically identify sepsis subphenotypes have pre-identified patients as having sepsis, for the purpose of stratifying them by characteristics, clinical trajectory, prognosis or response to therapy [60–65]. One group applied latent class analysis to three serum biomarkers (c-reactive protein, D-dimer, procalcitonin) among 765 patients in a single hospital in Columbia [66]. One of the two clusters identified, containing 24% of subjects, had 75% sepsis by consensus-based adjudication blinded to biomarkers.

This study has strengths. First, unsupervised learning can uncover insights and correlations missed by supervised methods due to their reliance on predefined labels. Second, these results used only input variables that are widely measured in hospitalized patients, i.e., excluding biomarkers not routinely measured. Third, we included a wide range of variables, including parameters rarely included as independent variables, such as standard deviations of laboratory and vital signs data. This allows us to detect evolving or fluctuating organ dysfunction that may not meet the fixed thresholds of the ASE criteria, offering a more dynamic view of the patient’s condition by capturing variability over time. Fourth, we evaluated eight unsupervised clustering methods, using a comprehensive set of metrics and validation analyses to conclude that the RSKC algorithm outperformed the others; RSKC is a modification of K-means clustering that robustly copes with high-dimensional data. Fifth, we validated findings in a data set of distinct individuals. Sixth, this unsupervised learning approach can be applied to detecting other medical conditions and thus is a generalizable advance in disease surveillance informatics. It is scalable and adaptable, and its ability to handle large data volumes may make it particularly effective for rare disease surveillance. Finally, a key novelty of this study is its minimal reliance on predefined criteria, allowing the data itself to guide discovery; although preprocessing decisions, such as variable selection, may never be fully optimal, embracing flexibility through unsupervised machine learning opens opportunities to learn something new.

The primary limitation of this study is that it presents only the initial steps in developing and analyzing our new approach to sepsis, including limiting consideration to an ICU cohort of subjects in a single jurisdiction. Thus, despite using highly diverse medical data of the sort routinely available for hospitalized patients, our results may not generalize to other patient cohorts or jurisdictions. Furthermore, our analysis based on fraction of ASE positivity within clusters can only be considered as preliminary, evidence of the potential value of this novel approach to sepsis. Second, despite best attempts, there are residual issues with data quality and completeness. Of the variables used in clustering, 41 (17.7%) contained missing values. However, the KMO and PCA variable pre-processing puts less focus on precision for each data point; it is also more practical for unsupervised tasks involving large data volumes than techniques such as multiple imputation. Finally, although we assessed eight different unsupervised clustering algorithms, we did not include others, such as hierarchical clustering and hierarchical density-based spatial clustering of applications with noise [67]. We also did not evaluate ensemble methods, or non-clustering methods, such as Latent Class Analysis [68] or Latent Dirichlet Allocation [69].

## Conclusions

The study is the first step towards a novel method of identifying and understanding sepsis through large-scale patient data. Next steps include expansion and fine-tuning of the list of input variables, *ex post facto* labelling by experts of all resulting clusters into clinical phenotypes expected to include a wide range of sepsis subphenotypes, application across population-based data sets representing different jurisdictions and cohorts with varied prevalence of underlying sepsis, and combined application across jurisdictions using federated learning methods.

## Supplementary Information

Supplementary Material 1

## Data Availability

The patient data are not available, given their potentially identifiable nature. Given the promising performance of the algorithm, we are currently applying it to the administrative and electronic health record data in other Canadian sites and cannot share the algorithm. We will share all the developed codes through GitHub once all ongoing projects are completed.

## References

[1] World Health Organization. Importance of surveillance in preventing and controlling noncommunicable diseases; 2023. https://www.emro.who.int/noncommunicable-diseases/publications/questions-and-answers-on-importance-of-surveillance-in-preventing-and-controlling-noncommunicable-diseases.html. Accessed 2 Dec 2023.

[2] World Health Organization. Noncommunicable disease surveillance, monitoring and reporting; 2023. https://www.who.int/images/default-source/departments/ncd-surveillance/global-monitoring-framework/gmf2-large.png?sfvrsn=30575e66_4. Accessed 2 Dec 2023.

[3] World Health Organization. Global report on the epidemiology and burden of sepsis: current evidence, identifying gaps and future directions; 2020. https://www.who.int/publications/i/item/9789240010789. Accessed 2 Dec 2023.

[4] Singer M, Deutschman CS, Seymour CW et al (2016) The third international consensus definitions for sepsis and septic shock (Sepsis-3). JAMA 315(8):801–810 26903338 10.1001/jama.2016.0287 PMC4968574

[5] Bone RC, Balk RA, Cerra FB, et al. Definitions for sepsis and organ failure and guidelines for the use of innovative therapies in sepsis. In: The ACCP/SCCM consensus conference committee. American College of Chest Physicians/Society of Critical Care Medicine. Chest*.* 1992;101(6):1644–1655. 10.1378/chest.101.6.1644 1303622

[6] Angus DC, Seymour CW, Coopersmith CM et al (2016) A framework for the development and interpretation of different sepsis definitions and clinical criteria. Crit Care Med 44(3):e113-121 26901559 10.1097/CCM.0000000000001730 PMC4765912

[7] Rhee C, Kadri SS, Danner RL et al (2016) Diagnosing sepsis is subjective and highly variable: a survey of intensivists using case vignettes. Crit Care 20:89 27048508 10.1186/s13054-016-1266-9 PMC4822273

[8] Garland A, Li N, Sligl W et al (2024) Adjudication of codes for identifying sepsis in hospital administrative data by expert consensus. Crit Care Med. 10.1097/CCM.0000000000006432 39637258 10.1097/CCM.0000000000006432 PMC11556841

[9] Jolley RJ, Quan H, Jette N et al (2015) Validation and optimisation of an ICD-10-coded case definition for sepsis using administrative health data. BMJ Open 5(12):e009487 26700284 10.1136/bmjopen-2015-009487 PMC4691777

[10] Iwashyna TJ, Odden A, Rohde J et al (2014) Identifying patients with severe sepsis using administrative claims: patient-level validation of the angus implementation of the international consensus conference definition of severe sepsis. Med Care 52(6):e39-43 23001437 10.1097/MLR.0b013e318268ac86 PMC3568444

[11] Rhee C, Zhang Z, Kadri SS et al (2019) Sepsis surveillance using adult sepsis events simplified eSOFA criteria versus sepsis-3 sequential organ failure assessment criteria. Crit Care Med 47(3):307–314 30768498 10.1097/CCM.0000000000003521 PMC6383796

[12] Centers for Disease Control and Prevention. Hospital toolkit for adult sepsis surveillance; 2018. https://www.cdc.gov/sepsis/pdfs/Sepsis-Surveillance-Toolkit-Mar-2018_508.pdf. Accessed 2 Dec 2023.

[13] Seymour CW, Coopersmith CM, Deutschman CS et al (2016) Application of a Framework to assess the usefulness of alternative sepsis criteria. Crit Care Med 44(3):e122-130 26901560 10.1097/CCM.0000000000001724 PMC4765919

[14] Heldens M, Schout M, Hammond NE, Bass F, Delaney A, Finfer SR (2018) Sepsis incidence and mortality are underestimated in Australian intensive care unit administrative data. Med J Aust 209(6):255–260 30176790 10.5694/mja18.00168

[15] Ma EY, Kim JW, Lee Y, Cho SW, Kim H, Kim JK (2021) Combined unsupervised-supervised machine learning for phenotyping complex diseases with its application to obstructive sleep apnea. Sci Rep 11(1):4457 33627761 10.1038/s41598-021-84003-4 PMC7904925

[16] Eckhardt CM, Madjarova SJ, Williams RJ et al (2023) Unsupervised machine learning methods and emerging applications in healthcare. Knee Surg Sports Traumatol Arthrosc 31(2):376–381 36378293 10.1007/s00167-022-07233-7

[17] Yu SC, Shivakumar N, Betthauser K et al (2021) Comparison of early warning scores for sepsis early identification and prediction in the general ward setting. JAMIA Open 4(3):ooab062 34820600 10.1093/jamiaopen/ooab062 PMC8607822

[18] Chen T, Guestrin C. XGBoost: A scalable tree boosting system. KDD’16: the 22nd ACM SIGKDD international conference on knowledge discovery and data mining; USA; 2016.

[19] Elixhauser A, Steiner C, Harris DR, Coffey RM (1998) Comorbidity measures for use with administrative data. Med Care 36(1):8–27 9431328 10.1097/00005650-199801000-00004

[20] Quan H, Sundararajan V, Halfon P et al (2005) Coding algorithms for defining comorbidities in ICD-9-CM and ICD-10 administrative data. Med Care 43(11):1130–1139 16224307 10.1097/01.mlr.0000182534.19832.83

[21] Kocak B (2022) Key concepts, common pitfalls, and best practices in artificial intelligence and machine learning: focus on radiomics. Diagn Interv Radiol 28(5):450–462 36218149 10.5152/dir.2022.211297 PMC9682557

[22] Li J, Yan XS, Chaudhary D et al (2021) Imputation of missing values for electronic health record laboratory data. NPJ Digit Med 4(1):147 34635760 10.1038/s41746-021-00518-0 PMC8505441

[23] Fan M, Peng X, Niu X, Cui T, He Q (2023) Missing data imputation, prediction, and feature selection in diagnosis of vaginal prolapse. BMC Med Res Methodol 23(1):259 37932660 10.1186/s12874-023-02079-0 PMC10629145

[24] Kaiser HF, Rice J (1974) Little Jiffy, Mark Iv. Educ Psychol Measur 34:111–117

[25] Jolliffe IT, Cadima J (2016) Principal component analysis: a review and recent developments. Philos Trans A Math Phys Eng Sci 374(2065):20150202 26953178 10.1098/rsta.2015.0202 PMC4792409

[26] Reimann C, Filzmoser P, Garrett RG, Dutter R (2008) Principal component analysis (PCA) and factor analysis (FA). In: Reimann C, Filzmoser P, Garrett RG, Dutter R (eds) Statistical data analysis explained. Wiley, pp 211–232

[27] Sigudla J, Maritz JE (2023) Exploratory factor analysis of constructs used for investigating research uptake for public healthcare practice and policy in a resource-limited setting, South Africa. BMC Health Serv Res 23(1):1423 38102600 10.1186/s12913-023-10165-8 PMC10724913

[28] Sürücü L, Yikilmaz İ, Maslakçi A. Exploratory factor analysis (EFA) in Quantitative researches and practical considerations. OSF Preprints, Center for Open Science.fgd4e.

[29] Selim SZ, Ismail MA (1984) K-means-type algorithms: a generalized convergence theorem and characterization of local optimality. IEEE Trans Pattern Anal Mach Intell 6(1):81–87 21869168 10.1109/tpami.1984.4767478

[30] Hicks SC, Liu R, Ni Y, Purdom E, Risso D (2021) mbkmeans: fast clustering for single cell data using mini-batch k-means. PLoS Comput Biol 17(1):e1008625 33497379 10.1371/journal.pcbi.1008625 PMC7864438

[31] Jing L, Ng MK, Huang JZ (2007) An entropy weighting k-means algorithm for subspace clustering of high-dimensional sparse data. IEEE Trans Knowl Data Eng 19(8):1026–1041

[32] Brodinová Š, Filzmoser P, Ortner T, Breiteneder C, Rohm M (2019) Robust and sparse k-means clustering for high-dimensional data. Adv Data Anal Classif 13(4):905–932

[33] Gaussian RD, Models M (2009). In: Li SZ, Jain A (eds) Encyclopedia of biometrics. Springer, US, Boston, pp 659–663

[34] Kaufman L, Rousseeuw P (1990) Clustering large applications (Program CLARA). In: Kaufman L, Rousseeuw P (eds) Finding groups in data. John Wiley & Sons Inc

[35] Kaski S (2011) Self-Organizing Maps. In: Sammut C, Webb GI (eds) Encyclopedia of machine learning. Springer, US, Boston, pp 886–888

[36] Shao W, Luo X, Zhang Z et al (2022) Application of unsupervised deep learning algorithms for identification of specific clusters of chronic cough patients from EMR data. BMC Bioinform 23(Suppl 3):140 10.1186/s12859-022-04680-4 PMC9019947 35439945

[37] Cui M (2020) Introduction to the K-means clustering algorithm based on the elbow method. Geosci Remote Sens 3:9–16

[38] Cattell RB (1966) The scree test for the number of factors. Multivariate Behav Res 1(2):245–276 26828106 10.1207/s15327906mbr0102_10

[39] Rousseeuw PJ (1987) Silhouettes: a graphical aid to the interpretation and validation of cluster analysis. J Comput Appl Math 20:53–65

[40] Davies DL, Bouldin DW (1979) A cluster separation measure. IEEE Trans Pattern Anal Mach Intell 1(2):224–227 21868852

[41] Caliński T, Harabasz J (1974) A dendrite method for cluster analysis. Commun Stat 3(1):1–27

[42] Konopinski MK (2020) Shannon diversity index: a call to replace the original Shannon’s formula with unbiased estimator in the population genetics studies. PeerJ 8:e9391 32655992 10.7717/peerj.9391 PMC7331625

[43] Lee WC (1997) Characterizing exposure-disease association in human populations using the Lorenz curve and Gini index. Stat Med 16(7):729–739 9131761 10.1002/(sici)1097-0258(19970415)16:7<729::aid-sim491>3.0.co;2-a

[44] Liu T, Yu H, Blair RH (2022) Stability estimation for unsupervised clustering: a review. Wiley Interdiscip Rev Comput Stat 14(6):e1575 36583207 10.1002/wics.1575 PMC9787023

[45] Dudoit S, Fridlyand J (2002) A prediction-based resampling method for estimating the number of clusters in a dataset. Genome Biol 3(7):RESEARCH0036 12184810 10.1186/gb-2002-3-7-research0036 PMC126241

[46] Lange T, Roth V, Braun ML, Buhmann JM (2004) Stability-based validation of clustering solutions. Neural Comput 16(6):1299–1323 15130251 10.1162/089976604773717621

[47] Steinley D (2004) Properties of the Hubert-Arabie adjusted Rand index. Psychol Methods 9(3):386–396 15355155 10.1037/1082-989X.9.3.386

[48] Knops ZF, Maintz JB, Viergever MA, Pluim JP (2006) Normalized mutual information based registration using k-means clustering and shading correction. Med Image Anal 10(3):432–439 16111913 10.1016/j.media.2005.03.009

[49] Eisenman RL (1967) A profit-sharing interpretation of Shapley value for N-person games. Behav Sci 12(5):396–398 6059772 10.1002/bs.3830120506

[50] Canadian Institute for Health Information. Indicator Library: Diagnosis type definitions; 2016. https://www.cihi.ca/sites/default/files/document/diagnosis-type-definitions-en.pdf. Accessed 28 Nov 2023.

[51] Canadian Institute for Health Information. International Statistical Classification of Diseases and Related Health Problems, Tenth Revision, Canada; 2018. p. 1–761.

[52] Juurlink D, Preyra C, Croxford R et al. Canadian Institute for health information discharge abstract database: a validation study. ICES; 2006.

[53] Misset B, Nakache D, Vesin A et al (2008) Reliability of diagnostic coding in intensive care patients. Crit Care 12(4):R95 18664267 10.1186/cc6969 PMC2575581

[54] Hemphill JF (2003) Interpreting the magnitudes of correlation coefficients. Am Psychol 58(1):78–79 12674822 10.1037/0003-066x.58.1.78

[55] Snoek J, Larochelle H, Adams RP. Practical Bayesian optimization of machine learning algorithms. Advances in neural information processing systems 25 (NIPS 2012); 2012.

[56] Van Calster B, McLernon DJ, van Smeden M et al (2019) Calibration: the Achilles heel of predictive analytics. BMC Med 17(1):230 31842878 10.1186/s12916-019-1466-7 PMC6912996

[57] Elixhauser A, Owens P. Reasons for being admitted to the hospital through the emergency department, 2003. In: Paper presented at: healthcare cost and utilization project (HCUP) statistical briefs; 2006. Rockville (MD). 21938851

[58] Garland A, Olafson K, Ramsey CD, Yogendran M, Fransoo R (2013) Epidemiology of critically ill patients in intensive care units: a population-based observational study. Crit Care 17(5):R212 24079640 10.1186/cc13026 PMC4056438

[59] Biebelberg B, Rhee C, Chen T, McKenna C, Klompas M (2024) Heterogeneity of Sepsis presentations and mortality rates. Ann Intern Med 177(7):985–987 38801775 10.7326/M24-0400

[60] Antcliffe DB, Mi Y, Santhakumaran S et al (2024) Patient stratification using plasma cytokines and their regulators in sepsis: relationship to outcomes, treatment effect and leucocyte transcriptomic subphenotypes. Thorax 79(6):515–523 38471792 10.1136/thorax-2023-220538 PMC11137467

[61] Taylor SP, Bray BC, Chou SH, Burns R, Kowalkowski MA (2022) Clinical subtypes of sepsis survivors predict readmission and mortality after hospital discharge. Ann Am Thorac Soc 19(8):1355–1363 35180373 10.1513/AnnalsATS.202109-1088OC PMC9353958

[62] Fohner AE, Greene JD, Lawson BL et al (2019) Assessing clinical heterogeneity in sepsis through treatment patterns and machine learning. J Am Med Inform Assoc 26(12):1466–1477 31314892 10.1093/jamia/ocz106 PMC7647146

[63] Seymour CW, Kennedy JN, Wang S et al (2019) Derivation, validation, and potential treatment implications of novel clinical phenotypes for sepsis. JAMA 321(20):2003–2017 31104070 10.1001/jama.2019.5791 PMC6537818

[64] Gardlund B, Dmitrieva NO, Pieper CF, Finfer S, Marshall JC, Taylor TB (2018) Six subphenotypes in septic shock: latent class analysis of the PROWESS Shock study. J Crit Care 47:70–79 29933169 10.1016/j.jcrc.2018.06.012 PMC7416734

[65] DeMerle KM, Kennedy JN, Chang CH et al (2024) Identification of a hyperinflammatory sepsis phenotype using protein biomarker and clinical data in the ProCESS randomized trial. Sci Rep 14(1):6234 38485953 10.1038/s41598-024-55667-5 PMC10940677

[66] Jaimes FA, De La Rosa GD, Valencia ML et al (2013) A latent class approach for sepsis diagnosis supports use of procalcitonin in the emergency room for diagnosis of severe sepsis. BMC Anesthesiol 13(1):23 24050481 10.1186/1471-2253-13-23 PMC3850719

[67] Ye JY, Yu C, Husman T, Chen B, Trikala A (2021) Novel strategy for applying hierarchical density-based spatial clustering of applications with noise towards spectroscopic analysis and detection of melanocytic lesions. Melanoma Res 31(6):526–532 34494605 10.1097/CMR.0000000000000771 PMC8568327

[68] Sinha P, Calfee CS, Delucchi KL (2021) Practitioner’s guide to latent class analysis: methodological considerations and common pitfalls. Crit Care Med 49(1):e63–e79 33165028 10.1097/CCM.0000000000004710 PMC7746621

[69] Wang Y, Zhao Y, Therneau TM et al (2020) Unsupervised machine learning for the discovery of latent disease clusters and patient subgroups using electronic health records. J Biomed Inform 102:103364 31891765 10.1016/j.jbi.2019.103364 PMC7028517

