# Supplementary material for: Unsupervised clustering for sepsis identification in large-scale patient data: a model development and validation study
Source: Intensive Care Med Exp. 2025 Mar 20;13:37. doi: 10.1186/s40635-025-00744-w (PMC11925832; doi:10.1186/s40635-025-00744-w)
Supplement: Supplementary file 1 — Supplementary Material 1 [file 40635_2025_744_MOESM1_ESM.docx]

**Unsupervised Clustering for Sepsis Detection in Electronic Health Records: A Model Development and Validation Study**

**Supplementary Materials**

Table of Contents

[Appendix A 2](#_Toc190082557)

[**eFigure A1.** Cluster performance evaluation strategy. 2](#_Toc190082558)

[**eTable A1.** Data sources and elements. 3](#_Toc190082559)

[**eTable A2.** Comparison of the eight clustering algorithms. 4](#_Toc190082560)

[**eTable A3.** R functions and parameters of the clustering algorithms used for the analyses. 5](#_Toc190082561)

[**eTable A4.** Optimized code-based algorithm for sepsis^13^. 6](#_Toc190082562)

[Appendix B. Additional Results for Model Interpretation 9](#_Toc190082563)

[**eTable B1.** Patient characteristics of patient cohorts. Values are n (%) unless indicated. 9](#_Toc190082564)

[**eTable B2.** Comparing clustering algorithms in the development cohort. 10](#_Toc190082565)

[**eFigure B1.** Elbow plot for the optimal number of clusters (k) for Robust and Sparse K-means Clustering (RSKC). 11](#_Toc190082566)

[**eTable B3.** Development cohort patient characteristics between ASE(+) (Adult Sepsis Event) and ASE(-) cases within the 11 patient clusters with ≥50% ASE(+) members (the ASE-majority clusters). Values are n (%) unless otherwise indicated. 12](#_Toc190082567)

[**eFigure B2.** Subsampling stability assessment for the best-performing algorithm among the top two clustering methods, K-Means and Robust and Sparse K-means Clustering (RSKC), based on Table 2. 13](#_Toc190082568)

[**eTable B4.** Feature importance using SHapley Additive exPlanations (SHAP)^16^ values derived from XGBoost^17^ for clusters identified by Robust and Sparse K-means Clustering (RSKC) in the development cohort. 14](#_Toc190082569)

[**eTable B5.** Most common categories of Most Responsible Hospital Diagnoses (MRHD), by cluster. 17](#_Toc190082570)

[**eFigure B3.** Feature importance heatmap using SHapley Additive exPlanations (SHAP) values derived from XGBoost for clusters identified by Robust and Sparse K-means Clustering (RSKC) for the development cohort, in descending left-to-right order of fraction meeting Adult Sepsis Event definition. 18](#_Toc190082571)

[**eTable B6.** Patient characteristics between the ASE-majority clusters and the ASE-minority clusters in the development cohort. Values are n (%) unless otherwise indicated. 19](#_Toc190082572)

[**eTable B7.** Patient characteristics for the 11 ASE-majority clusters in the development cohort. Patients identified by Robust and Sparse K-means Clustering (RSKC). Values are n (%) unless indicated. 21](#_Toc190082573)

[**eFigure B4.** Principal Component Analysis (PCA) plot with ASE-majority clusters and ASE-minority clusters identified by Robust and Sparse K-means Clustering (RSKC) for the development cohort. 24](#_Toc190082574)

[**eFigure B5.** Laboratory test result distributions between ASE(+) [Adult Sepsis Event] and ASE(-) cases among the sepsis patients identified by Robust and Sparse K-means Clustering (RSKC) from the development cohort. 26](#_Toc190082575)

[**eFigure B6.** Laboratory test result distributions between ASE(+) [Adult Sepsis Event] and ASE(-) cases among the sepsis patients identified by Robust and Sparse K-means Clustering (RSKC) from the validation cohort. 27](#_Toc190082576)

[**eFigure B7.** Percentages of ASE(+) (Adult Sepsis Event) cases in the 48 clusters for the development (x-axis) and validation (y-axis) cohorts. 28](#_Toc190082577)

[**eTable B8.** Patient characteristics of the ASE(+) [Adult Sepsis Event] patients in the 11 ASE-majority clusters (ASEmac) versus the 37 ASE-minority clusters (ASEmic) in the development cohort. Values are n (%) unless otherwise indicated. 29](#_Toc190082578)

[**eTable B9.** Independent, blinded clinical evaluation and categorization of the 48 clusters from the development cohort into eight clinical categories using cluster-specific summary statistics of patient characteristics. 30](#_Toc190082579)

[**eTable B10.** Patient characteristics among the 11 ASE-majority clusters between the development and validation cohort. Values are n (%) unless otherwise indicated. 32](#_Toc190082580)

[Appendix C. Handling Missing Values and Variable Description 33](#_Toc190082581)

[**eTable C1.** Methods for missing data imputation. The mean imputation method was used in our unsupervised learning approach, primarily for missing patient-specific laboratory test results. 33](#_Toc190082582)

[**eTable C2.** Description of the 592 processed variables and the 231 variables included in the model development. 34](#_Toc190082583)

[References 49](#_Toc190082584)

# Appendix A

## **eFigure A1.** Cluster performance evaluation strategy.

**
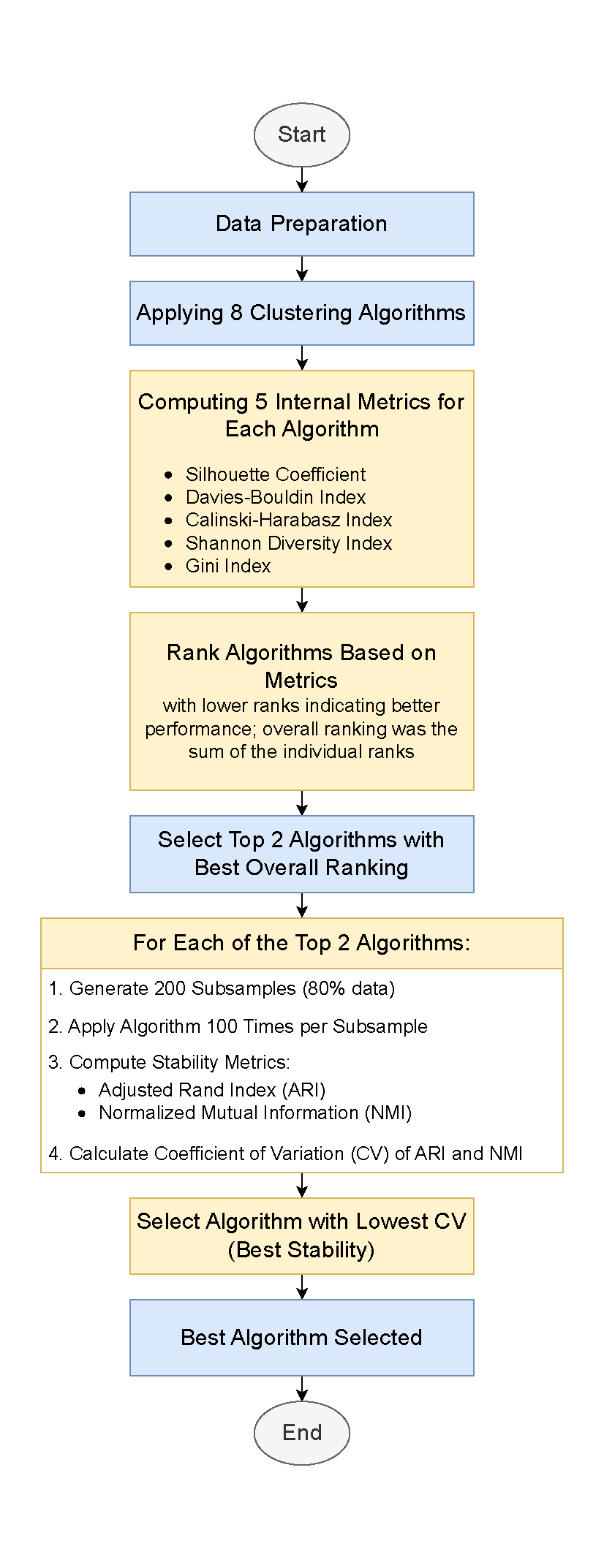
**

## **eTable A1.** Data sources and elements.

| **Data Source** | **Table Name** | **Description** | **Variables Included** |
| --- | --- | --- | --- |
| **DAD** ^1^ | | Administrative records of hospital discharges, focusing on patient demographics, clinical diagnoses, and outcomes. | De-identified patient identifier, hospitalization dates/times, age, sex, hospital identifier, postal code, admission source, diagnoses (up to 25), procedures (up to 20), discharge disposition. |
| **NACRS** ^2^ | | Ambulatory care data, including emergency department visits, day surgeries, and outpatient clinics. | Visit date, patient demographics, visit diagnoses, treatments. |
| **SCM EMR** ^3^ | Medication | Inpatient medication administration. | Medication names, dosages, administration times. |
|  | Vital Signs | Measured values | Temperature, blood pressure, heart rate, respiratory rate, oxygen saturation. |
|  | Laboratory tests | Values of tests conducted on inpatients. | Test, date and time of test, results, normal ranges, critical values. |
|  | Transfusions | Blood products administered. | Type, issue date and time, volume. |
| **eCritical Alberta** ^4^ | | Data on to patients admitted to the Intensive Care Unit (ICU). | ICU admission and discharge dates/times, care details (e.g., mechanical ventilation), SOFA scores, disposition. |

DAD: Discharge Abstract Database; NACRS: National Ambulatory Care Reporting System; SCM EMR: Sunrise Clinical Manager (SCM) Electronic Medical Record (EMR) database; SOFA, Sequential Organ Failure Assessment.

## **eTable A2.** Comparison of the eight clustering algorithms.

| **Algorithm** | **Base method** | **Typical cluster shape** | **Advantages** | **Ideal usage scenario** | **Drawbacks** |
| --- | --- | --- | --- | --- | --- |
| K-Means ^5^ | Centroid-based clustering | Spherical | Simple, fast | Large datasets with clear separation | Sensitive to initial centroid placement |
| Gaussian mixture model (GMM) ^6^ | Probabilistic K-Means | Elliptical | Can model different cluster shapes | Non-spherical clusters | Slower due to Expectation-Maximization |
| Clustering large Applications (CLARA) ^7^ | Sampling-based Partitioning Around Medoids (PAM) | Various | Can handle large datasets through sampling | Very large datasets | Requires pre-specification of clusters and samples |
| Mini Batch K-Means ^8^ | K-Means with mini-batches | Spherical | Scalable and faster convergence | Need for speed with large datasets | Might be less accurate than standard K-Means |
| Weighted K-Means ^9^ | K-Means with data point weights | Spherical | Flexibility with weighted data points | Unequal data point importance | Complexity in defining weights |
| Robust and Sparse K-Means (RSKC) ^10^ | K-Means with robustness & feature selection | Spherical | Resistant to outliers, performs feature selection | Noisy data or irrelevant features | Computationally intensive |
| Self-Organized Map (SOM) ^11^ | Neural network-based clustering | Topology-preserving, various shapes | Preserves topological properties, good for high-dimensional data visualization | Dimensionality reduction, visualizing complex structures | Computationally intensive, requires predefined map size, initial weights and data order affect output |
| Regularized Deep Clustering with Auto-Encoder (Regularized DCAE) ^12^ | Deep learning-based clustering using auto-encoders | Flexible, often complex and non-linear shapes | Efficient at handling large datasets, capable of learning complex and non-linear data representations, automatically learn feature representations, reducing the need for manual feature engineering | Large and high-dimensional datasets, automatic feature extraction and representation learning | Requires significant computational resources, prone to overfitting (especially with smaller datasets), challenging to optimize model architecture and hyperparameters |

## **eTable A3.** R functions and parameters of the clustering algorithms used for the analyses.

| **Algorithm** | **Function Used** | **Parameters** | **Parameters Used** |
| --- | --- | --- | --- |
| K-Means | kmeans(…) under `stats` | - centers: The number of clusters (k). - iter.max: The maximum number of iterations allowed. - nstart: The number of random starting partitions when `centers` are a number. | - `centers` selected based on the number of input variables/components. - iter.max = 20 - nstart = 25 |
| Gaussian Mixture Model (GMM) | GMM(…) in the `ClusterR` package | - gaussian_comps: The number of gaussian mixture components - dist_mode: The distance used during the seeding of initial means and k-means clustering - seed_mode: How the initial means are seeded prior to running k-means and/or EM algorithms. | - `gaussian_comps` selected based on the number of input variables/components. - dist_mode = "eucl_dist" - seed_mode = "random_subset" |
| Clustering Large Applications (CLARA) | clara(…) in the `cluster` package | - k: The number of clusters. - metric: The metric to be used for calculating dissimilarities between observations. | - `k` selected based on the number of input variables/components. - metric = "euclidean" |
| Mini Batch K-Means | MiniBatchKmeans(…) in the `ClusterR` package | - clusters: The number of clusters. - batch_size: The size of the mini batches. - num_init: Number of times the algorithm will be run with different centroid seeds. - max_iters: The maximum number of clustering iterations. - early_stop_iter: Continue that many iterations after calculation of the best within-cluster-sum-of-squared-error. | - `clusters` selected based on the number of input variables/components. - batch_size = 200 - num_init = 10 - max_iters =100 - early_stop_iter = 10 |
| Entropy Weighted K-Means | ewkm(…) in the `wskm` package | - clusters: The number of clusters. - lambda: Parameter for variable weight distribution. - max_iters: The maximum number of clustering iterations. - delta: Maximum change allowed between iterations for convergence. | - `clusters` selected based on the number of input variables/components. - lambda = 0.5 - max_iters =100 - delta = 0.00001 |
| Robust and Sparse K-means Clustering (RSKC) | RSKC(…) in the `RSKC` package | - ncl: The number of clusters. - alpha: 0 <= alpha <= 1, the proportion of the cases to be trimmed in robust sparse K-means. - L1: A single L1 bound on weights (the feature weights). - nstart: The number of random starting partitions when `centers` are a number. | - `clusters` selected based on the number of input variables/components. - alpha = 1/6 - L1 = 6 - nstart = 25 |
| Self-Organized Map (SOM) | som(…) in the `kohonen` package | - grid: Defines the dimensions of the SOM grid. - rlen: The number of times the complete dataset will be presented to the network. - alpha: A vector of two learning rates that linearly decrease over rlen. - radius: The initial and final radius of the neighborhood function. | - grid size typically chosen based on dataset characteristics and visualization needs. - rlen = 100 (may vary depending on dataset size). - alpha usually starts at 0.05 and linearly decreases. - radius typically starts large to affect many neurons and decrease over time. |
| Regularized Deep Clustering with Auto-Encoder (Regularized DCAE) | This algorithm involves multiple steps, each using different functions from the `keras` package: keras_model_sequential(), layer_dense(), layer_batch_normalization() for building the encoder and decoder. keras_model() for creating the autoencoder model. compile() and fit() functions to compile and train the model. | - layers_dense: Defines the architecture of the encoder and decoder parts of the auto-encoder. - keras_model and compile: Configures a Keras model with auto-encoder for training. - keras::fit: Trains the model with defined number of samples per gradient update (batch_size), number of epochs, fraction of data for validation (validation_split), early stopping callbacks, and other hyperparameters. | - layers_dense: Each layer (encoder or decoder) with activation = 'tanh', units = 20, input_shape set to the number of the columns of the input matrix, kernel_regularizer set to ` regularizer_l1_l2(l1 = 0.001, l2 = 0.001)`. - keras_model and compile: inputs as the encoder layers and outputs as the decoder layers. optimizer = 'adam' and loss = 'mean_squared_error'. - keras::fit: batch_size = 16, epochs = 200, validation_split = 0.2. |

## **eTable A4.** Optimized code-based algorithm for sepsis^13^.

| **ICD-10-CA/CCI Codes** | **Description** |
| --- | --- |
| ***Infection Codes*** | |
| A02.1 | Salmonella sepsis |
| A03.9 | Shigellosis, unspecified |
| A04.7 | Enterocolitis due to Clostridium difficile |
| A20.7 | Septicaemic plague |
| A21.7 | Generalized tularaemia |
| A22.7 | Anthrax sepsis |
| A23.9 | Brucellosis, unspecified |
| A24.1 | Acute and fulminating melioidosis |
| A26.7 | Erysipelothrix sepsis |
| A28.0 | Pasteurellosis |
| A28.2 | Extraintestinal yersiniosis |
| A32.7 | Listerial sepsis |
| A39.2 | Acute meningococcaemia |
| A39.3 | Chronic meningococcaemia |
| A39.4 | Meningococcaemia, unspecified |
| A40.x | Streptococcal sepsis |
| A41.x | Other sepsis |
| A42.7 | Actinomycotic sepsis |
| B00.7 | Disseminated herpesviral disease |
| B37.7 | Candidal sepsis |
| B95.48 | Other Streptococcus as the cause of diseases classified to other chapters |
| B95.6 | Staphylococcus aureus as the cause of diseases classified elsewhere |
| B96.2 | E. coli as the cause of diseases classified to other chapters |
| J18.9 | Pneumonia, unspecified |
| J44.0 | Chronic obstructive pulmonary disease with acute lower respiratory infection |
| N39.0 | Urinary tract infection, site not specified |
| P35.2 | Congenital herpesviral [herpes simplex] infection |
| P36.0 | Sepsis of newborn due to streptococcus, group B |
| P36.1 | Sepsis of newborn due to other and unspecified streptococci |
| P36.2 | Sepsis of newborn due to Staphylococcus aureus |
| P36.3 | Sepsis of newborn due to other and unspecified staphylococci |
| P36.4 | Sepsis of newborn due to Escherichia coli |
| P36.5 | Sepsis of newborn due to anaerobes |
| P36.8 | Other bacterial sepsis of newborn |
| P36.9 | Bacterial sepsis of newborn, unspecified |
| P37.2 | Neonatal (disseminated) listeriosis |
| P37.5 | Neonatal candidiasis |
| ***Organ Failure Codes*** | |
| R57.2 | Septic Shock: This code represents sepsis without also requiring an infection code |
| D65.x | Disseminated Intravascular Coagulation |
| D69.5 | Secondary Thrombocytopenia |
| D69.6 | Thrombocytopenia Unspecified |
| F05.0 | Delirium Not Superimposed on Dementia, so described |
| F05.9 | Delirium Unspecified |
| G93.1 | Anoxic Brain Damage Not elsewhere classified |
| G93.4 | Encephalopathy, Unspecified |
| G93.80 | Metabolic Encephalopathy |
| I95.1 | Orthostatic Hypotension |
| I95.9 | Hypotension, Unspecified |
| J80.x | Adult Respiratory Distress Syndrome |
| J96.0 | Acute Respiratory Failure |
| J96.9 | Respiratory Failure, Unspecified |
| K72.0 | Acute And Subacute Hepatic Failure |
| K72.9 | Hepatic Failure, Unspecified |
| K76.3 | Infarction Of Liver |
| N17.0 | Acute Renal Failure with Tubular Necrosis |
| N17.1 | Acute Renal Failure with Acute Cortical Necrosis |
| N17.2 | Acute Renal Failure with Medullary Necrosis |
| N17.8 | Other Acute Renal Failure |
| N17.9 | Acute Renal Failure, Unspecified |
| R09.2 | Respiratory Arrest |
| R57.0 | Cardiogenic Shock |
| R57.1 | Hypovolaemic Shock |
| R57.8 | Other Shock |
| R57.9 | Shock, Unspecified |
| 1GZ31CAND | Ventilation, respiratory system NEC, invasive per orifice approach by (endotracheal) intubation, positive pressure |
| 1GZ31CRND | Ventilation, respiratory system NEC, invasive per orifice with incision approach for intubation through tracheostomy, positive pressure |
| 1GZ31GPND | Ventilation, respiratory system, invasive per orifice per trans-tracheal needle |

CCI: Canadian Classification of Interventions; NEC: not elsewhere classified; x: any number of subsequent wildcard characters.

**eTable A5.**  Relaxed thresholds for Adult Sepsis Event identification of sepsis ^14^.

| ***Parameter*** | ***Original threshold*** | ***Modified threshold*** |
| --- | --- | --- |
| Serum lactate (mM) ^15^ | ≥2.0 | ≥1.5 |
| Serum creatinine, rise over baseline ^16^ | >200% | >150% |
| Platelet count ^17-19^  value (x 10^9^/L)  fall from baseline | <100  >50% | <150  >25% |
| Total bilirubin ^20,21^  value (mM)  rise over baseline | 34.2  >200% | 25.7  >150% |
| Interval (days) between blood culture and first acute organ dysfunction | ≤2 | ≤3 or 4 |
| Days of antimicrobial administration | >4 | >3 |

# Appendix B. Additional Results for Model Interpretation

## **eTable B1.** Patient characteristics of patient cohorts. Values are n (%) unless indicated.

| Characteristic  N (%) | Development cohort  April 2015 - March 2016  N = 3,660 | External validation cohort  April 2016 - March 2017  N = 3,012 | Standardized difference between the two cohorts |
| --- | --- | --- | --- |
| Age, median (interquartile range [IQR]) | 62 (52 – 73) | 62 (52 – 72) | 0.03 |
| Female | 1,315 (36) | 956 (32) | 0.09 |
| ***Chronic comorbid conditions^1^*** | | | |
| Congestive Heart Failure | 535 (15) | 369 (12) | -0.07 |
| Cardiac Arrhythmia | 924 (25) | 723 (24) | -0.03 |
| Valvular Disease | 356 (9.7) | 299 (9.9) | 0.01 |
| Pulmonary Circulation Disorders | 138 (3.8) | 109 (3.6) | -0.01 |
| Peripheral Vascular Disorders | 154 (4.2) | 141 (4.7) | 0.02 |
| Hypertension, Uncomplicated | 1,765 (48) | 1,415 (47) | -0.02 |
| Hypertension, Complicated | 15 (0.4) | 13 (0.4) | 0 |
| Paralysis | 62 (1.7) | 33 (1.1) | -0.05 |
| Other Neurological Disorders | 225 (6.1) | 181 (6.0) | -0.01 |
| Chronic Pulmonary Disease | 261 (7.1) | 188 (6.2) | -0.04 |
| Diabetes, Uncomplicated | 247 (6.7) | 176 (5.8) | -0.04 |
| Diabetes, Complicated | 802 (22) | 614 (20) | -0.04 |
| Hypothyroidism | 41 (1.1) | 25 (0.8) | -0.03 |
| Renal Failure | 104 (2.8) | 79 (2.6) | -0.01 |
| Liver Disease | 148 (4.0) | 116 (3.9) | -0.01 |
| Peptic Ulcer Disease, excluding bleeding | 17 (0.5) | 7 (0.2) | -0.04 |
| AIDS/HIV | 3 (<0.1) | 2 (<0.1) | -0.01 |
| Lymphoma | 26 (0.7) | 13 (0.4) | -0.04 |
| Metastatic Cancer | 45 (1.2) | 45 (1.5) | 0.02 |
| Solid Tumor without Metastasis | 131 (3.6) | 105 (3.5) | -0.01 |
| Rheumatoid Arthritis/collagen-vascular disease | 42 (1.1) | 37 (1.2) | 0.01 |
| Coagulopathy | 148 (4.0) | 115 (3.8) | -0.01 |
| Obesity | 128 (3.5) | 104 (3.5) | 0 |
| Weight Loss | 32 (0.9) | 26 (0.9) | 0 |
| Fluid and Electrolyte Disorders | 395 (11) | 352 (12) | 0.03 |
| Blood Loss Anemia | 9 (0.2) | 9 (0.3) | 0.01 |
| Deficiency Anemia | 49 (1.3) | 44 (1.5) | 0.01 |
| Alcohol Abuse | 258 (7.0) | 196 (6.5) | -0.02 |
| Drug Abuse | 105 (2.9) | 72 (2.4) | -0.03 |
| Psychoses | 16 (0.4) | 15 (0.5) | 0.01 |
| Depression | 102 (2.8) | 65 (2.2) | -0.04 |
| End-Stage Renal Disease | 46 (1.3) | 24 (0.8) | -0.05 |
| ***Clinical characteristics during hospitalization*** | | | |
| Admitted via Emergency Department | 2,430 (66) | 1,944 (65) | 0.04 |
| ICU length of stay, median (IQR) | 3 (2 – 5) | 3 (2 – 5) | 0.04 |
| Hospital length of stay, days, median (IQR) | 8 (5 – 17) | 7 (4 – 15) | 0.09 |
| Received invasive mechanical ventilation | 1,427 (39) | 1,198 (40) | 0.02 |
| Blood culture collected | 1,348 (37) | 960 (32) | -0.10 |
| Received antimicrobial medications  - for 2 or more consecutive days | 2,163 (59)  1,979 (54) | 1,682 (56)  1,528 (51) | -0.07  -0.07 |
| Received intravenous vasopressor therapy | 684 (19) | 481 (16) | -0.07 |
| Highest serum lactate (in mmol/L), median (IQR) | 2.6 (1.6 – 4.8) | 2.6 (1.7 – 4.7) | 0.02 |
| Highest serum creatinine (in μmol/L), median (IQR) | 95 (77 – 128) | 97 (80 – 123) | 0.03 |
| Highest serum total bilirubin (in μmol/L), median (IQR) | 11 (7 – 19) | 11 (7 – 18) | 0.05 |
| Lowest serum platelet count (in 10^9^/L), median (IQR) | 166 (120 – 212) | 168 (121 – 212) | -0.02 |
| In-hospital mortality | 419 (11) | 320 (11) | -0.03 |
| ***Sepsis cases by comparator definitions*** | | | |
| Adult Sepsis Event (ASE) definition | 787 (21.5) | 576 (19.1) | -0.06 |

Thresholds for the absolute values of standardized differences: 0≤d≤0.2 negligible effect size; 0.2<d≤0.4 small effect size (*); 0.4<d≤0.8 medium effect size (**); d>0.8 large effect size (***).

^1^The presence of the 31 chronic comorbid conditions was determined using the Elixhauser Comorbidity Index, defined from ICD-10-CA codes as per the methodology outlined by Hude Quan et al. ^22^.

## **eTable B2.** Comparing clustering algorithms in the development cohort.

| **Method** | **# of principal components** | **Optimal # of clusters** | **% of ASE(+) cases in the clusters**  **median (min – max)** | **# of sepsis clusters by ASE(+) ≥50%** |
| --- | --- | --- | --- | --- |
| K-Means | 55 | 48 | 28.4 (0, 100) | 19 |
| GMM | 55 | 48 | 24.1 (0, 87.5) | 15 |
| CLARA | 55 | 48 | 62.2 (0.4, 100) | 30 |
| Mini Batch K-Means | 55 | 48 | 63.4 (0, 100) | 25 |
| Weighted K-Means | 55 | 48 | 16.0 (0, 100) | 3 |
| RSKC | 55 | 48 | 6.0 (0, 86.9) | 11 |
| SOM | 55 | 48 | 23.4 (0, 92.8) | 15 |
| Regularized DCAE | N/A | 48 | 16.7 (0, 93.6) | 17 |

ASE: Adult Sepsis Event definition of sepsis; GMM: Gaussian Mixture Model; CLARA: Clustering large Applications; RSKC: Robust and Sparse K-means Clustering; SOM: Self-Organized Map; DCAE: Deep Clustering with Auto-Encoder.

## **eFigure B1.** Elbow plot for the optimal number of clusters (k) for Robust and Sparse K-means Clustering (RSKC).


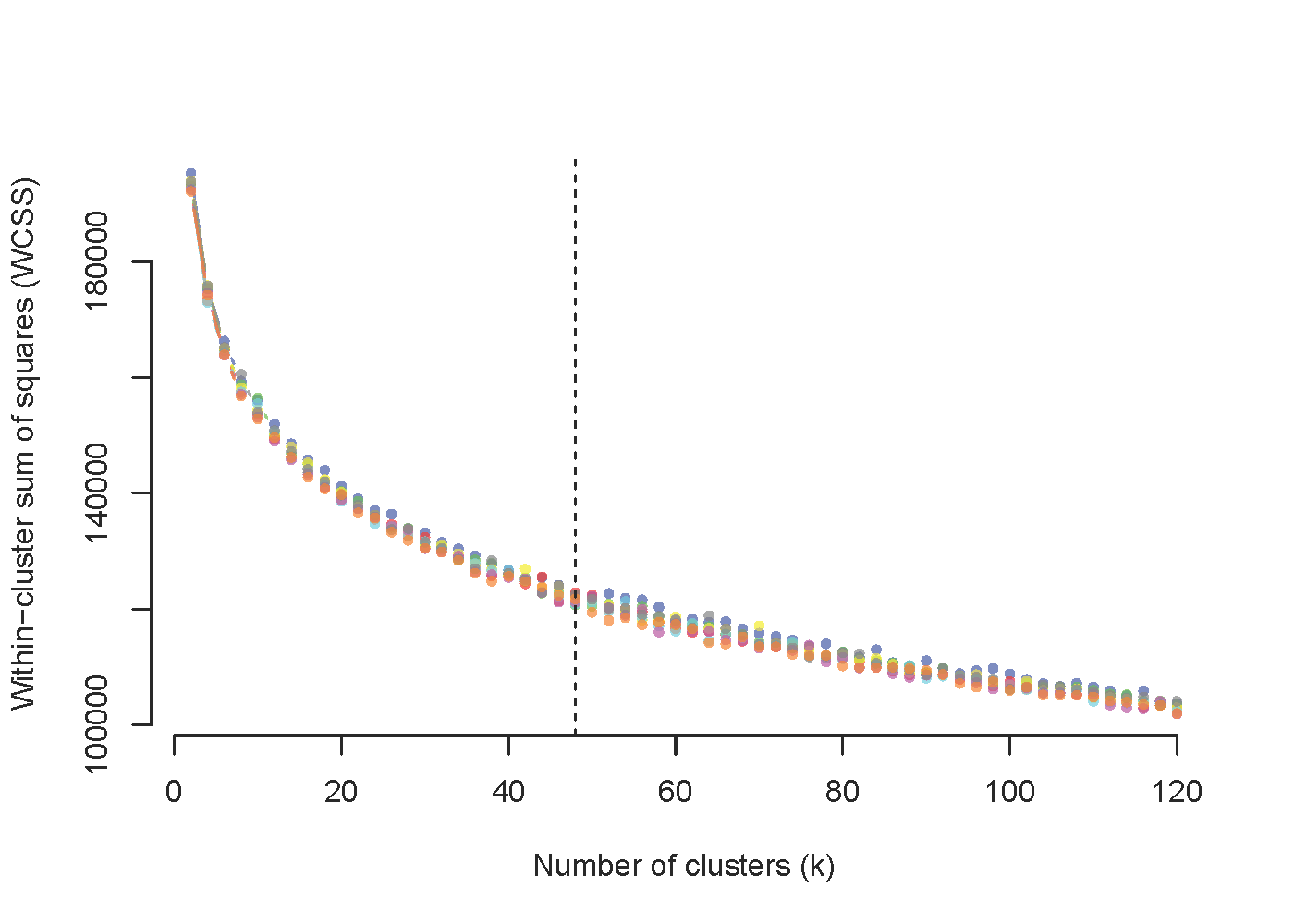


The dashed black line marks the selected cutoff for k, k = 48.

The multiple lines in the figure represent results from different initializations of the RSKC algorithm, tested to ensure fair comparison with other clustering algorithms that may be sensitive to starting points.

We observed minimal differences in the within-cluster sum of squares (WCSS) across initializations, suggesting that RSKC is relatively insensitive to starting points. Furthermore, WCSS values became slightly more dispersed beyond k = 48, indicating increased variability in clustering outcomes at higher k values.

Based on the figure, the elbow point could fall within a range of k values, likely between 40 and 60, rather than at a single precise point. The elbow method suggests looking for a point where adding more clusters yields diminishing returns, i.e., the rate of decrease in WCSS slows significantly. We selected k = 48 as it lies at the point where WCSS begins to stabilize with minimal dispersion across initializations. This choice balances cluster compactness and stability, aligning with the goals of RSKC.

## **eTable B3.** Development cohort patient characteristics between ASE(+) (Adult Sepsis Event) and ASE(-) cases within the 11 patient clusters with ≥50% ASE(+) members (the ASE-majority clusters). Values are n (%) unless otherwise indicated.

| **Characteristic** | **All**  **N = 821** | **ASE(-)**  **N = 209** | **ASE(+)**  **N = 612** | **Standardized difference** |
| --- | --- | --- | --- | --- |
| Age, median (interquartile range [IQR]) | 61 (51 – 72) | 61 (52 – 75) | 61 (50 – 71) | 0.09 |
| Female | 351 (42.8) | 83 (39.7) | 268 (43.8) | 0.08 |
| ***Clinical characteristics during hospitalization*** | | | | |
| Admitted via Emergency Department | 651 (79.3) | 147 (70.3) | 504 (82.4) | -0.28* |
| ICU length of stay, median (IQR) | 7 (4 – 12) | 6 (3 – 11) | 7 (4 – 12) | -0.22* |
| Hospital length of stay, median (IQR) | 19 (9 – 36) | 21 (11 – 39) | 19 (8 – 34) | 0.14 |
| Received invasive mechanical ventilation (IMV) | 578 (70.4) | 138 (66.0) | 440 (71.9) | 0.13 |
| Blood culture collected | 779 (94.9) | 167 (79.9) | 612 (100.0) | 0.93** |
| - Days from admission to blood culture, median (IQR) | 0 (0 – 2) | 3 (0 – 7) | 0 (0 – 1) | 0.52** |
| Received antimicrobial medications | 814 (99.1) | 202 (96.7) | 612 (100.0) | 0.37* |
| - for 2 or more consecutive days | 810 (98.7) | 198 (94.7) | 612 (100.0) | 0.46** |
| Died during hospitalization | 255 (31.1) | 54 (25.8) | 201 (32.8) | 0.15 |
| ***Acute organ dysfunction per ASE criteria: (Any one of the following ± 2 days of blood culture)*** | | | | |
| Number of acute organ dysfunctions, median (IQR) | 3 (2 – 4) | 2 (1 – 3) | 3 (2 – 4) | -0.69** |
| Respiratory (invasive mechanical ventilation) | 432 (52.6) | 87 (41.6) | 345 (56.4) | 0.30* |
| Renal | 452 (55.1) | 93 (44.5) | 359 (58.7) | 0.28* |
| Liver | 135 (16.4) | 21 (10.0) | 114 (18.6) | 0.25* |
| Hematologic | 291 (35.4) | 61 (29.2) | 230 (37.6) | 0.18 |
| Cardiac dysfunction (received intravenous vasopressor) | 493 (60.0) | 85 (40.7) | 408 (66.7) | 0.53** |
| Elevated lactate | 676 (82.3) | 148 (70.8) | 528 (86.3) | 0.38* |
| Days from blood culture collection to the first acute organ dysfunction, median (IQR) | 1 (1 – 1) | 1 (-1 – 1) | 1 (1 – 1) | 0.11 |
| ***Lab test results, median (IQR)*** | | | | |
| Highest serum lactate (in mmol/L) | 4.0 (2.3 – 7.8) | 3.7 (1.8 – 7.4) | 4.2 (2.5 – 8.0) | -0.13 |
| >2.0 mmol/L | 662 (80.6) | 145 (69.4) | 517 (84.5) | 0.36* |
| >1.5 mmol/L | 731 (89.0) | 167 (79.9) | 564 (92.2) | 0.36* |
| Highest serum creatinine (in μmol/L) | 146 (92 – 253) | 125 (83 – 225) | 156 (97 – 269) | -0.03 |
| Highest/lowest serum creatinine ratio | 2.1 (1.7 – 3.3) | 1.9 (1.7 – 2.6) | 2.2 (1.7 – 3.6) | -0.25* |
| >2.0 fold | 451 (54.9) | 93 (44.5) | 358 (58.5) | 0.28* |
| >1.5 fold | 702 (85.5) | 173 (82.8) | 529 (86.4) | 0.10 |
| Lowest estimated glomerular filtration rate (eGFR) | 40 (19 – 71) | 51 (23 – 79) | 37 (18 – 69) | 0.21* |
| Lowest/highest eGFR ratio | 0.5 (0.3 – 0.7) | 0.6 (0.4 – 0.7) | 0.5 (0.3 – 0.7) | 0.31* |
| <50% | 409 (49.8) | 84 (40.2) | 325 (53.1) | 0.26* |
| <75% | 670 (81.6) | 158 (75.6) | 512 (83.7) | 0.20 |
| Highest serum total bilirubin (in μmol/L) | 15 (9 – 31) | 12 (7 – 24) | 16 (10 – 39) | -0.20 |
| >34.2 μmol/L | 186 (22.7) | 26 (12.4) | 160 (26.1) | 0.35* |
| >25.7 μmol/L | 250 (30.5) | 43 (20.6) | 207 (33.8) | 0.30* |
| Highest/lowest serum total bilirubin ratio | 2.0 (1.3 – 3.5) | 1.6 (1.0 – 2.9) | 2.2 (1.3 – 3.7) | -0.24* |
| >2.0 fold | 376 (45.8) | 68 (32.5) | 308 (50.3) | 0.36* |
| >1.5 fold | 500 (60.9) | 97 (46.4) | 403 (65.8) | 0.39* |
| Lowest serum platelet count (in 10^9^/L) | 119 (72 – 171) | 133 (83 – 180) | 117 (67 – 168) | 0.16 |
| <100 x10^9^/L | 331 (40.3) | 70 (33.5) | 261 (42.6) | 0.19 |
| <150 x10^9^/L | 530 (64.6) | 122 (58.4) | 408 (66.7) | 0.17 |
| Lowest/highest serum platelet count ratio | 0.3 (0.2 – 0.5) | 0.3 (0.2 – 0.5) | 0.3 (0.2 – 0.5) | 0.10 |
| <50% | 629 (76.6) | 154 (73.7) | 475 (77.6) | 0.09 |
| <75% | 786 (95.7) | 196 (93.8) | 590 (96.4) | 0.12 |
| Lowest P/F ratio value | 138 (82 – 202) | 158 (102 – 228) | 132 (78 – 193) | 0.28* |

Thresholds for the absolute values of standardized differences: 0≤d≤0.2 negligible effect size; 0.2<d≤0.4 small effect size (*); 0.4<d≤0.8 medium effect size (**); d>0.8 large effect size (***). The highest (or lowest) lab test result was the maximum (or minimum) value during the entire hospitalization. The terms ‘highest/lowest ratio’ and ‘lowest/highest ratio’ refer to the ratios of the maximum to minimum lab results, and vice versa, during the entire hospitalization.

## **eFigure B2.** Subsampling stability assessment for the best-performing algorithm among the top two clustering methods, K-Means and Robust and Sparse K-means Clustering (RSKC), based on Table 2.


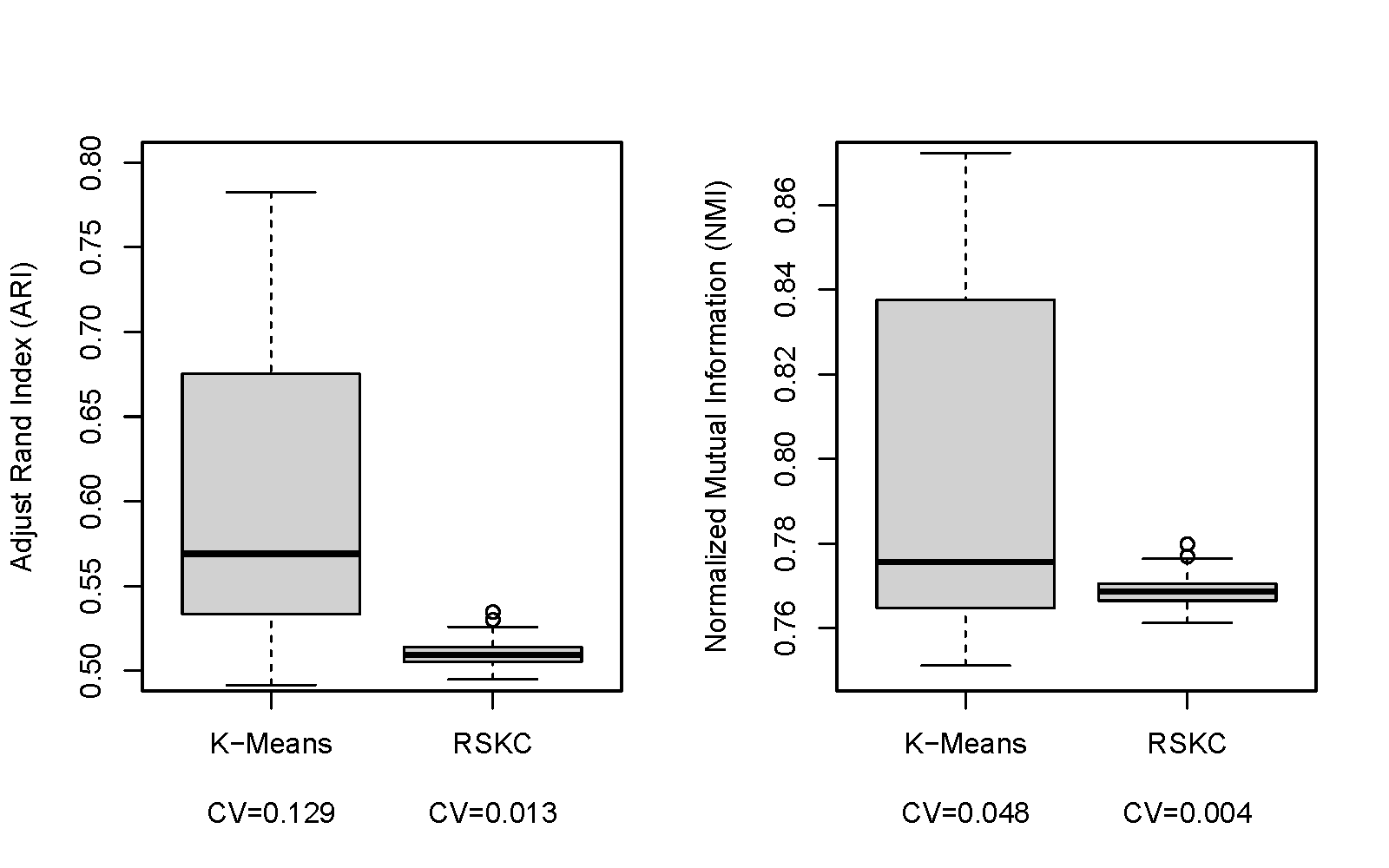


CV: Coefficient of variation.

## **eTable B4.** Feature importance using SHapley Additive exPlanations (SHAP)^23^ values derived from XGBoost^24^ for clusters identified by Robust and Sparse K-means Clustering (RSKC) in the development cohort.

| **Cluster ID** | **# of patients** | **% ASE(+)** | **% hospital mortality** | **Most common ICD-10 chapter*** | **Top Features (by SHAP)** |
| --- | --- | --- | --- | --- | --- |
| ***8*** | 176 | ***86.9*** | 42.6 | Diseases of the digestive system | Blood culture collected; SD of neutrophils values; SD of sodium values; SD of bilirubin values; Taken glycopeptide drugs; Max Urea value; Min CO2 content value; SD of FiO2 values |
| ***33*** | 111 | ***83.8*** | 23.4 | Diseases of the respiratory system | Total length of stay (days); SD of ALP values; SD of RDW values; Enteric culture collected; Taken glycopeptide drugs; Max neutrophil to lymphocyte ratio; Max chloride value; SD of eGFR values |
| ***4*** | 40 | ***82.5*** | 15.0 | Diseases of the respiratory system | Hospital-acquired infections; Taken penicillin drugs; SD of P/F ratio values; Min potassium value; Certain infectious and parasitic diseases; Diseases of the circulatory system; SD of eGFR values; Max eGFR value |
| ***5*** | 126 | ***78.6*** | 58.7 | Certain infectious and parasitic diseases | Min eGFR value; Max Urea value; SD of sodium values; SD of bilirubin values; Max creatinine value; Diseases of the genitourinary system; Hospital-acquired infections; SD of potassium values |
| ***42*** | 42 | ***73.8*** | 9.5 | Certain infectious and parasitic diseases | Certain infectious and parasitic diseases; Min potassium value; Diseases of the circulatory system; SD of ALP values; Min CO2 content value; Hospital-acquired infections; Hypertension Uncomplicated (chronic); SD of neutrophils values |
| ***9*** | 55 | ***72.7*** | 52.7 | Diseases of the circulatory system | SD of blood lactate values; Min eGFR value; SD of PCO2 arterial values; Max glucose value; Max chloride value; SD of eGFR values; SD of P/F ratio values; Min CO2 content value |
| ***26*** | 61 | ***65.6*** | 23.0 | Certain infectious and parasitic diseases | Max creatinine value; Taken glycopeptide drugs; Taken penicillin drugs; Max Urea value; Min creatinine value; Min CO2 content value; Diseases of the genitourinary system; Blood culture collected |
| ***35*** | 65 | ***63.1*** | 15.4 | Diseases of the circulatory system | SD of RDW values; Total length of stay (days); Respiratory culture collected; SD of MCV values; Max neutrophil to lymphocyte ratio; Max platelet counts; Taken miscellaneous drugs; Urine culture collected |
| ***48*** | 50 | ***60.0*** | 10.0 | Diseases of the respiratory system | Total length of stay (days); Taken penicillin drugs; SD of FiO2 values; Max CO2 content value; Min creatinine value; SD of RDW values; Max platelet counts; Blood culture collected |
| ***45*** | 42 | ***54.8*** | 11.9 | Injury, poisoning and certain other consequences of external causes | Max eGFR value; Total length of stay (days); SD of P/F ratio values; Min Urea value; Taken glycopeptide drugs; Max CO2 content value; Age at admission; Min potassium value |
| ***12*** | 53 | ***54.7*** | 13.2 | Diseases of the respiratory system | Respiratory culture collected; SD of PCO2 arterial values; SD of FiO2 values; SD of P/F ratio values; Max platelet counts; Min neutrophil to lymphocyte ratio ; Min Urea value; Max CO2 content value |
| 10 | 56 | 46.4 | 16.1 | Diseases of the circulatory system | SD of blood lactate values; SD of RDW values; Max RDW value; SD of eGFR values; Total length of stay (days); SD of sodium values; Min potassium value; SD of P/F ratio values |
| 36 | 40 | 42.5 | 5.0 | Diseases of the respiratory system | Max eGFR value; Max platelet counts; Min Urea value; Diseases of the circulatory system; Min eGFR value; Max Urea value; Age at admission; Blood culture collected |
| 22 | 52 | 38.5 | 34.6 | Injury, poisoning and certain other consequences of external causes | Max chloride value; SD of blood lactate values; Diseases of the circulatory system; Age at admission; SD of MCV values; SD of hemoglobin values; SD of FiO2 values; SD of PCO2 arterial values |
| 40 | 61 | 32.8 | 4.9 | Injury, poisoning and certain other consequences of external causes | Min Urea value; Min eGFR value; Age at admission; Diseases of the circulatory system; Max eGFR value; Injury, poisoning and certain other consequences of external causes; Taken cephalosporins drugs; Max creatinine value |
| 18 | 88 | 27.3 | 29.5 | Diseases of the circulatory system | Max RDW value; Min platelet counts; Min Urea value; SD of potassium values; SD of sodium values; Min eGFR value; Max eGFR value; Max Urea value |
| 20 | 50 | 26.0 | 10.0 | Diseases of the respiratory system | Max CO2 content value; Diseases of the respiratory system; Min MCHC value; Respiratory culture collected; SD of hemoglobin values; Max neutrophil to lymphocyte ratio; Max chloride value; Min platelet counts |
| 44 | 55 | 21.8 | 7.3 | Diseases of the circulatory system | Total length of stay (days); Max neutrophil to lymphocyte ratio; Diseases of the genitourinary system; Max eGFR value; Taken miscellaneous drugs; SD of sodium values; Max chloride value; Max RDW value |
| 38 | 29 | 20.7 | 24.1 | Diseases of the circulatory system | Max creatinine value; Min creatinine value; Min Urea value; Max eGFR value; Min eGFR value; Min RDW value; SD of eGFR values; Min potassium value |
| 6 | 45 | 15.6 | 15.6 | Diseases of the circulatory system | Min Urea value; Max creatinine value; Min eGFR value; Max eGFR value; Max Urea value; Total length of stay (days); Min neutrophil to lymphocyte ratio ; Min creatinine value |
| 19 | 37 | 10.8 | 45.9 | Diseases of the circulatory system | SD of blood lactate values; SD of FiO2 values; SD of P/F ratio values; SD of PCO2 arterial values; Max chloride value; Min potassium value; Max glucose value; Max platelet counts |
| 32 | 65 | 10.8 | 3.1 | Injury, poisoning and certain other consequences of external causes | Age at admission; Diseases of the circulatory system; Max Urea value; Max eGFR value; Injury, poisoning and certain other consequences of external causes; Min eGFR value; Max glucose value; Severe events proximally threatening to life |
| 23 | 74 | 8.1 | 5.4 | Diseases of the circulatory system | Max eGFR value; Min eGFR value; SD of neutrophils values; Heart attack (AMI); Taken antimicrobials; SD of blood lactate values; Diseases of the respiratory system; Diseases of the circulatory system |
| 2 | 59 | 6.8 | 5.1 | Diseases of the circulatory system | Congestive Heart Failure (chronic); Max eGFR value; Age at admission; Min eGFR value; Cardiac Arrhythmia (chronic); Total length of stay (days); SD of PCO2 arterial values; Min MCHC value |
| 28 | 38 | 5.3 | 0 | Diseases of the respiratory system | Diseases of the circulatory system; Max eGFR value; Min eGFR value; Age at admission; Min hematocrit value; Taken antimicrobials; Severe events proximally threatening to life; Respiratory culture collected |
| 41 | 56 | 3.6 | 3.6 | Diseases of the circulatory system | SD of PCO2 arterial values; Min hematocrit value; SD of RDW values; SD of hemoglobin values; SD of FiO2 values; Taken cephalosporins drugs; Total length of stay (days); Max platelet counts |
| 14 | 31 | 3.2 | 19.4 | Diseases of the circulatory system | Min Urea value; Min eGFR value; Min creatinine value; Max eGFR value; Max glucose value; Max creatinine value; Min RDW value; Age at admission |
| 11 | 58 | 1.7 | 0 | Diseases of the circulatory system | Taken antimicrobials; SD of RDW values; Min creatinine value; Max glucose value; SD of FiO2 values; Taken cephalosporins drugs; Severe events proximally threatening to life; SD of blood lactate values |
| 34 | 65 | 1.5 | 4.6 | Diseases of the circulatory system | Min eGFR value; Max eGFR value; Max Urea value; Min Urea value; Taken antimicrobials; SD of hemoglobin values; Received invasive mechanical ventilation; Min creatinine value |
| 43 | 82 | 1.2 | 1.2 | Diseases of the circulatory system | SD of blood lactate values; Max eGFR value; Urine culture collected; SD of potassium values; Min Urea value; Age at admission; Hypertension Uncomplicated (chronic); Min hematocrit value |
| 27 | 95 | 1.1 | 1.1 | Diseases of the circulatory system | Min hematocrit value; SD of blood lactate values; Received invasive mechanical ventilation; SD of hemoglobin values; SD of potassium values; SD of FiO2 values; Min platelet counts; Total length of stay (days) |
| 1 | 67 | 0 | 4.5 | Diseases of the circulatory system | SD of blood lactate values; Max eGFR value; SD of potassium values; Min platelet counts; SD of PCO2 arterial values; Taken cephalosporins drugs; Min creatinine value; Congestive Heart Failure (chronic) |
| 3 | 68 | 0 | 10.3 | Diseases of the circulatory system | Max CO2 content value; Taken antimicrobials; Taken cephalosporins drugs; SD of FiO2 values; SD of sodium values; Min eGFR value; Max chloride value; Min neutrophil to lymphocyte ratio |
| 7 | 85 | 0 | 0 | Diseases of the circulatory system | SD of PCO2 arterial values; Min hematocrit value; SD of potassium values; SD of blood lactate values; Min eGFR value; Min CO2 content value; Max eGFR value; SD of hemoglobin values |
| 13 | 92 | 0 | 1.1 | Diseases of the circulatory system | Max eGFR value; SD of neutrophils values; Min hematocrit value; SD of sodium values; Taken antimicrobials; Min Urea value; SD of potassium values; Min potassium value |
| 15 | 30 | 0 | 66.7 | Diseases of the circulatory system | SD of blood lactate values; Max glucose value; Max CO2 content value; Min CO2 content value; Max eGFR value; Taken antimicrobials; Max chloride value; SD of PCO2 arterial values |
| 16 | 70 | 0 | 0 | Diseases of the circulatory system | Cardiac Arrhythmia (chronic); Age at admission; Min CO2 content value; Congestive Heart Failure (chronic); SD of hemoglobin values; Max eGFR value; SD of neutrophils values; Min eGFR value |
| 17 | 99 | 0 | 3.0 | Diseases of the circulatory system | SD of blood lactate values; Age at admission; SD of MCV values; SD of P/F ratio values; Cardiac Arrhythmia (chronic); Max neutrophil to lymphocyte ratio; Min CO2 content value; Max eGFR value |
| 21 | 84 | 0 | 0 | Diseases of the circulatory system | Min hematocrit value; SD of sodium values; Heart attack (AMI); SD of MCHC values; Min MCHC value; Min eGFR value; Taken antimicrobials; SD of potassium values |
| 24 | 125 | 0 | 2.4 | Diseases of the circulatory system | SD of PCO2 arterial values; Max eGFR value; Taken antimicrobials; Min hematocrit value; Min eGFR value; Min Urea value; Heart attack (AMI); Urine culture collected |
| 25 | 243 | 0 | 0.8 | Diseases of the circulatory system | Min hematocrit value; SD of blood lactate values; Min eGFR value; Max neutrophil to lymphocyte ratio; Urine culture collected; Max chloride value; SD of hemoglobin values; Total length of stay (days) |
| 29 | 222 | 0 | 0.5 | Diseases of the circulatory system | Min eGFR value; Min hematocrit value; Max eGFR value; Min MCHC value; SD of potassium values; Taken antimicrobials; Max neutrophil to lymphocyte ratio; SD of sodium values |
| 30 | 51 | 0 | 2.0 | Injury, poisoning and certain other consequences of external causes | Min eGFR value; Max eGFR value; Age at admission; Diseases of the circulatory system; Max Urea value; Injury, poisoning and certain other consequences of external causes; SD of blood lactate values; Min Urea value |
| 31 | 47 | 0 | 2.1 | Diseases of the circulatory system | Min Urea value; Max eGFR value; Min creatinine value; Min potassium value; Age at admission; Taken antimicrobials; Max creatinine value; SD of RDW values |
| 37 | 173 | 0 | 0.6 | Diseases of the circulatory system | Taken antimicrobials; SD of sodium values; SD of potassium values; Min hematocrit value; Max neutrophil to lymphocyte ratio; Total length of stay (days); SD of hemoglobin values; Heart attack (AMI) |
| 39 | 6 | 0 | 0 | Diseases of the circulatory system | Max eGFR value; Min eGFR value; End-stage Renal Disease; SD of sodium values; Max platelet counts; Min creatinine value; Max neutrophil to lymphocyte ratio; SD of MCHC values |
| 46 | 67 | 0 | 0 | Diseases of the circulatory system | Age at admission; Diseases of the circulatory system; Max Urea value; Max eGFR value; SD of PCO2 arterial values; Min eGFR value; SD of sodium values; Min platelet counts |
| 47 | 174 | 0 | 0.6 | Diseases of the circulatory system | Taken cephalosporins drugs; Min hematocrit value; Min eGFR value; Max eGFR value; SD of FiO2 values; SD of PCO2 arterial values; Taken antimicrobials; SD of blood lactate values |

ASE: Adult Sepsis Event definition of sepsis; SD: standard deviation.

*The most common ICD-10 chapter was defined by the most responsible diagnosis^25^ according to the userguide of ICD-10-CA^26^.

## **eTable B5.** Most common categories of Most Responsible Hospital Diagnoses (MRHD), by cluster.

| **ICD-10 Chapter of Most Responsible Hospital Diagnosis** | **ASE-majority Clusters** | | | **ASE-minority Clusters** | | |
| --- | --- | --- | --- | --- | --- | --- |
|  | # clusters  (% of 11 clusters) | # patients in the clusters  (% of 821 patients) | # ASE cases in the clusters  (% of # patients in the cluster) | # clusters  (% of 37 clusters) | # patients in the clusters  (% of 2,839 patients) | # ASE cases in the clusters  (% of # patients in the cluster) |
| Diseases of the circulatory system | 2 (18.2) | 120 (14.6) | 81 (67.5) | 30 (81.1) | 2,482 (87.4) | 96 (3.9) |
| Injury, poisoning and certain other consequences of external causes | 1 (9.1) | 42 (5.1) | 23 (54.8) | 4 (10.8) | 229 (8.1) | 47 (20.5) |
| Diseases of the respiratory system | 4 (36.3) | 254 (30.9) | 185 (72.8) | 3 (8.1) | 128 (4.5) | 32 (25.0) |
| Certain infectious and parasitic diseases | 3 (27.3) | 229 (27.9) | 170 (74.2) |  |  |  |
| Diseases of the digestive system | 1 (9.1) | 176 (21.4) | 153 (86.9) |  |  |  |

Regarding the most common category of Most Responsible Hospital Diagnosis (MRHD), there was more variation within the ASE-majority versus ASE-minority clusters, as manifested in three ways. First (1^st^ and 4^th^ data columns of eTable B5), just three diagnostic categories contained the most common MRHD for all 37 ASE-minority clusters, with diseases of the circulatory system being that category for 81% of 37 clusters. This is consistent with the known dominance of cardiovascular disorders as reasons for admission to hospitals and ICUs.^27,28^ On the other hand, among the 11 ASE-majority clusters, five different categories were represented (respiratory; infectious; circulatory; digestive; external causes including injury and poisonings), with none representing more than 37% of those 11 clusters. Second (2^nd^ and 5^th^ data columns of eTable B5), within the most common categories of MRHD, patients were less concentrated for ASE-majority versus ASE-minority clusters. Third (3^rd^ and 6^th^ data columns of eTable B5), ASE cases were more concentrated in ASE-majority clusters than in ASE-minority clusters within the most common categories of MRHD.

## **eFigure B3.** Feature importance heatmap using SHapley Additive exPlanations (SHAP) values derived from XGBoost for clusters identified by Robust and Sparse K-means Clustering (RSKC) for the development cohort, in descending left-to-right order of fraction meeting Adult Sepsis Event definition.


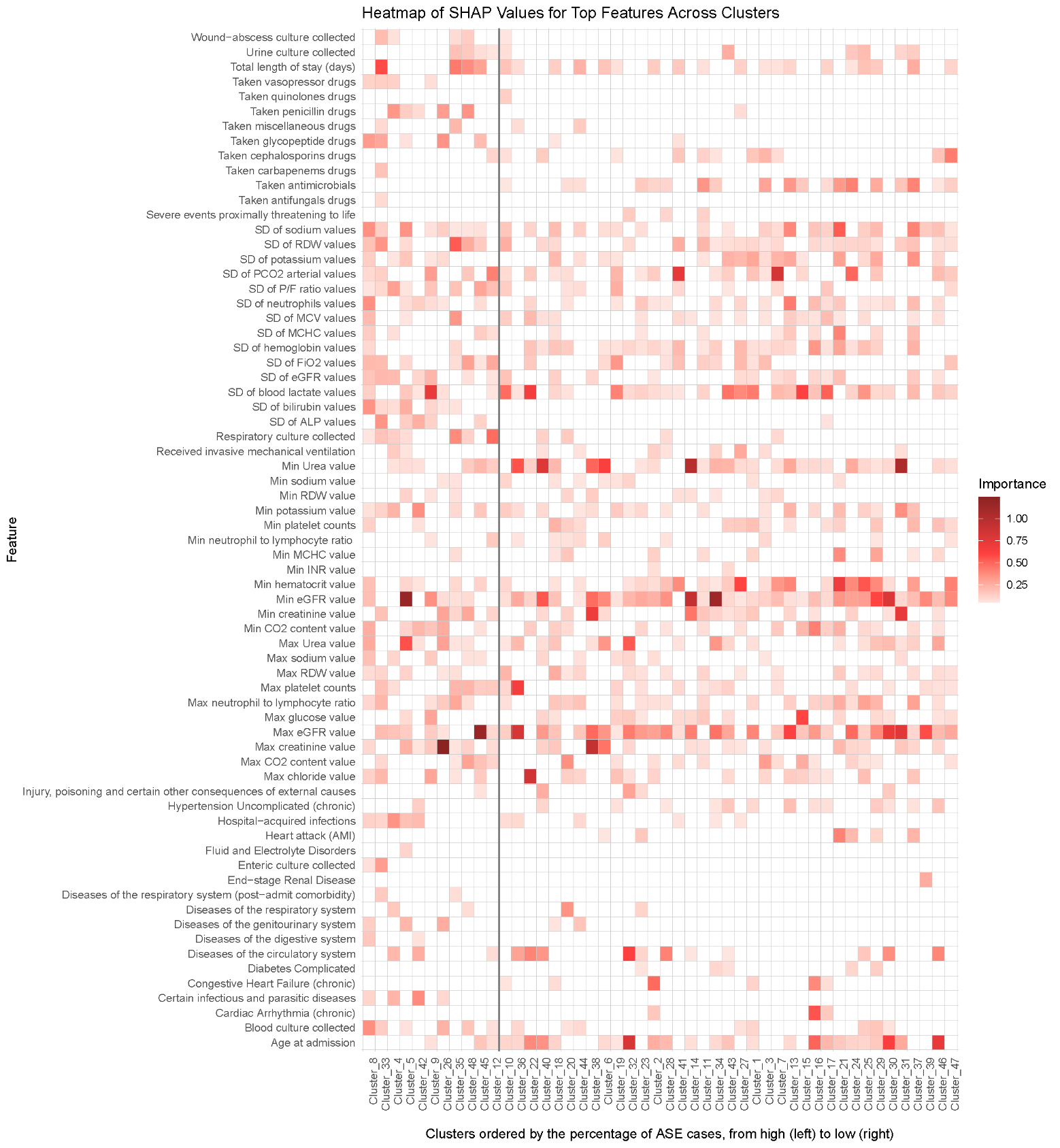


## **eTable B6.** Patient characteristics between the ASE-majority clusters and the ASE-minority clusters in the development cohort. Values are n (%) unless otherwise indicated.

| **Characteristic**  N (%) | **ASE-minority Clusters**  N = 2,839 (77.6) | **ASE-majority Clusters**  N = 821 (22.4) | **p-value** |
| --- | --- | --- | --- |
| Age at admission, median (IQR) | 63 (52 – 73) | 61 (51 – 72) | 0.017 |
| Blood culture collected | 569 (20.0) | 779 (94.9) | <0.001 |
| Respiratory culture collected | 272 (9.6) | 550 (67.0) | <0.001 |
| Urine culture collected | 644 (22.7) | 725 (88.3) | <0.001 |
| Wound-abscess culture collected | 177 (6.2) | 424 (51.6) | <0.001 |
| Cardiac arrhythmia (chronic) | 515 (18.1) | 150 (18.3) | 0.932 |
| Certain infectious and parasitic diseases | 51 (1.8) | 294 (35.8) | <0.001 |
| Congestive heart failure (chronic) | 368 (13.0) | 102 (12.4) | 0.685 |
| Diabetes complicated | 621 (21.9) | 181 (22.0) | 0.916 |
| Diseases of the circulatory system | 2,298 (80.9) | 389 (47.4) | <0.001 |
| Diseases of the digestive system | 136 (4.8) | 261 (31.8) | <0.001 |
| Diseases of the genitourinary system | 164 (5.8) | 300 (36.5) | <0.001 |
| Diseases of the respiratory system (post-admit comorbidity) | 158 (5.6) | 286 (34.8) | <0.001 |
| Diseases of the respiratory system | 302 (10.6) | 358 (43.6) | <0.001 |
| End-stage renal disease | 23 (0.8) | 23 (2.8) | <0.001 |
| Enteric culture collected | 118 (4.2) | 311 (37.9) | <0.001 |
| Fluid and electrolyte disorders | 155 (5.5) | 240 (29.2) | <0.001 |
| Heart attack (AMI) | 1,107 (39.0) | 70 (8.5) | <0.001 |
| Hospital-acquired infections | 189 (6.7) | 532 (64.8) | <0.001 |
| Hypertension uncomplicated (chronic) | 1,401 (49.3) | 314 (38.2) | <0.001 |
| Injury, poisoning and certain other consequences of external causes | 271 (9.5) | 169 (20.6) | <0.001 |
| Maximum chloride value, median (IQR) | 108 (106 – 111) | 114 (110 – 119) | <0.001 |
| Maximum CO2 content value, median (IQR) | 27 (25 – 29) | 29 (26 – 32) | <0.001 |
| Maximum creatinine value, median (IQR) | 91 (75 – 112) | 146 (92 – 253) | <0.001 |
| Maximum eGFR value, median (IQR) | 91 (77 – 104) | 99 (69 – 115) | <0.001 |
| Maximum glucose value, median (IQR) | 9.2 (6.9 – 11.1) | 12.7 (9.9 – 17.0) | <0.001 |
| Maximum neutrophil to lymphocyte ratio, median (IQR) | 8 (4 – 14) | 24 (15 – 44) | <0.001 |
| Maximum platelet counts, median (IQR) | 248 (201 – 304) | 381 (251 – 540) | <0.001 |
| Maximum RDW value, median (IQR) | 14.1 (13.4 – 15.1) | 16.7 (15.1 – 19.3) | <0.001 |
| Maximum sodium value, median (IQR) | 141 (139 – 142) | 145 (142 – 150) | <0.001 |
| Maximum Urea value, median (IQR) | 7 (5 – 10) | 13 (8 – 21) | <0.001 |
| Minimum CO2 content value, median (IQR) | 22 (20 – 23) | 17 (14 – 20) | <0.001 |
| Minimum creatinine value, median (IQR) | 71 (58 – 84) | 59 (44 – 89) | <0.001 |
| Minimum eGFR value, median (IQR) | 73 (55 – 90) | 40 (19 – 71) | <0.001 |
| Minimum hematocrit value, median (IQR) | 0.36 (0.30 – 0.41) | 0.25 (0.21 – 0.29) | <0.001 |
| Minimum INR value, median (IQR) | 1.1 (1.0 – 1.1) | 1.1 (1.0 – 1.2) | 0.012 |
| Minimum MCHC value, median (IQR) | 327 (321 – 332) | 319 (312 – 325) | <0.001 |
| Minimum neutrophil to lymphocyte ratio , median (IQR) | 2.5 (1.7 – 3.5) | 2.8 (1.6 – 4.8) | <0.001 |
| Minimum platelet counts, median (IQR) | 176 (137 – 219) | 119 (72 – 171) | <0.001 |
| Minimum potassium value, median (IQR) | 3.7 (3.4 – 3.9) | 3.1 (2.8 – 3.3) | <0.001 |
| Minimum RDW value, median (IQR) | 13.5 (12.9 – 14.3) | 14.2 (13.3 – 15.5) | <0.001 |
| Minimum sodium value, median (IQR) | 135 (133 – 138) | 132 (128 – 135) | <0.001 |
| Minimum Urea value, median (IQR) | 6.1 (4.3 – 6.5) | 4.7 (3.2 – 7.2) | <0.001 |
| Received invasive mechanical ventilation | 850 (29.9) | 577 (70.3) | <0.001 |
| SD of ALP values, median (IQR) | 0 (0 – 0) | 18 (2 – 38) | <0.001 |
| SD of total bilirubin values, median (IQR) | 0 (0 – 0) | 3.0 (0.7 – 8.5) | <0.001 |
| SD of serum lactate values, median (IQR) | 0.0 (0.0 – 0.4) | 0.9 (0.5 – 1.9) | <0.001 |
| SD of eGFR values, median (IQR) | 6 (3 – 9) | 12 (7 – 19) | <0.001 |
| SD of FiO2 values, median (IQR) | 0 (0 – 0) | 13 (7 – 21) | <0.001 |
| SD of hemoglobin values, median (IQR) | 7.3 (4.9 – 10.9) | 12.0 (9.3 – 16.1) | <0.001 |
| SD of MCHC values, median (IQR) | 4.0 (2.8 – 5.4) | 5.8 (4.5 – 7.3) | <0.001 |
| SD of MCV values, median (IQR) | 0.7 (0.5 – 1.0) | 1.3 (1.0 – 2.1) | <0.001 |
| SD of neutrophils values, median (IQR) | 1.7 (0.9 – 2.7) | 3.4 (2.5 – 5.0) | <0.001 |
| SD of P/F ratio values, median (IQR) | 0 (0 – 0) | 45 (21 – 63) | <0.001 |
| SD of PCO2 arterial values, median (IQR) | 0.0 (0.0 – 3.2) | 5.6 (3.7 – 8.0) | <0.001 |
| SD of potassium values, median (IQR) | 0.3 (0.2 – 0.5) | 0.5 (0.4 – 0.6) | <0.001 |
| SD of RDW values, median (IQR) | 0.2 (0.1 – 0.3) | 0.6 (0.4 – 1.0) | <0.001 |
| SD of sodium values, median (IQR) | 1.9 (1.3 – 2.6) | 3.6 (2.6 – 4.8) | <0.001 |
| Severe events proximally threatening to life | 1,302 (45.9) | 436 (53.1) | <0.001 |
| Administered antifungals drugs | 37 (1.3) | 241 (29.4) | <0.001 |
| Administered antimicrobials | 1,349 (47.5) | 814 (99.1) | <0.001 |
| Administered carbapenems drugs | 25 (0.9) | 185 (22.5) | <0.001 |
| Administered cephalosporins drugs | 1,006 (35.4) | 582 (70.9) | <0.001 |
| Administered glycopeptide drugs | 129 (4.5) | 474 (57.7) | <0.001 |
| Administered miscellaneous drugs | 188 (6.6) | 388 (47.3) | <0.001 |
| Administered penicillin drugs | 240 (8.5) | 596 (72.6) | <0.001 |
| Administered quinolones drugs | 206 (7.3) | 291 (35.4) | <0.001 |
| Administered vasopressor drugs | 191 (6.7) | 493 (60.0) | <0.001 |
| Total length of stay (days), median (IQR) | 6 (4 – 11) | 21 (11 – 40) | <0.001 |
| Meet Adult Sepsis Event (ASE) criteria | 175 (6.2) | 612 (74.5) | <0.001 |

P-values were calculated from Wilcoxon rank sum test, Pearson’s Chi-squared test, or Fisher’s exact test, not adjusted for multiple comparisons; IQR: Interquartile range.

SD: Standard deviation, calculated by two or more measures during the entire hospitalization per patient. For lab tests with only one result value, the SD was set to 0; ALP: Alkaline phosphatase; ALT: Alanine transaminase; eGFR: Estimated glomerular filtration rate; INR: International normalized ratio; MCHC: Mean corpuscular hemoglobin concentration; MCV: Mean corpuscular volume; PTT: Partial thromboplastin time; RDW: Red cell distribution width; WBC: White blood count.

## **eTable B7.** Patient characteristics for the 11 ASE-majority clusters in the development cohort. Patients identified by Robust and Sparse K-means Clustering (RSKC). Values are n (%) unless indicated.

| Characteristic | **Cluster 8** | **Cluster 33** | **Cluster 4** | **Cluster 5** | **Cluster 42** | **Cluster 9** | **Cluster 26** | **Cluster 35** | **Cluster 48** | **Cluster 45** | **Cluster 12** |
| --- | --- | --- | --- | --- | --- | --- | --- | --- | --- | --- | --- |
| N (%) | N = 176 | N = 111 | N = 40 | N = 126 | N = 42 | N = 55 | N = 61 | N = 65 | N = 50 | N = 42 | N = 53 |
| Age, median (interquartile range [IQR]) | 57 (50 – 69) | 59 (48 – 67) | 55 (48 – 65) | 67 (59 – 78) | 57 (43 – 70) | **70 (60 – 79)** | 67 (57 – 76) | 66 (55 – 76) | 63 (57 – 71) | **36 (26 – 45)** | 61 (54 – 64) |
| Female | 77 (43.8) | 53 (47.7) | 17 (42.5) | 60 (47.6) | 23 (54.8) | 22 (40.0) | **17 (27.9)** | 28 (43.1) | 21 (42.0) | 12 (28.6) | **21 (39.6)** |
| ***Chronic comorbid conditions^1^*** | | | | | | | | | | | |
| Number of comorbid conditions, median (IQR) | 3 (2 – 4) | 2 (1 – 3) | 2 (1 – 3) | **4 (3 – 5)** | 2 (1 – 2) | 2 (2 – 4) | 3 (2 – 4) | 3 (2 – 4) | 2 (1 – 3) | 2 (1 – 3) | 2 (1 – 3) |
| Congestive Heart Failure | 22 (12.5) | 12 (10.8) | 2 (5.0) | 37 (29.4) | 1 (2.4) | 12 (21.8) | 17 (27.9) | 11 (16.9) | 5 (10.0) | 2 (4.8) | 4 (7.5) |
| Cardiac Arrhythmia | 51 (29.0) | 33 (29.7) | 4 (10.0) | 52 (41.3) | 6 (14.3) | 13 (23.6) | 19 (31.1) | 23 (35.4) | 8 (16.0) | 5 (11.9) | 12 (22.6) |
| Valvular Disease | 7 (4.0) | 4 (3.6) | 3 (7.5) | 16 (12.7) | 2 (4.8) | 3 (5.5) | 5 (8.2) | 5 (7.7) | 3 (6.0) | 1 (2.4) | 2 (3.8) |
| Pulmonary Circulation Disorders | 7 (4.0) | 10 (9.0) | 4 (10.0) | 10 (7.9) | 4 (9.5) | 4 (7.3) | 3 (4.9) | 5 (7.7) | 2 (4.0) | 3 (7.1) | 3 (5.7) |
| Peripheral Vascular Disorders | 16 (9.1) | 5 (4.5) | 2 (5.0) | 12 (9.5) | 1 (2.4) | 5 (9.1) | 5 (8.2) | 5 (7.7) | 1 (2.0) | 0 (0.0) | 4 (7.5) |
| Hypertension Uncomplicated | 58 (33.0) | 36 (32.4) | 13 (32.5) | 62 (49.2) | 6 (14.3) | 32 (58.2) | 33 (54.1) | 36 (55.4) | 20 (40.0) | 4 (9.5) | 28 (52.8) |
| Hypertension Complicated | 1 (0.6) | 0 (0.0) | 0 (0.0) | 2 (1.6) | 0 (0.0) | 0 (0.0) | 0 (0.0) | 0 (0.0) | 0 (0.0) | 0 (0.0) | 0 (0.0) |
| Paralysis | 3 (1.7) | 6 (5.4) | 0 (0.0) | 1 (0.8) | 1 (2.4) | 2 (3.6) | 0 (0.0) | 9 (13.8) | 3 (6.0) | 2 (4.8) | 1 (1.9) |
| Other Neurological Disorders | 24 (13.6) | 15 (13.5) | 5 (12.5) | 17 (13.5) | 3 (7.1) | 11 (20.0) | 4 (6.6) | 9 (13.8) | 8 (16.0) | 9 (21.4) | 5 (9.4) |
| Chronic Pulmonary Disease | 10 (5.7) | 23 (20.7) | 7 (17.5) | 14 (11.1) | 6 (14.3) | 5 (9.1) | 6 (9.8) | 9 (13.8) | 17 (34.0) | 3 (7.1) | 17 (32.1) |
| Diabetes Uncomplicated | 33 (18.8) | 7 (6.3) | 5 (12.5) | 17 (13.5) | 5 (11.9) | 6 (10.9) | 11 (18.0) | 8 (12.3) | 5 (10.0) | 5 (11.9) | 3 (5.7) |
| Diabetes Complicated | 43 (24.4) | 10 (9.0) | 2 (5.0) | 54 (42.9) | 5 (11.9) | 13 (23.6) | 30 (49.2) | 13 (20.0) | 4 (8.0) | 0 (0.0) | 7 (13.2) |
| Hypothyroidism | 5 (2.8) | 1 (0.9) | 1 (2.5) | 1 (0.8) | 0 (0.0) | 1 (1.8) | 2 (3.3) | 3 (4.6) | 0 (0.0) | 0 (0.0) | 1 (1.9) |
| Renal Failure | 13 (7.4) | 0 (0.0) | 0 (0.0) | 20 (15.9) | 0 (0.0) | 5 (9.1) | 7 (11.5) | 0 (0.0) | 0 (0.0) | 0 (0.0) | 0 (0.0) |
| Liver Disease | 57 (32.4) | 8 (7.2) | 2 (5.0) | 27 (21.4) | 2 (4.8) | 3 (5.5) | 4 (6.6) | 6 (9.2) | 0 (0.0) | 2 (4.8) | 1 (1.9) |
| Peptic ulcer disease without bleeding | 3 (1.7) | 0 (0.0) | 0 (0.0) | 1 (0.8) | 0 (0.0) | 0 (0.0) | 1 (1.6) | 0 (0.0) | 1 (2.0) | 0 (0.0) | 0 (0.0) |
| AIDS/HIV | 0 (0.0) | 0 (0.0) | 0 (0.0) | 0 (0.0) | 0 (0.0) | 0 (0.0) | 0 (0.0) | 0 (0.0) | 0 (0.0) | 1 (2.4) | 0 (0.0) |
| Lymphoma | 4 (2.3) | 2 (1.8) | 1 (2.5) | 5 (4.0) | 2 (4.8) | 0 (0.0) | 0 (0.0) | 0 (0.0) | 0 (0.0) | 1 (2.4) | 0 (0.0) |
| Metastatic Cancer | 3 (1.7) | 3 (2.7) | 1 (2.5) | 4 (3.2) | 1 (2.4) | 0 (0.0) | 0 (0.0) | 2 (3.1) | 3 (6.0) | 1 (2.4) | 3 (5.7) |
| Solid Tumor without Metastasis | 9 (5.1) | 7 (6.3) | 1 (2.5) | 8 (6.3) | 2 (4.8) | 6 (10.9) | 2 (3.3) | 4 (6.2) | 5 (10.0) | 1 (2.4) | 5 (9.4) |
| Rheumatoid Arthritis/collagen | 6 (3.4) | 6 (5.4) | 0 (0.0) | 3 (2.4) | 1 (2.4) | 1 (1.8) | 2 (3.3) | 5 (7.7) | 0 (0.0) | 0 (0.0) | 1 (1.9) |
| Coagulopathy | 36 (20.5) | 9 (8.1) | 3 (7.5) | 17 (13.5) | 6 (14.3) | 6 (10.9) | 7 (11.5) | 4 (6.2) | 0 (0.0) | 2 (4.8) | 1 (1.9) |
| Obesity | 5 (2.8) | 2 (1.8) | 3 (7.5) | 6 (4.8) | 2 (4.8) | 1 (1.8) | 2 (3.3) | 3 (4.6) | 3 (6.0) | 1 (2.4) | 6 (11.3) |
| Weight Loss | 5 (2.8) | 11 (9.9) | 1 (2.5) | 3 (2.4) | 1 (2.4) | 0 (0.0) | 0 (0.0) | 3 (4.6) | 2 (4.0) | 0 (0.0) | 0 (0.0) |
| Fluid and Electrolyte Disorders | 72 (40.9) | 28 (25.2) | 7 (17.5) | 55 (43.7) | 7 (16.7) | 15 (27.3) | 12 (19.7) | 20 (30.8) | 6 (12.0) | 9 (21.4) | 9 (17.0) |
| Blood Loss Anemia | 3 (1.7) | 1 (0.9) | 0 (0.0) | 0 (0.0) | 0 (0.0) | 1 (1.8) | 0 (0.0) | 1 (1.5) | 0 (0.0) | 0 (0.0) | 0 (0.0) |
| Deficiency Anemia | 4 (2.3) | 4 (3.6) | 0 (0.0) | 5 (4.0) | 1 (2.4) | 0 (0.0) | 1 (1.6) | 3 (4.6) | 0 (0.0) | 0 (0.0) | 1 (1.9) |
| Alcohol Abuse | 39 (22.2) | 15 (13.5) | 5 (12.5) | 10 (7.9) | 3 (7.1) | 2 (3.6) | 5 (8.2) | 3 (4.6) | 4 (8.0) | 10 (23.8) | 14 (26.4) |
| Drug Abuse | 8 (4.5) | 10 (9.0) | 3 (7.5) | 2 (1.6) | 3 (7.1) | 2 (3.6) | 2 (3.3) | 1 (1.5) | 1 (2.0) | 4 (9.5) | 2 (3.8) |
| Psychoses | 1 (0.6) | 1 (0.9) | 1 (2.5) | 0 (0.0) | 1 (2.4) | 0 (0.0) | 0 (0.0) | 0 (0.0) | 2 (4.0) | 1 (2.4) | 0 (0.0) |
| Depression | 8 (4.5) | 8 (7.2) | 2 (5.0) | 1 (0.8) | 1 (2.4) | 0 (0.0) | 3 (4.9) | 3 (4.6) | 3 (6.0) | 5 (11.9) | 2 (3.8) |
| End-stage Renal Disease | 1 (0.6) | 0 (0.0) | 0 (0.0) | 16 (12.7) | 0 (0.0) | 0 (0.0) | 6 (9.8) | 0 (0.0) | 0 (0.0) | 0 (0.0) | 0 (0.0) |
| ***ICD-10 chapters by most responsible diagnosis^2^*** | | | | | | | | | | | |
| Certain infectious and parasitic diseases | **40 (22.7)** | 15 (13.5) | **12 (30.0)** | **30 (23.8)** | **15 (35.7)** | 2 (3.6) | **16 (26.2)** | 1 (1.5) | 1 (2.0) | 2 (4.8) | 0 (0.0) |
| Neoplasms | 10 (5.7) | 5 (4.5) | 0 (0.0) | 2 (1.6) | 2 (4.8) | 4 (7.3) | 0 (0.0) | 4 (6.2) | 5 (10.0) | 2 (4.8) | 4 (7.5) |
| Diseases of the blood and blood-forming organs and certain disorders | 2 (1.1) | 0 (0.0) | 0 (0.0) | 0 (0.0) | 0 (0.0) | 0 (0.0) | 0 (0.0) | 0 (0.0) | 0 (0.0) | 0 (0.0) | 0 (0.0) |
| Endocrine, nutritional and metabolic diseases | 10 (5.7) | 0 (0.0) | 0 (0.0) | 6 (4.8) | 0 (0.0) | 2 (3.6) | 5 (8.2) | 1 (1.5) | 1 (2.0) | 0 (0.0) | 2 (3.8) |
| Mental and behavioural disorders | 1 (0.6) | 4 (3.6) | 1 (2.5) | 4 (3.2) | 1 (2.4) | 0 (0.0) | 0 (0.0) | 0 (0.0) | 1 (2.0) | 2 (4.8) | 0 (0.0) |
| Diseases of the nervous system | 4 (2.3) | 2 (1.8) | 1 (2.5) | 4 (3.2) | 1 (2.4) | 6 (10.9) | 2 (3.3) | 2 (3.1) | 1 (2.0) | 4 (9.5) | 2 (3.8) |
| Diseases of the circulatory system | 12 (6.8) | 14 (12.6) | 1 (2.5) | **28 (22.2)** | 1 (2.4) | **22 (40.0)** | **12 (19.7)** | **25 (38.5)** | **13 (26.0)** | 1 (2.4) | **15 (28.3)** |
| Diseases of the respiratory system | 18 (10.2) | **26 (23.4)** | **14 (35.0)** | 17 (13.5) | 3 (7.1) | **9 (16.4)** | 6 (9.8) | **11 (16.9)** | **20 (40.0)** | 5 (11.9) | **19 (35.8)** |
| Diseases of the digestive system | **43 (24.4)** | 17 (15.3) | 6 (15.0) | 16 (12.7) | **9 (21.4)** | 6 (10.9) | 6 (9.8) | 5 (7.7) | 3 (6.0) | 2 (4.8) | 1 (1.9) |
| Diseases of the skin and subcutaneous tissue | 2 (1.1) | 1 (0.9) | 0 (0.0) | 1 (0.8) | 1 (2.4) | 0 (0.0) | 0 (0.0) | 0 (0.0) | 1 (2.0) | 0 (0.0) | 0 (0.0) |
| Diseases of the musculoskeletal system and connective tissue | 6 (3.4) | 3 (2.7) | 0 (0.0) | 1 (0.8) | 0 (0.0) | 0 (0.0) | 0 (0.0) | 1 (1.5) | 0 (0.0) | 0 (0.0) | 1 (1.9) |
| Diseases of the genitourinary system | 2 (1.1) | 1 (0.9) | 0 (0.0) | 4 (3.2) | 2 (4.8) | 1 (1.8) | 3 (4.9) | 1 (1.5) | 0 (0.0) | 0 (0.0) | 0 (0.0) |
| Pregnancy, childbirth and the puerperium | 2 (1.1) | 0 (0.0) | 1 (2.5) | 0 (0.0) | 0 (0.0) | 0 (0.0) | 0 (0.0) | 0 (0.0) | 0 (0.0) | 0 (0.0) | 0 (0.0) |
| Congenital malformations, deformations and chromosomal abnormalities | 0 (0.0) | 1 (0.9) | 0 (0.0) | 1 (0.8) | 0 (0.0) | 0 (0.0) | 0 (0.0) | 0 (0.0) | 0 (0.0) | 0 (0.0) | 1 (1.9) |
| Symptoms, signs and abnormal clinical and laboratory findings, not elsewhere classified | 5 (2.8) | 1 (0.9) | 0 (0.0) | 2 (1.6) | 2 (4.8) | 0 (0.0) | 5 (8.2) | 2 (3.1) | 0 (0.0) | 0 (0.0) | 1 (1.9) |
| Injury, poisoning and certain other consequences of external causes | 18 (10.2) | 16 (14.4) | 4 (10.0) | 10 (7.9) | 5 (11.9) | 3 (5.5) | 6 (9.8) | 9 (13.8) | 3 (6.0) | **19 (45.2)** | 5 (9.4) |
| Factors influencing health status and contact with health services | 1 (0.6) | 5 (4.5) | 0 (0.0) | 0 (0.0) | 0 (0.0) | 0 (0.0) | 0 (0.0) | 3 (4.6) | 1 (2.0) | 5 (11.9) | 2 (3.8) |
| ***Clinical characteristics during hospitalization*** | | | | | | | | | | | |
| Admitted via Emergency Department | 152 (86.4) | 84 (75.7) | 35 (87.5) | 100 (79.4) | 33 (78.6) | 41 (74.5) | 50 (82.0) | 44 (67.7) | 39 (78.0) | 35 (83.3) | 38 (71.7) |
| ICU length of stay, median (IQR) | 9 (6 – 16) | **13 (7 – 22)** | 7 (5 – 10) | 6 (3 – 9) | **3 (2 – 4)** | 5 (3 – 9) | 5 (3 – 7) | 8 (4 – 13) | 8 (5 – 12) | 10 (6 – 16) | 6 (3 – 8) |
| Hospital length of stay, median (IQR) | 24 (12 – 41) | **37 (27 – 76)** | 13 (9 – 18) | 11 (3 – 23) | 8 (5 – 11) | 9 (3 – 15) | 12 (7 – 22) | 31 (21 – 45) | 21 (16 – 37) | 27 (20 – 43) | 11 (6 – 16) |
| Received invasive mechanical ventilation | 137 (77.8) | 89 (80.2) | 36 (90.0) | 89 (70.6) | 10 (23.8) | 39 (70.9) | 13 (21.3) | 50 (76.9) | 39 (78.0) | 32 (76.2) | 44 (83.0) |
| Blood culture collected | 175 (99.4) | 111 (100.0) | 38 (95.0) | 122 (96.8) | 39 (92.9) | 49 (89.1) | 58 (95.1) | 59 (90.8) | 46 (92.0) | 38 (90.5) | 44 (83.0) |
| Received antimicrobial medications | 176 (100.0) | 111 (100.0) | 40 (100.0) | 126 (100.0) | 41 (97.6) | 52 (94.5) | 61 (100.0) | 65 (100.0) | 50 (100.0) | 41 (97.6) | 51 (96.2) |
| - for 2 or more consecutive days | 175 (99.4) | 111 (100.0) | 40 (100.0) | 125 (99.2) | 40 (95.2) | 53 (96.4) | 58 (95.1) | 64 (98.5) | 50 (100.0) | 42 (100.0) | 52 (98.1) |
| Discharged Deceased | 75 (42.6) | 26 (23.4) | 6 (15.0) | **74 (58.7)** | **4 (9.5)** | **29 (52.7)** | 14 (23.0) | 10 (15.4) | 5 (10.0) | 5 (11.9) | 7 (13.2) |
| ***Acute organ dysfunction: (Any one of the following ± 2 days of blood culture)*** | | | | | | | | | | | |
| Number of acute organ dysfunctions, median (IQR) | **4 (4 – 5)** | **4 (3 – 4)** | 3 (2 – 3) | 3 (2 – 4) | 2 (1 – 2) | 3 (2 – 4) | 2 (1 – 3) | 3 (2 – 3) | 2 (1 – 3) | 3 (2 – 3) | 2 (1 – 2) |
| Respiratory (IMV) | 106 (60.2) | 68 (61.3) | **30 (75.0)** | 57 (45.2) | 6 (14.3) | 33 (60.0) | 9 (14.8) | 31 (47.7) | 31 (62.0) | 26 (61.9) | 35 (66.0) |
| Renal | **153 (86.9)** | 77 (69.4) | 18 (45.0) | 64 (50.8) | 8 (19.0) | 27 (49.1) | 35 (57.4) | 35 (53.8) | 9 (18.0) | 17 (40.5) | 9 (17.0) |
| Liver | **73 (41.5)** | 15 (13.5) | 0 (0.0) | 18 (14.3) | 6 (14.3) | 4 (7.3) | 7 (11.5) | 5 (7.7) | 1 (2.0) | 4 (9.5) | 2 (3.8) |
| Hematologic | **103 (58.5)** | 44 (39.6) | 6 (15.0) | 41 (32.5) | 10 (23.8) | 18 (32.7) | 16 (26.2) | 24 (36.9) | 6 (12.0) | 19 (45.2) | 4 (7.5) |
| Cardiac dysfunction (received intravenous vasopressor therapy) | 133 (75.6) | **86 (77.5)** | 27 (67.5) | 83 (65.9) | 17 (40.5) | 33 (60.0) | 34 (55.7) | 32 (49.2) | 19 (38.0) | 17 (40.5) | 12 (22.6) |
| Elevated lactate | **170 (96.6)** | 100 (90.1) | 27 (67.5) | 112 (88.9) | 25 (59.5) | 53 (96.4) | 40 (65.6) | 49 (75.4) | 31 (62.0) | 35 (83.3) | 34 (64.2) |
| ***Lab test results****^3^****, median (IQR)*** | | | | | | | | | | | |
| Highest serum lactate (in mmol/L) | 7.3 (3.8 – 12.7) | 3.5 (2.5 – 5.3) | 2.5 (1.8 – 4.7) | 6.8 (3.3 – 12.7) | 2.9 (1.8 – 4.0) | 9.1 (4.5 – 12.2) | 2.9 (1.6 – 4.8) | 3.0 (2.0 – 4.9) | 2.3 (1.5 – 3.1) | 4.7 (2.6 – 7.0) | 2.9 (1.6 – 4.0) |
| Highest serum creatinine (in μmol/L) | 248 (160 – 409) | 106 (81 – 127) | 100 (84 – 133) | 300 (211 – 488) | 96 (80 – 126) | 172 (140 – 223) | 270 (179 – 397) | 113 (90 – 142) | 78 (67 – 88) | 87 (70 – 107) | 86 (69 – 100) |
| Highest/lowest serum creatinine ratio^4^ | 4.1 (2.4 – 6.3) | 2.5 (1.9 – 3.2) | 2.0 (1.7 – 2.2) | 2.0 (1.6 – 2.9) | 1.6 (1.4 – 2.0) | 1.9 (1.6 – 2.5) | 2.2 (1.7 – 3.2) | 2.0 (1.8 – 2.4) | 1.8 (1.6 – 1.9) | 1.9 (1.7 – 2.4) | 1.5 (1.3 – 1.7) |
| Highest serum total bilirubin (in μmol/L) | 38 (15 – 131) | 15 (9 – 27) | 15 (9 – 25) | 16 (9 – 39) | 14 (10 – 27) | 14 (10 – 21) | 11 (7 – 21) | 11 (8 – 23) | 10 (7 – 12) | 14 (10 – 23) | 11 (6 – 15) |
| Highest/lowest serum total bilirubin ratio^4^ | 3.4 (2 – 6.8) | 3 (1.7 – 4.2) | 1.5 (1.1 – 2.3) | 1.8 (1.3 – 3) | 1.4 (1 – 3) | 1.7 (1 – 2.3) | 1.4 (1 – 2.7) | 1.7 (1.2 – 2.4) | 1.5 (1 – 1.8) | 2.7 (1.6 – 4) | 1 (1 – 1.7) |
| Lowest serum platelet count (in 10^9^/L) | 68 (36 – 117) | 131 (80 – 177) | 149 (120 – 187) | 109 (50 – 160) | 123 (101 – 186) | 115 (74 – 184) | 139 (96 – 168) | 127 (81 – 169) | 164 (125 – 206) | 111 (80 – 154) | 168 (130 – 211) |
| Lowest/highest serum platelet count ratio^4^ | 0.2 (0.1 – 0.3) | 0.2 (0.1 – 0.3) | 0.4 (0.3 – 0.6) | 0.4 (0.3 – 0.5) | 0.5 (0.3 – 0.6) | 0.5 (0.3 – 0.6) | 0.5 (0.4 – 0.6) | 0.3 (0.2 – 0.4) | 0.4 (0.3 – 0.5) | 0.2 (0.1 – 0.3) | 0.5 (0.3 – 0.7) |
| Lowest estimated glomerular filtration rate (eGFR) | 20 (13 – 34) | 60 (45 – 83) | 63 (50 – 83) | 15 (9 – 23) | 66 (46 – 83) | 29 (22 – 43) | 18 (12 – 27) | 50 (40 – 72) | 83 (70 – 95) | 97 (78 – 111) | 79 (66 – 92) |
| Lowest/highest eGFR ratio^4^ | 0.2 (0.1 – 0.4) | 0.5 (0.4 – 0.7) | 0.6 (0.5 – 0.7) | 0.4 (0.3 – 0.6) | 0.7 (0.5 – 0.8) | 0.5 (0.3 – 0.6) | 0.4 (0.2 – 0.5) | 0.5 (0.4 – 0.7) | 0.8 (0.7 – 0.8) | 0.7 (0.6 – 0.8) | 0.8 (0.7 – 0.9) |
| Lowest P/F ratio value | 109 (66 – 181) | 137 (82 – 186) | 151 (96 – 199) | 102 (72 – 175) | 185 (144 – 276) | 114 (70 – 162) | 164 (98 – 213) | 156 (99 – 216) | 156 (110 – 227) | 175 (117 – 256) | 164 (123 – 229) |
| ***Sepsis cases by comparator definitions*** | | | | | | | | | | | |
| Adult Sepsis Event (ASE) definition | **153 (86.9)** | 93 (83.8) | 33 (82.5) | 99 (78.6) | 31 (73.8) | 40 (72.7) | 40 (65.6) | 41 (63.1) | 30 (60.0) | 23 (54.8) | **29 (54.7)** |

^1^The presence of the 31 chronic comorbid conditions was determined using the Elixhauser Comorbidity Index, defined from ICD-10-CA codes as per the methodology outlined by Quan *et al.*^22^. ^2^The ICD-10 chapters were defined by the most responsible diagnosis^25^ according to the user guide of ICD-10-CA^26^. ^3^The highest (or lowest) lab test result was the maximum (or minimum) value during the entire hospitalization; ^4^The terms ‘highest/lowest ratio’ and ‘lowest/highest ratio’ refer to the ratios of the maximum to minimum lab results, and vice versa, during the entire hospitalization.

## **eFigure B4.** Principal Component Analysis (PCA) plot with ASE-majority clusters and ASE-minority clusters identified by Robust and Sparse K-means Clustering (RSKC) for the development cohort.

**A) ASE-majority clusters and ASE-minority clusters**


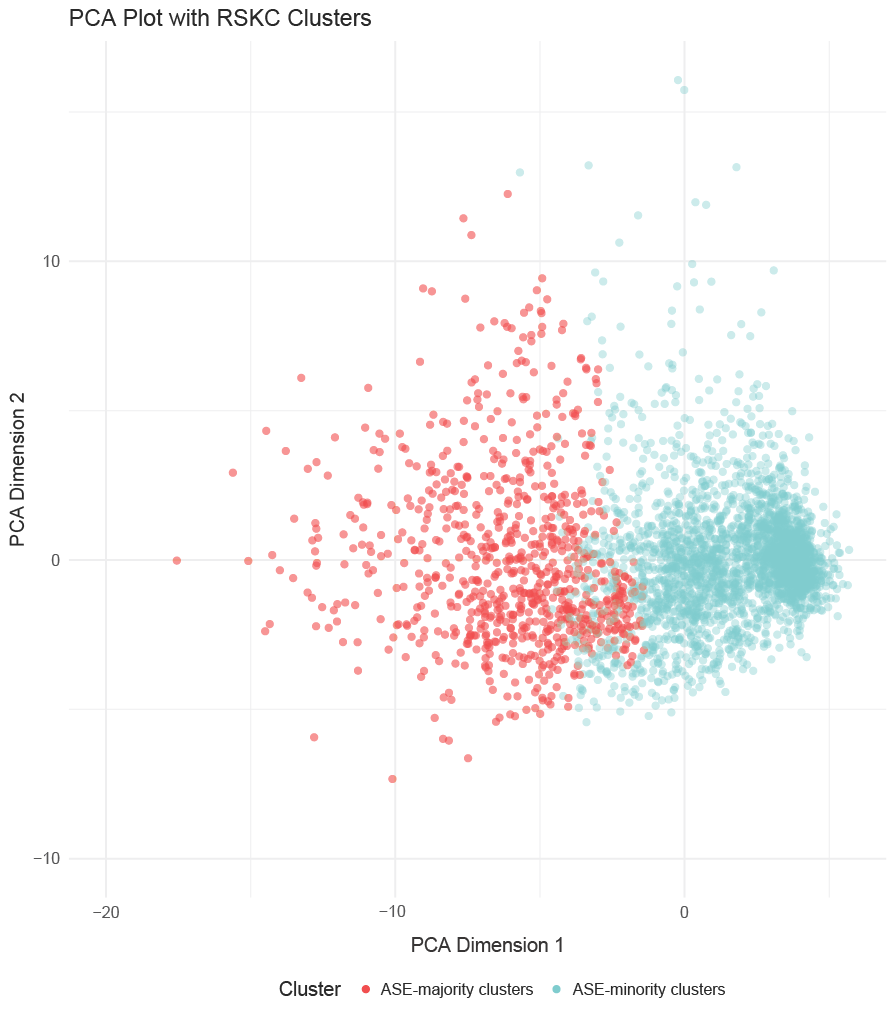


**B) ASE cases in the ASE-majority clusters, non-ASE cases in the ASE-majority clusters, ASE cases in the ASE-minority clusters, and non-ASE cases in the ASE-minority clusters**


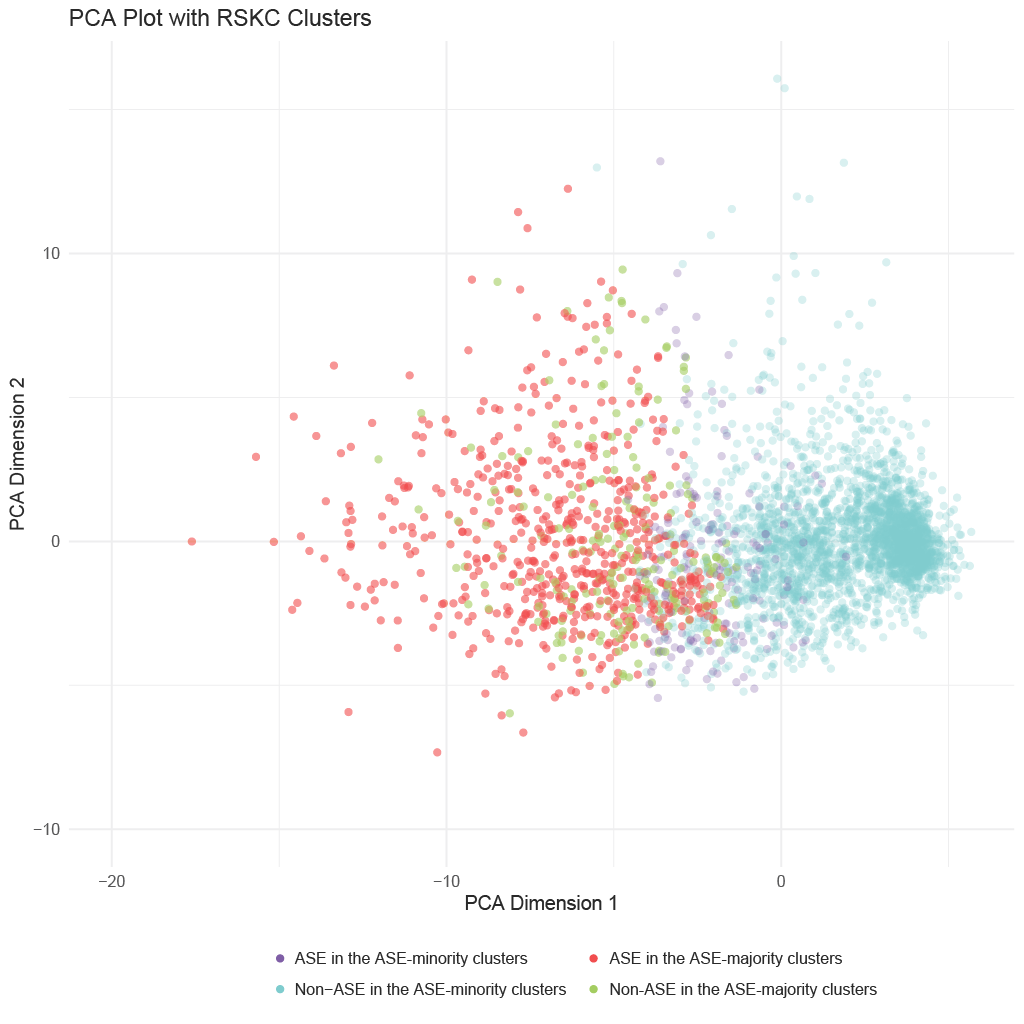


## **eFigure B5.** Laboratory test result distributions between ASE(+) [Adult Sepsis Event] and ASE(-) cases among the sepsis patients identified by Robust and Sparse K-means Clustering (RSKC) from the development cohort.

**
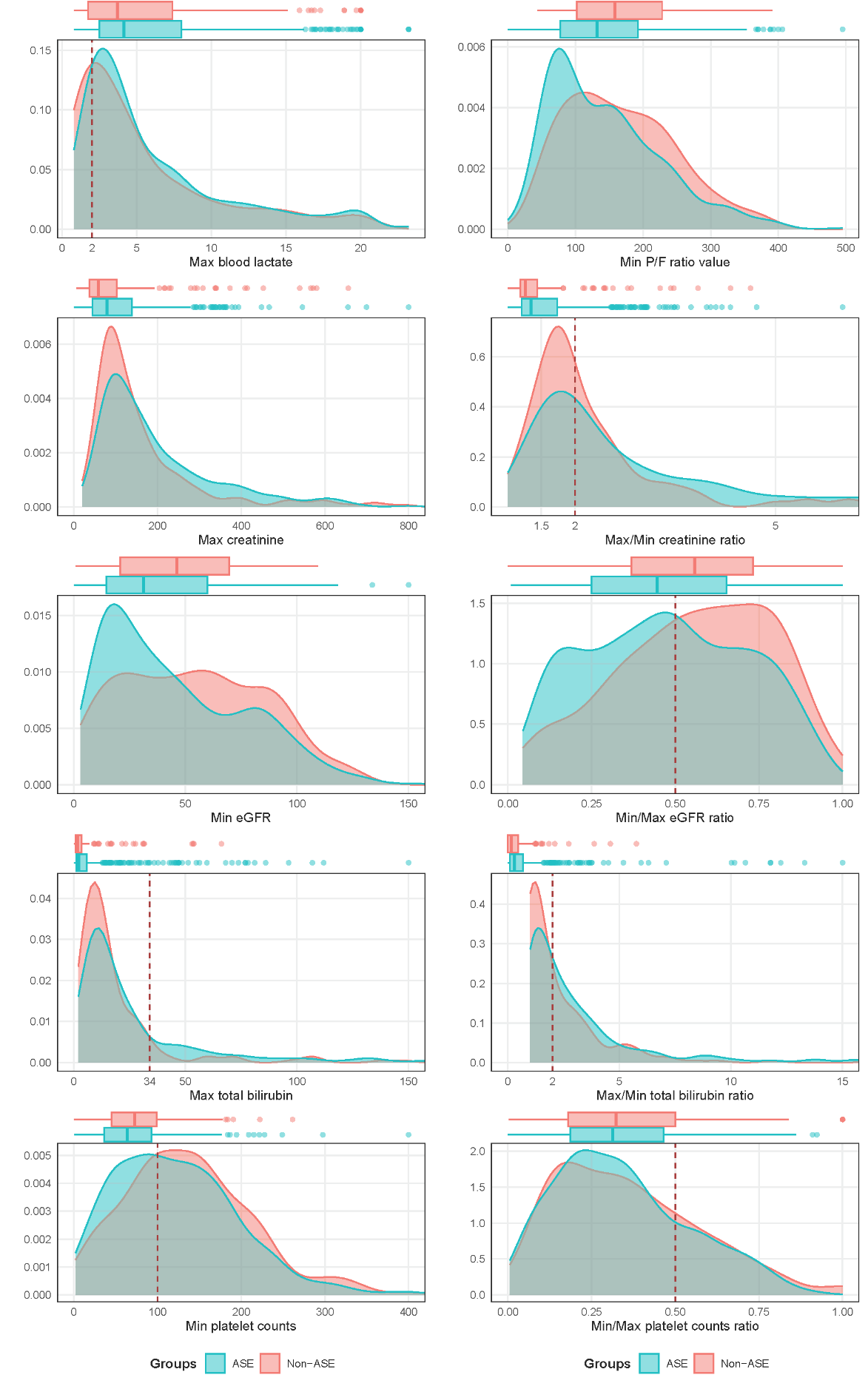
**

The ‘Max’ (or ‘Min’) lab test result was the maximum (or minimum) value during the entire hospitalization. The terms ‘Max/Min ratio’ and ‘Min/Max ratio’ refer to the ratios of the maximum to minimum lab results, and vice versa, during the entire hospitalization.

## **eFigure B6.** Laboratory test result distributions between ASE(+) [Adult Sepsis Event] and ASE(-) cases among the sepsis patients identified by Robust and Sparse K-means Clustering (RSKC) from the validation cohort.


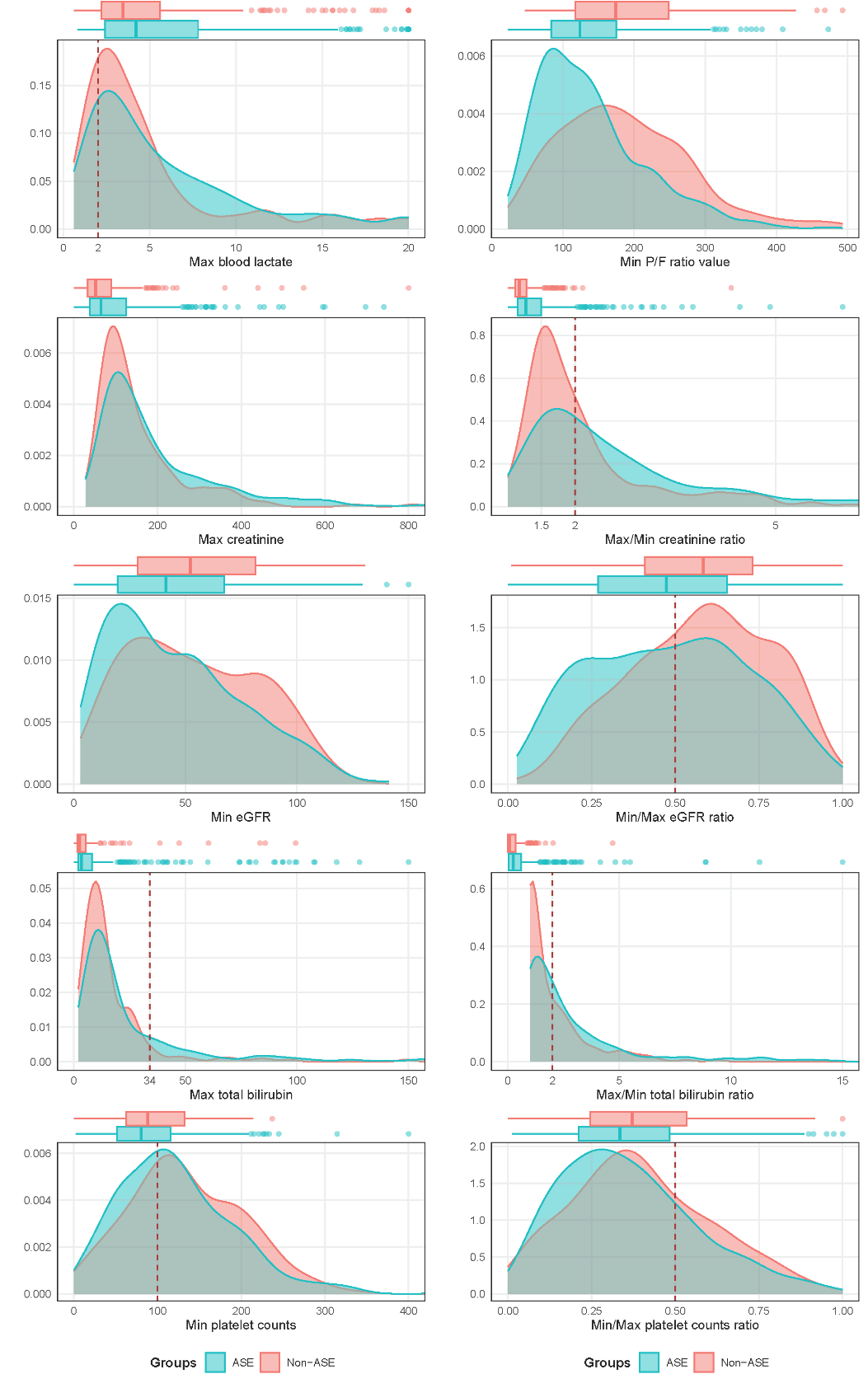


## **eFigure B7.** Percentages of ASE(+) (Adult Sepsis Event) cases in the 48 clusters for the development (x-axis) and validation (y-axis) cohorts.

**
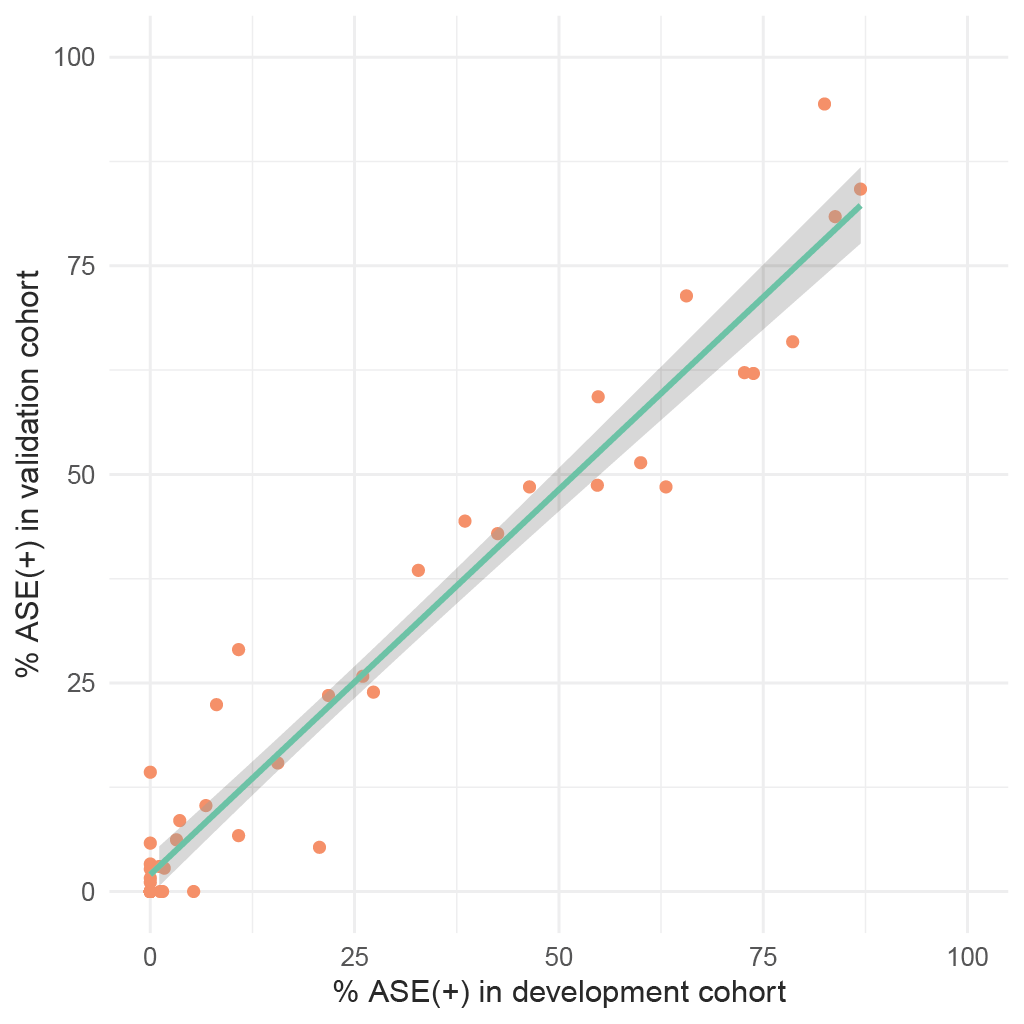
**

## **eTable B8.** Patient characteristics of the ASE(+) [Adult Sepsis Event] patients in the 11 ASE-majority clusters (ASEmac) versus the 37 ASE-minority clusters (ASEmic) in the development cohort. Values are n (%) unless otherwise indicated.

| **Characteristic** | **ASE(+) cases**  **in the ASEmac** | **ASE(+) cases**  **in the ASEmic** | **Standardized difference** |
| --- | --- | --- | --- |
|  | **N = 612** | **N= 175** |  |
| Age, median (interquartile range [IQR]) | 61 (50 – 71) | 60 (39 – 73) | -0.15 |
| Female | 268 (43.8) | 65 (37.1) | 0.14 |
| ***Clinical characteristics during hospitalization*** | | | |
| Admitted via Emergency Department | 504 (82.4) | 130 (74.3) | -0.20 |
| ICU length of stay, median (IQR) | 7 (4 – 12) | 4 (3 – 7) | -0.55** |
| Hospital length of stay, median (IQR) | 19 (8 – 34) | 10 (6 – 16) | -0.41** |
| Received invasive mechanical ventilation (IMV) | 440 (71.9) | 94 (53.7) | 0.38* |
| Blood culture collected | 612 (100.0) | 175 (100.0) | 0 |
| Received antimicrobial medications | 612 (100.0) | 175 (100.0) | 0 |
| Died during hospitalization | 201 (32.8) | 32 (18.3) | 0.34* |
| ***Acute organ dysfunction per ASE criteria: (Any one of the following ± 2 days of blood culture)*** | | | |
| Number of acute organ dysfunctions, median (IQR) | 3 (2 – 4) | 2 (1 – 2) | -1.13*** |
| Respiratory (invasive mechanical ventilation) | 345 (56.4) | 73 (41.7) | 0.29* |
| Renal | 359 (58.7) | 40 (22.9) | 0.75** |
| Liver | 114 (18.6) | 10 (5.7) | 0.41** |
| Hematologic | 230 (37.6) | 41 (23.4) | 0.31* |
| Cardiac dysfunction (received intravenous vasopressor) | 408 (66.7) | 52 (29.7) | 0.76** |
| Elevated lactate | 528 (86.3) | 119 (68.0) | 0.44** |
| ***Lab test results, median (IQR)*** | | | |
| Highest serum lactate (in mmol/L) | 4.2 (2.5 – 8.0) | 2.8 (1.7 – 5.1) | -0.48** |
| Highest serum creatinine (in μmol/L) | 156 (97 – 269) | 92 (72 – 133) | -0.56** |
| Highest/lowest serum creatinine ratio | 2.2 (1.7 – 3.6) | 1.5 (1.3 – 1.8) | -0.73** |
| Lowest estimated glomerular filtration rate (eGFR) | 37 (18 – 69) | 69 (43 – 97) | 0.80** |
| Lowest/highest eGFR ratio | 0.5 (0.3 – 0.7) | 0.7 (0.6 – 0.9) | 1.00*** |
| Highest serum total bilirubin (in μmol/L) | 16 (10 – 39) | 10 (7 – 18) | -0.35* |
| Highest/lowest serum total bilirubin ratio | 2.2 (1.3 – 3.7) | 1.0 (1.0 – 1.7) | -0.42** |
| Lowest serum platelet count (in 10^9^/L) | 117 (67 – 168) | 150 (96 – 201) | 0.43** |
| Lowest/highest serum platelet count ratio | 0.3 (0.2 – 0.5) | 0.6 (0.4 – 0.7) | 1.02*** |
| Lowest P/F ratio value | 132 (78 – 193) | 187 (112 – 260) | 0.59** |

ASEmac, clusters with ≥50% of patients meeting ASE criteria; ASEmic, clusters with <50% of patients meeting ASE criteria; Thresholds for the absolute values of standardized differences: 0≤d≤0.2 negligible effect size; 0.2<d≤0.4 small effect size (*); 0.4<d≤0.8 medium effect size (**); d>0.8 large effect size (***).

## **eTable B9.** Independent, blinded clinical evaluation and categorization of the 48 clusters from the development cohort into eight clinical categories using cluster-specific summary statistics of patient characteristics.

1. **Septic shock clusters**

| ***Cluster#*** | ***Age***^1^ | ***Degree of***  ***hypoxemia***^1^ | ***Predominantly***  ***hospital onset*** | ***Notable***  ***laboratory***  ***abnormalities*** | ***Organ***  ***dysfunctions*** | ***Common concomitant acute diagnoses*** | ***Other features*** |
| --- | --- | --- | --- | --- | --- | --- | --- |
| 8 | younger | severe |  |  | hepatic |  |  |
| 4 | younger | -- |  |  |  | drug-related/psychiatric |  |
| 5 |  | severe |  |  | severe renal | diverse |  |
| 9 |  | severe |  | very elevated lactate |  | cardiovascular, neurologic |  |
| 26 |  |  |  |  | severe renal |  | gastrointestinal source of sepsis; few required mechanical ventilation |
| 33 |  |  | √ | thrombocytosis |  |  | long LOS |
| 35 |  |  | √ |  |  | diverse | long LOS |

^1^relative to all clusters in this category; LOS, hospital length of stay.

1. **Sepsis without shock clusters**

| ***Cluster#*** | ***Age***^1^ | ***Common concomitant acute diagnoses*** | ***Other features*** |
| --- | --- | --- | --- |
| 12 |  | drug-related/psychiatric | pulmonary origin of sepsis |
| 48 |  | pulmonary, drug-related/psychiatric, stroke |  |
| 10 | older | hospital-acquired infections |  |

^1^relative to all clusters in this category.

1. **Respiratory clusters**

| ***Cluster#*** | ***Age****^1^* | ***Features*** |
| --- | --- | --- |
| 20 |  | hypercapnia and hypoxemia, suspected infection |
| 28 | younger | hypoxemia with mixture of etiologies |

^1^relative to all clusters in this category.

1. **Substance abuse/drug overdose clusters**

| ***Cluster#*** | ***Age^1^*** | ***Suspected***  ***infection*** | ***Female***  ***predominance*** | ***LOS***^1^ | ***Other features*** |
| --- | --- | --- | --- | --- | --- |
| 19 | older |  |  | short | high mortality |
| 30 |  |  |  |  |  |
| 40 |  | √ |  |  | high use of mechanical ventilation |
| 22 |  | √ |  |  | very elevated lactate; hypernatremia |
| 32 |  |  | √ |  | elevated lactate |
| 36 |  |  | √ | long | hospital-acquired infections common |
| 46 |  |  | √ |  | marked hypoxemia |
| 45 |  |  |  | long | marked hypoxemia, hospital-acquired infections common |

^1^relative to all clusters in this category; LOS, hospital length of stay.

1. **Acute myocardial infarction**

| ***Cluster#*** | ***Age^1^*** | ***Renal dysfunction*** | ***Lactate elevation*** | ***Common concomitant acute diagnoses*** |
| --- | --- | --- | --- | --- |
| 31 | much older | severe |  |  |
| 24 | older |  |  |  |
| 34 | older | moderate |  | cardiovascular |
| 13 | older |  |  | arrythmias |
| 3 |  |  | moderate |  |
| 47 |  |  | slight |  |
| 25  37 |  |  |  |  |
| 21  29 | younger |  |  |  |

^1^relative to all clusters in this category.

1. **Post-operative cardiovascular disorders**

| ***Cluster#*** | ***Age^1^*** | ***Cardiovascular disorder type prevalence*** |
| --- | --- | --- |
| 43 |  | valvular |
| 7  27 | younger | valvular |
| 11 | younger | None (diverse) |

^1^relative to all clusters in this category.

1. **Mixed cardiovascular disorders**

| ***Cluster#*** | ***Age^1^*** | ***Prevalence of acute cardiovascular disorders*** | ***Suspected***  ***infection*** | ***Renal***  ***impairment*** | ***Other features*** |
| --- | --- | --- | --- | --- | --- |
| 2 | older | CHF>arrythmia>AMI, valvular | √ |  |  |
| 14 | older | arrythmia, CHF, AMI > valvular | √ | severe |  |
| 6 | older | arrythmia, CHF, AMI > valvular | √ | moderate | elevated lactate |
| 16 | older | predominantly arrythmias |  |  |  |
| 17 | older | predominantly arrythmias | √ |  |  |
| 18 |  | diverse |  | moderate | elevated lactate, half on MV |
| 1 |  | predominantly valvular | √ |  | most on MV |
| 38 |  | CHF, AMI, arrythmia > valvular | √ | very severe |  |
| 39 | younger | predominantly CHF |  | severe |  |
| 41 | younger | predominantly valvular |  |  | elevated lactate, long LOS |

^1^relative to all clusters in this category; CHF, congestive heart failure; AMI, acute myocardial infarction; MV, mechanical ventilation, LOS, hospital length of stay.

1. **Mixed disorders**

| ***Cluster#*** | ***Age^1^*** | ***Acute disorders*** | ***Suspected***  ***infection*** | ***Other features*** |
| --- | --- | --- | --- | --- |
| 44 |  | CV, OD, COPD, DM, CA |  |  |
| 15 |  | CV, Neurologic, DM | √ | very elevated lactate, most on life support, very high mortality |
| 23 | younger | CV, OD, CA, Nerologic | √ | many on MV |
| 42 | younger | OD, COPD, CV, DM | √ | elevated lactate, many on vasopressor agents |

^1^relative to all clusters in this category; CV, cardiovascular; COPD, related to chronic obstructive pulmonary diseaese; DM, related to diabetes mellitis; CA, related to malignancy; OD, related to drugs, overdoses or psychiatric disorders; MV, mechanical ventilation.

## **eTable B10.** Patient characteristics among the 11 ASE-majority clusters between the development and validation cohort. Values are n (%) unless otherwise indicated.

| **Characteristic** | **Development Cohort**  **ASE-majority Clusters** | **Validation Cohort**  **ASE-majority Clusters** | **Standardized difference** |
| --- | --- | --- | --- |
|  | **N = 821** | **N= 611** |  |
| Age, median (interquartile range [IQR]) | 61 (51 – 72) | 61 (51 – 71) | 0.03 |
| Female | 351 (42.8) | 235 (38.5) | -0.09 |
| ***Clinical characteristics during hospitalization*** | | | |
| Admitted via Emergency Department | 651 (79.3) | 480 (78.6) | 0.04 |
| ICU length of stay, median (IQR) | 7 (4 – 12) | 7 (4 – 11) | 0.04 |
| Hospital length of stay, median (IQR) | 21 (11 – 40) | 21 (10 – 40) | 0.09 |
| Received invasive mechanical ventilation (IMV) | 577 (70.3) | 422 (69.1) | 0.02 |
| Blood culture collected | 764 (93.1) | 544 (89.0) | -0.1 |
| Received antimicrobial medications | 814 (99.1) | 590 (96.6) | -0.07 |
| Died during hospitalization | 255 (31.1) | 182 (29.8) | -0.03 |
| ***Acute organ dysfunction per ASE criteria: (Any one of the following ± 2 days of blood culture)*** | | | |
| Respiratory (invasive mechanical ventilation) | 432 (52.6) | 499 (81.7) | 0.51** |
| Renal | 452 (55.1) | 322 (52.7) | -0.07 |
| Liver | 135 (16.4) | 76 (12.4) | -0.06 |
| Hematologic | 291 (35.4) | 200 (32.7) | -0.03 |
| Cardiac dysfunction (received intravenous vasopressor) | 493 (60.0) | 330 (54.0) | -0.07 |
| ***Lab test results, median (IQR)*** | | | |
| Highest serum lactate (in mmol/L) | 4.0 (2.3 – 7.8) | 4.0 (2.3 – 7.5) | 0.02 |
| Highest serum creatinine (in μmol/L) | 146 (92 – 253) | 140 (93 – 247) | 0.03 |
| Highest/lowest serum creatinine ratio | 2.1 (1.7 – 3.3) | 2.1 (1.6 – 3.1) | 0.08 |
| Lowest estimated glomerular filtration rate (eGFR) | 40 (19 – 71) | 42 (21 – 71) | 0 |
| Lowest/highest eGFR ratio | 0.5 (0.3 – 0.7) | 0.5 (0.3 – 0.7) | -0.06 |
| Highest serum total bilirubin (in μmol/L) | 15 (9 – 31) | 14 (9 – 27) | 0.05 |
| Highest/lowest serum total bilirubin ratio | 2.0 (1.2 – 3.5) | 1.8 (1.0 – 3.1) | 0.06 |
| Lowest serum platelet count (in 10^9^/L) | 119 (72 – 171) | 118 (77 – 171) | -0.02 |
| Lowest/highest serum platelet count ratio | 0.3 (0.2 – 0.5) | 0.3 (0.2 – 0.5) | -0.09 |
| Lowest P/F ratio value | 138 (82 – 202) | 135 (88 – 202) | -0.06 |
| ***Sepsis cases by comparator definitions*** | | | |
| Adult Sepsis Event (ASE) definition | 612 (74.5) | 419 (68.6) | -0.06 |

ASE-majority clusters, clusters with ≥50% of patients meeting ASE criteria; Thresholds for the absolute values of standardized differences: 0≤d≤0.2 negligible effect size; 0.2<d≤0.4 small effect size (*); 0.4<d≤0.8 medium effect size (**); d>0.8 large effect size (***).

# Appendix C. Handling Missing Values and Variable Description

## **eTable C1.** Methods for missing data imputation. The mean imputation method was used in our unsupervised learning approach, primarily for missing patient-specific laboratory test results.

| **Aspect** | **Mean Imputation** |
| --- | --- |
| Patient-specific missing data | Fills missing values using the average result from other patients within the same cohort. |
| Assumption of similarity | Assumes the average value from other patients is a reasonable estimate for the missing value. |
| Advantages | - Easy to implement and understand. - Maintains sample size. - Quick to compute. |
| Disadvantages | - Potential for misrepresentation. - Underestimation of variability. - Ignores correlations between variables. - Not suitable for skewed distributions or when data is not missing at random. |
| Reasons for using mean imputation in unsupervised learning | This method was used to pre-process the data before our unsupervised learning approach.   - Less focus on precision for each individual data point. - Unsupervised tasks often involve large data volumes where simpler methods are more practical. |

## **eTable C2.** Description of the 592 processed variables and the 231 variables included in the model development.

| **Coded Name** | **Category** | **Feature Label** | **Data Source** | **Format** | **Included for Analysis** | **Reason for exclusion** | **% of Missing Values** |
| --- | --- | --- | --- | --- | --- | --- | --- |
| age | Demographics | Age at admission | DAD | numeric | Y |  | 0 |
| female | Demographics | Female sex | DAD | 0/1 | Y |  | 0 |
| zone | Demographics | Residential zone | DAD | categorical | N | 2, 4 |  |
| chronic_1 | Diagnoses | Congestive Heart Failure (chronic) | DAD | 0/1 | Y |  | 0 |
| chronic_10 | Diagnoses | Chronic Pulmonary Disease (chronic) | DAD | 0/1 | Y |  | 0 |
| chronic_11 | Diagnoses | Diabetes Uncomplicated (chronic) | DAD | 0/1 | Y |  | 0 |
| chronic_12 | Diagnoses | Diabetes Complicated (chronic) | DAD | 0/1 | Y |  | 0 |
| chronic_13 | Diagnoses | Hypothyroidism (chronic) | DAD | 0/1 | Y |  | 0 |
| chronic_14 | Diagnoses | Renal Failure (chronic) | DAD | 0/1 | Y |  | 0 |
| chronic_15 | Diagnoses | Liver Disease (chronic) | DAD | 0/1 | Y |  | 0 |
| chronic_16 | Diagnoses | Peptic Ulcer Disease excluding bleeding (chronic) | DAD | 0/1 | Y |  | 0 |
| chronic_18 | Diagnoses | Lymphoma (chronic) | DAD | 0/1 | Y |  | 0 |
| chronic_19 | Diagnoses | Metastatic Cancer (chronic) | DAD | 0/1 | Y |  | 0 |
| chronic_2 | Diagnoses | Cardiac Arrhythmia (chronic) | DAD | 0/1 | Y |  | 0 |
| chronic_20 | Diagnoses | Solid Tumor without Metastasis (chronic) | DAD | 0/1 | Y |  | 0 |
| chronic_21 | Diagnoses | Rheumatoid Arthritis/collagen (chronic) | DAD | 0/1 | Y |  | 0 |
| chronic_22 | Diagnoses | Coagulopathy (chronic) | DAD | 0/1 | Y |  | 0 |
| chronic_23 | Diagnoses | Obesity (chronic) | DAD | 0/1 | Y |  | 0 |
| chronic_24 | Diagnoses | Weight Loss (chronic) | DAD | 0/1 | Y |  | 0 |
| chronic_25 | Diagnoses | Fluid and Electrolyte Disorders (chronic) | DAD | 0/1 | Y |  | 0 |
| chronic_26 | Diagnoses | Blood Loss Anemia (chronic) | DAD | 0/1 | Y |  | 0 |
| chronic_27 | Diagnoses | Deficiency Anemia (chronic) | DAD | 0/1 | Y |  | 0 |
| chronic_28 | Diagnoses | Alcohol Abuse (chronic) | DAD | 0/1 | Y |  | 0 |
| chronic_29 | Diagnoses | Drug Abuse (chronic) | DAD | 0/1 | Y |  | 0 |
| chronic_3 | Diagnoses | Valvular Disease (chronic) | DAD | 0/1 | Y |  | 0 |
| chronic_30 | Diagnoses | Psychoses (chronic) | DAD | 0/1 | Y |  | 0 |
| chronic_31 | Diagnoses | Depression (chronic) | DAD | 0/1 | Y |  | 0 |
| chronic_32 | Diagnoses | Primary Immunodeficiency States (chronic) | DAD | 0/1 | Y |  | 0 |
| chronic_33 | Diagnoses | History of Organ Transplantation (chronic) | DAD | 0/1 | Y |  | 0 |
| chronic_34 | Diagnoses | Auto-Immune Disorders (chronic) | DAD | 0/1 | Y |  | 0 |
| chronic_35 | Diagnoses | Chapter miscellaneous comorbidity (chronic) | DAD | 0/1 | Y |  | 0 |
| chronic_4 | Diagnoses | Pulmonary Circulation Disorders (chronic) | DAD | 0/1 | Y |  | 0 |
| chronic_5 | Diagnoses | Peripheral Vascular Disorders (chronic) | DAD | 0/1 | Y |  | 0 |
| chronic_6 | Diagnoses | Hypertension Uncomplicated (chronic) | DAD | 0/1 | Y |  | 0 |
| chronic_7 | Diagnoses | Hypertension Complicated (chronic) | DAD | 0/1 | Y |  | 0 |
| chronic_8 | Diagnoses | Paralysis (chronic) | DAD | 0/1 | Y |  | 0 |
| chronic_9 | Diagnoses | Other Neurological Disorders (chronic) | DAD | 0/1 | Y |  | 0 |
| ELX_All_1 | Diagnoses | Congestive Heart Failure | DAD/NACRS | 0/1 | Y |  | 0 |
| ELX_All_10 | Diagnoses | Chronic Pulmonary Disease | DAD/NACRS | 0/1 | Y |  | 0 |
| ELX_All_11 | Diagnoses | Diabetes Uncomplicated | DAD/NACRS | 0/1 | Y |  | 0 |
| ELX_All_12 | Diagnoses | Diabetes Complicated | DAD/NACRS | 0/1 | Y |  | 0 |
| ELX_All_13 | Diagnoses | Hypothyroidism | DAD/NACRS | 0/1 | Y |  | 0 |
| ELX_All_14 | Diagnoses | Renal Failure | DAD/NACRS | 0/1 | Y |  | 0 |
| ELX_All_15 | Diagnoses | Liver Disease | DAD/NACRS | 0/1 | Y |  | 0 |
| ELX_All_16 | Diagnoses | Peptic Ulcer Disease, excluding bleeding | DAD/NACRS | 0/1 | Y |  | 0 |
| ELX_All_18 | Diagnoses | Lymphoma | DAD/NACRS | 0/1 | Y |  | 0 |
| ELX_All_19 | Diagnoses | Metastatic Cancer | DAD/NACRS | 0/1 | Y |  | 0 |
| ELX_All_2 | Diagnoses | Cardiac Arrhythmia | DAD/NACRS | 0/1 | Y |  | 0 |
| ELX_All_20 | Diagnoses | Solid Tumor without Metastasis | DAD/NACRS | 0/1 | Y |  | 0 |
| ELX_All_21 | Diagnoses | Rheumatoid Arthritis/collagen | DAD/NACRS | 0/1 | Y |  | 0 |
| ELX_All_22 | Diagnoses | Coagulopathy | DAD/NACRS | 0/1 | Y |  | 0 |
| ELX_All_23 | Diagnoses | Obesity | DAD/NACRS | 0/1 | Y |  | 0 |
| ELX_All_24 | Diagnoses | Weight Loss | DAD/NACRS | 0/1 | Y |  | 0 |
| ELX_All_25 | Diagnoses | Fluid and Electrolyte Disorders | DAD/NACRS | 0/1 | Y |  | 0 |
| ELX_All_26 | Diagnoses | Blood Loss Anemia | DAD/NACRS | 0/1 | Y |  | 0 |
| ELX_All_27 | Diagnoses | Deficiency Anemia | DAD/NACRS | 0/1 | Y |  | 0 |
| ELX_All_28 | Diagnoses | Alcohol Abuse | DAD/NACRS | 0/1 | Y |  | 0 |
| ELX_All_29 | Diagnoses | Drug Abuse | DAD/NACRS | 0/1 | Y |  | 0 |
| ELX_All_3 | Diagnoses | Valvular Disease | DAD/NACRS | 0/1 | Y |  | 0 |
| ELX_All_30 | Diagnoses | Psychoses | DAD/NACRS | 0/1 | Y |  | 0 |
| ELX_All_31 | Diagnoses | Depression | DAD/NACRS | 0/1 | Y |  | 0 |
| ELX_All_33 | Diagnoses | End-stage Renal Disease | DAD/NACRS | 0/1 | Y |  | 0 |
| ELX_All_34 | Diagnoses | Heart attack (AMI) | DAD/NACRS | 0/1 | Y |  | 0 |
| ELX_All_35 | Diagnoses | Abnormal ECG | DAD/NACRS | 0/1 | Y |  | 0 |
| ELX_All_36 | Diagnoses | Childbirth | DAD/NACRS | 0/1 | Y |  | 0 |
| ELX_All_37 | Diagnoses | Myocardial Infarction | DAD/NACRS | 0/1 | Y |  | 0 |
| ELX_All_38 | Diagnoses | Cerebrovascular Disease | DAD/NACRS | 0/1 | Y |  | 0 |
| ELX_All_39 | Diagnoses | Dementia | DAD/NACRS | 0/1 | Y |  | 0 |
| ELX_All_4 | Diagnoses | Pulmonary Circulation Disorders | DAD/NACRS | 0/1 | Y |  | 0 |
| ELX_All_40 | Diagnoses | Peptic Ulcer Disease | DAD/NACRS | 0/1 | Y |  | 0 |
| ELX_All_41 | Diagnoses | Mild Liver Disease | DAD/NACRS | 0/1 | Y |  | 0 |
| ELX_All_42 | Diagnoses | Moderate or Severe Liver Disease | DAD/NACRS | 0/1 | Y |  | 0 |
| ELX_All_43 | Diagnoses | Lymphoid Leukemia | DAD/NACRS | 0/1 | Y |  | 0 |
| ELX_All_45 | Diagnoses | Palliative Care | DAD/NACRS | 0/1 | Y |  | 0 |
| ELX_All_48 | Diagnoses | Non-Follicular Lymphoma | DAD/NACRS | 0/1 | Y |  | 0 |
| ELX_All_49 | Diagnoses | Other Unspecified Non-Hodgkin Lymphoma | DAD/NACRS | 0/1 | Y |  | 0 |
| ELX_All_5 | Diagnoses | Peripheral Vascular Disorders | DAD/NACRS | 0/1 | Y |  | 0 |
| ELX_All_52 | Diagnoses | Other Aplastic Anemia | DAD/NACRS | 0/1 | Y |  | 0 |
| ELX_All_57 | Diagnoses | Purpura | DAD/NACRS | 0/1 | Y |  | 0 |
| ELX_All_58 | Diagnoses | Disseminated Intravascular Coagulation | DAD/NACRS | 0/1 | Y |  | 0 |
| ELX_All_59 | Diagnoses | Other Coagulation Defects | DAD/NACRS | 0/1 | Y |  | 0 |
| ELX_All_6 | Diagnoses | Hypertension Uncomplicated | DAD/NACRS | 0/1 | Y |  | 0 |
| ELX_All_64 | Diagnoses | Systemic Lupus Erythematosus | DAD/NACRS | 0/1 | Y |  | 0 |
| ELX_All_66 | Diagnoses | Folate Deficiency Anemia | DAD/NACRS | 0/1 | Y |  | 0 |
| ELX_All_67 | Diagnoses | Iron Deficiency Anemia | DAD/NACRS | 0/1 | Y |  | 0 |
| ELX_All_68 | Diagnoses | Hospital-acquired infections | DAD/NACRS | 0/1 | Y |  | 0 |
| ELX_All_69 | Diagnoses | Decubitus ulcer | DAD/NACRS | 0/1 | Y |  | 0 |
| ELX_All_7 | Diagnoses | Hypertension Complicated | DAD/NACRS | 0/1 | Y |  | 0 |
| ELX_All_70 | Diagnoses | Endocrine & metabolic complications | DAD/NACRS | 0/1 | Y |  | 0 |
| ELX_All_71 | Diagnoses | Drug-related adverse events | DAD/NACRS | 0/1 | Y |  | 0 |
| ELX_All_72 | Diagnoses | Fluid management adverse events | DAD/NACRS | 0/1 | Y |  | 0 |
| ELX_All_73 | Diagnoses | Surgical Complications | DAD/NACRS | 0/1 | Y |  | 0 |
| ELX_All_74 | Diagnoses | Traumatic injuries in hospital | DAD/NACRS | 0/1 | Y |  | 0 |
| ELX_All_75 | Diagnoses | Anesthesia-related complications | DAD/NACRS | 0/1 | Y |  | 0 |
| ELX_All_76 | Diagnoses | Delirium | DAD/NACRS | 0/1 | Y |  | 0 |
| ELX_All_77 | Diagnoses | Central nervous system complications | DAD/NACRS | 0/1 | Y |  | 0 |
| ELX_All_78 | Diagnoses | Severe events proximally threatening to life | DAD/NACRS | 0/1 | Y |  | 0 |
| ELX_All_79 | Diagnoses | Obstetric complications -mother | DAD/NACRS | 0/1 | Y |  | 0 |
| ELX_All_8 | Diagnoses | Paralysis | DAD/NACRS | 0/1 | Y |  | 0 |
| ELX_All_9 | Diagnoses | Other Neurological Disorders | DAD/NACRS | 0/1 | Y |  | 0 |
| hospital_acq_1 | Diagnoses | Certain infectious and parasitic diseases (post-admit comorbidity) | DAD | 0/1 | Y |  | 0 |
| hospital_acq_10 | Diagnoses | Diseases of the respiratory system (post-admit comorbidity) | DAD | 0/1 | Y |  | 0 |
| hospital_acq_11 | Diagnoses | Diseases of the digestive system (post-admit comorbidity) | DAD | 0/1 | Y |  | 0 |
| hospital_acq_12 | Diagnoses | Diseases of the skin and subcutaneous tissue (post-admit comorbidity) | DAD | 0/1 | Y |  | 0 |
| hospital_acq_13 | Diagnoses | Diseases of the musculoskeletal system and connective tissue (post-admit comorbidity) | DAD | 0/1 | Y |  | 0 |
| hospital_acq_14 | Diagnoses | Diseases of the genitourinary system (post-admit comorbidity) | DAD | 0/1 | Y |  | 0 |
| hospital_acq_15 | Diagnoses | Pregnancy, childbirth and the puerperium (post-admit comorbidity) | DAD | 0/1 | Y |  | 0 |
| hospital_acq_18 | Diagnoses | Symptoms, signs and abnormal clinical and laboratory findings, not elsewhere classified (post-admit comorbidity) | DAD | 0/1 | Y |  | 0 |
| hospital_acq_19 | Diagnoses | Injury, poisoning and other certain consequences of external causes (post-admit comorbidity) | DAD | 0/1 | Y |  | 0 |
| hospital_acq_22 | Diagnoses | Chapter miscellaneous comorbidity (post-admit comorbidity) | DAD | 0/1 | Y |  | 0 |
| hospital_acq_3 | Diagnoses | Diseases of the blood and blood-forming organs and certain disorders (post-admit comorbidity) | DAD | 0/1 | Y |  | 0 |
| hospital_acq_4 | Diagnoses | Endocrine, nutritional and metabolic diseases (post-admit comorbidity) | DAD | 0/1 | Y |  | 0 |
| hospital_acq_5 | Diagnoses | Mental and behavioural disorders (post-admit comorbidity) | DAD | 0/1 | Y |  | 0 |
| hospital_acq_6 | Diagnoses | Diseases of the nervous system (post-admit comorbidity) | DAD | 0/1 | Y |  | 0 |
| hospital_acq_7 | Diagnoses | Diseases of the eye and adnexa (post-admit comorbidity) | DAD | 0/1 | Y |  | 0 |
| hospital_acq_8 | Diagnoses | Diseases of the ear and mastoid process (post-admit comorbidity) | DAD | 0/1 | Y |  | 0 |
| hospital_acq_9 | Diagnoses | Diseases of the circulatory system (post-admit comorbidity) | DAD | 0/1 | Y |  | 0 |
| icd_chapter_1 | Diagnoses | Certain infectious and parasitic diseases | DAD | 0/1 | Y |  | 0 |
| icd_chapter_10 | Diagnoses | Diseases of the respiratory system | DAD | 0/1 | Y |  | 0 |
| icd_chapter_11 | Diagnoses | Diseases of the digestive system | DAD | 0/1 | Y |  | 0 |
| icd_chapter_12 | Diagnoses | Diseases of the skin and subcutaneous tissue | DAD | 0/1 | Y |  | 0 |
| icd_chapter_13 | Diagnoses | Diseases of the musculoskeletal system and connective tissue | DAD | 0/1 | Y |  | 0 |
| icd_chapter_14 | Diagnoses | Diseases of the genitourinary system | DAD | 0/1 | Y |  | 0 |
| icd_chapter_15 | Diagnoses | Pregnancy, childbirth and the puerperium | DAD | 0/1 | Y |  | 0 |
| icd_chapter_17 | Diagnoses | Congenital malformations, deformations and chromosomal abnormalities | DAD | 0/1 | Y |  | 0 |
| icd_chapter_18 | Diagnoses | Symptoms, signs and abnormal clinical and laboratory findings not elsewhere classified | DAD | 0/1 | Y |  | 0 |
| icd_chapter_19 | Diagnoses | Injury, poisoning and certain other consequences of external causes | DAD | 0/1 | Y |  | 0 |
| icd_chapter_2 | Diagnoses | Neoplasms | DAD | 0/1 | Y |  | 0 |
| icd_chapter_21 | Diagnoses | Factors influencing health status and contact with health services | DAD | 0/1 | Y |  | 0 |
| icd_chapter_22 | Diagnoses | Chapter Miscellaneous | DAD | 0/1 | Y |  | 0 |
| icd_chapter_3 | Diagnoses | Diseases of the blood and blood-forming organs and certain disorders | DAD | 0/1 | Y |  | 0 |
| icd_chapter_4 | Diagnoses | Endocrine, nutritional, and metabolic diseases | DAD | 0/1 | Y |  | 0 |
| icd_chapter_5 | Diagnoses | Mental and behavioural disorders | DAD | 0/1 | Y |  | 0 |
| icd_chapter_6 | Diagnoses | Diseases of the nervous system | DAD | 0/1 | Y |  | 0 |
| icd_chapter_7 | Diagnoses | Diseases of the eye and adnexa | DAD | 0/1 | Y |  | 0 |
| icd_chapter_9 | Diagnoses | Diseases of the circulatory system | DAD | 0/1 | Y |  | 0 |
| chronic_17 | Diagnoses | AIDS/HIV (chronic) | DAD | 0/1 | N | 1, 2, 4 |  |
| ELX_All_17 | Diagnoses | AIDS/HIV | DAD/NACRS | 0/1 | N | 1, 4 |  |
| ELX_All_32 | Diagnoses | Sepsis | DAD/NACRS | 0/1 | N | 5 |  |
| ELX_All_44 | Diagnoses | Myeloid Leukemia | DAD/NACRS | 0/1 | N | 1, 4 |  |
| ELX_All_46 | Diagnoses | Hodgkin Lymphoma | DAD/NACRS | 0/1 | N | 1, 4 |  |
| ELX_All_47 | Diagnoses | Follicular Lymphoma | DAD/NACRS | 0/1 | N | 1, 4 |  |
| ELX_All_50 | Diagnoses | Other Specified Lymphoma | DAD/NACRS | 0/1 | N | 1, 4 |  |
| ELX_All_51 | Diagnoses | Acquired Red Cell Aplasia | DAD/NACRS | 0/1 | N | 1, 4 |  |
| ELX_All_53 | Diagnoses | Other Leukemia | DAD/NACRS | 0/1 | N | 1, 4 |  |
| ELX_All_54 | Diagnoses | Neoplasms of Uncertain Behavior | DAD/NACRS | 0/1 | N | 1, 4 |  |
| ELX_All_55 | Diagnoses | Myelodysplastic Syndromes | DAD/NACRS | 0/1 | N | 1, 4 |  |
| ELX_All_56 | Diagnoses | Polycythemia Vera | DAD/NACRS | 0/1 | N | 1, 4 |  |
| ELX_All_60 | Diagnoses | Acute Viral Hepatitis | DAD/NACRS | 0/1 | N | 1, 4 |  |
| ELX_All_61 | Diagnoses | Infectious Mononucleosis | DAD/NACRS | 0/1 | N | 1, 4 |  |
| ELX_All_62 | Diagnoses | Acquired Hemolytic Anemia | DAD/NACRS | 0/1 | N | 1, 4 |  |
| ELX_All_63 | Diagnoses | Other Necrotizing Vasculopathies | DAD/NACRS | 0/1 | N | 1, 4 |  |
| ELX_All_65 | Diagnoses | Vitamin B12 Deficiency Anemia | DAD/NACRS | 0/1 | N | 1, 4 |  |
| ELX_All_80 | Diagnoses | ICD-10-CA Coded Sepsis | DAD/NACRS | 0/1 | N | 5 |  |
| hospital_acq_16 | Diagnoses | Certain conditions originating in the perinatal period (post-admit comorbidity) | DAD | 0/1 | N | 1, 2, 4 |  |
| hospital_acq_17 | Diagnoses | Congenital malformations, deformations and chromosomal abnormalities (post-admit comorbidity) | DAD | 0/1 | N | 1, 2, 4 |  |
| hospital_acq_2 | Diagnoses | Neoplasms (post-admit comorbidity) | DAD | 0/1 | N | 1, 2, 4 |  |
| hospital_acq_20 | Diagnoses | External causes of morbidity and mortality (post-admit comorbidity) | DAD | 0/1 | N | 1, 2, 4 |  |
| hospital_acq_21 | Diagnoses | Factors influencing health status and contact with health services (post-admit comorbidity) | DAD | 0/1 | N | 1, 2, 4 |  |
| icd_chapter_16 | Diagnoses | Certain conditions originating in the perinatal period | DAD | 0/1 | N | 1, 2, 4 |  |
| icd_chapter_20 | Diagnoses | External causes of morbidity and mortality | DAD | 0/1 | N | 1, 2, 4 |  |
| icd_chapter_8 | Diagnoses | Diseases of the ear and mastoid process | DAD | 0/1 | N | 1, 2, 4 |  |
| adm_yr | Encounter | Admission year | DAD | numeric | Y |  | 0 |
| total_LoS | Encounter | Total length of stay (days) | DAD | numeric | Y |  | 0 |
| ER | Encounter | Admitted from ED | NACRS | 0/1 | Y |  | 0 |
| er_hours | Encounter | ED duration | NACRS | numeric | Y |  | 0 |
| acute_days | Encounter | Acute days | DAD | numeric | N | 5 |  |
| AdmitType | Encounter | Admission type | DAD | categorical | N | 2, 4 |  |
| AdmitCat | Encounter | Admission category | DAD | categorical | N | 2, 4 |  |
| admitdate | Encounter | Admission date | DAD | date (yymmdd) | N | 3 |  |
| admit_from | Encounter | Admitted from | DAD | categorical | N | 2, 4 |  |
| admittime | Encounter | Admission time | DAD | time | N | 3 |  |
| chestpain | Encounter | Chest pain indicated in ED data | SCM EMR | 0/1 | N | 2, 4 |  |
| chestpain_complnt_date | Encounter | Chest pain date | SCM EMR | date (yymmdd) | N | 3 |  |
| chestpain_complnt_time | Encounter | Chest pain time | SCM EMR | time | N | 3 |  |
| chestpain_txt | Encounter | Chest pain (Y/N) | SCM EMR | free text | N | 4 |  |
| death | Encounter | Death in hospital | DAD | 0/1 | N | 5 |  |
| death_scu | Encounter | Death in SCU | DAD | 0/1 | N | 5 |  |
| dischdate | Encounter | Discharge date | DAD | date (yymmdd) | N | 3 |  |
| ERdeptdate | Encounter | ED departure date | NACRS | date (yymmdd) | N | 1, 4 |  |
| ERdepttime | Encounter | ED departure time | NACRS | time | N | 1, 4 |  |
| entry_code | Encounter | Entry code | DAD | categorical | N | 2, 4 |  |
| ERadmitdate | Encounter | ED admission date | NACRS | date (yymmdd) | N | 1, 4 |  |
| ERadmittime | Encounter | ED admission time | NACRS | date (yymmdd) | N | 1, 4 |  |
| icu | Encounter | ICU stay indicator | eCritical | 0/1 | N | 5 |  |
| icu_admitdate | Encounter | ICU admission date | eCritical | date (yymmdd) | N | 1, 4 |  |
| icu_dischdate | Encounter | ICU discharge date | eCritical | date (yymmdd) | N | 1, 4 |  |
| sepsis_ase | Encounter | Adult Sepsis Event (ASE) definition indicator | SCM EMR | 0/1 | N | 5 |  |
| site | Encounter | Site | DAD | categorical | N | 2, 4 |  |
| ER_transfer_delay | Encounter | Transfer delay | NACRS | categorical | N | 1, 4 |  |
| triagedate | Encounter | Triage date | NACRS | date (yymmdd) | N | 1, 4 |  |
| triagetime | Encounter | Triage time | NACRS | time | N | 1, 4 |  |
| triagecode | Encounter | Triage code | NACRS | categorical | N | 2, 4 |  |
| nprevER | History | Number of previous ED visits | NACRS | numeric | N | 2, 4 |  |
| bcx_ind | Laboratory | Blood culture collected | SCM EMR | 0/1 | Y |  | 0 |
| enteric_culture | Laboratory | Enteric culture collected | SCM EMR | 0/1 | Y |  | 0 |
| enteye_culture | Laboratory | ENT-Eye culture collected | SCM EMR | 0/1 | Y |  | 0 |
| labs_alp_sd | Laboratory | SD of ALP values | SCM EMR | numeric | Y |  | 0 |
| labs_alt_sd | Laboratory | SD of ALT values | SCM EMR | numeric | Y |  | 0 |
| labs_bilirubin_sd | Laboratory | SD of bilirubin values | SCM EMR | numeric | Y |  | 0 |
| labs_blood_lactate_max | Laboratory | Max blood lactate value | SCM EMR | numeric | Y |  | 40.3 |
| labs_blood_lactate_sd | Laboratory | SD of blood lactate values | SCM EMR | numeric | Y |  | 0 |
| labs_cl_max | Laboratory | Max chloride value | SCM EMR | numeric | Y |  | 0.4 |
| labs_cl_min | Laboratory | Min chloride value | SCM EMR | numeric | Y |  | 0.4 |
| labs_co2_max | Laboratory | Max CO2 content value | SCM EMR | numeric | Y |  | 0.6 |
| labs_co2_min | Laboratory | Min CO2 content value | SCM EMR | numeric | Y |  | 0.6 |
| labs_creatinine_max | Laboratory | Max creatinine value | SCM EMR | numeric | Y |  | 0.6 |
| labs_creatinine_min | Laboratory | Min creatinine value | SCM EMR | numeric | Y |  | 0.6 |
| labs_creatinine_sd | Laboratory | SD of creatinine values | SCM EMR | numeric | Y |  | 0 |
| labs_FiO2_sd | Laboratory | SD of FiO2 values | SCM EMR | numeric | Y |  | 0 |
| labs_gfr_max | Laboratory | Max eGFR value | SCM EMR | numeric | Y |  | 0.8 |
| labs_gfr_min | Laboratory | Min eGFR value | SCM EMR | numeric | Y |  | 0.8 |
| labs_gfr_sd | Laboratory | SD of eGFR values | SCM EMR | numeric | Y |  | 0 |
| labs_glucose_max | Laboratory | Max glucose value | SCM EMR | numeric | Y |  | 9.6 |
| labs_glucose_min | Laboratory | Min glucose value | SCM EMR | numeric | Y |  | 9.6 |
| labs_hemato_max | Laboratory | Max hematocrit value | SCM EMR | numeric | Y |  | 0.5 |
| labs_hemato_min | Laboratory | Min hematocrit value | SCM EMR | numeric | Y |  | 0.5 |
| labs_hemoglobin_max | Laboratory | Max hemoglobin value | SCM EMR | numeric | Y |  | 0.5 |
| labs_hemoglobin_min | Laboratory | Min hemoglobin value | SCM EMR | numeric | Y |  | 0.5 |
| labs_hemoglobin_sd | Laboratory | SD of hemoglobin values | SCM EMR | numeric | Y |  | 0 |
| labs_inr_max | Laboratory | Max INR value | SCM EMR | numeric | Y |  | 19.4 |
| labs_inr_min | Laboratory | Min INR value | SCM EMR | numeric | Y |  | 19.4 |
| labs_inr_sd | Laboratory | SD of INR values | SCM EMR | numeric | Y |  | 0 |
| labs_k_max | Laboratory | Max potassium value | SCM EMR | numeric | Y |  | 0.4 |
| labs_k_min | Laboratory | Min potassium value | SCM EMR | numeric | Y |  | 0.4 |
| labs_k_sd | Laboratory | SD of potassium values | SCM EMR | numeric | Y |  | 0 |
| labs_mchc_max | Laboratory | Max MCHC value | SCM EMR | numeric | Y |  | 0.5 |
| labs_mchc_min | Laboratory | Min MCHC value | SCM EMR | numeric | Y |  | 0.5 |
| labs_mchc_sd | Laboratory | SD of MCHC values | SCM EMR | numeric | Y |  | 0 |
| labs_mcv_max | Laboratory | Max MCV value | SCM EMR | numeric | Y |  | 0.5 |
| labs_mcv_min | Laboratory | Min MCV value | SCM EMR | numeric | Y |  | 0.5 |
| labs_mcv_sd | Laboratory | SD of MCV values | SCM EMR | numeric | Y |  | 0 |
| labs_na_max | Laboratory | Max sodium value | SCM EMR | numeric | Y |  | 0.4 |
| labs_na_min | Laboratory | Min sodium value | SCM EMR | numeric | Y |  | 0.4 |
| labs_na_sd | Laboratory | SD of sodium values | SCM EMR | numeric | Y |  | 0 |
| labs_neutro_max | Laboratory | Max neutrophils value | SCM EMR | numeric | Y |  | 0.5 |
| labs_neutro_min | Laboratory | Min neutrophils value | SCM EMR | numeric | Y |  | 0.5 |
| labs_neutro_sd | Laboratory | SD of neutrophils values | SCM EMR | numeric | Y |  | 0 |
| labs_nlr_hosp_max | Laboratory | Max neutrophil to lymphocyte ratio | SCM EMR | numeric | Y |  | 0.6 |
| labs_nlr_hosp_min | Laboratory | Min neutrophil to lymphocyte ratio | SCM EMR | numeric | Y |  | 0.6 |
| labs_pco2_art_sd | Laboratory | SD of PCO2 arterial values | SCM EMR | numeric | Y |  | 0 |
| labs_PF_lab_sd | Laboratory | SD of P/F ratio values in lab data | SCM EMR | numeric | Y |  | 0 |
| labs_pltct_max | Laboratory | Max platelet counts | SCM EMR | numeric | Y |  | 0.5 |
| labs_pltct_min | Laboratory | Min platelet counts | SCM EMR | numeric | Y |  | 0.5 |
| labs_pltct_sd | Laboratory | SD of platelet counts | SCM EMR | numeric | Y |  | 0 |
| labs_ptt_max | Laboratory | Max PTT value | SCM EMR | numeric | Y |  | 26.9 |
| labs_ptt_min | Laboratory | Min PTT value | SCM EMR | numeric | Y |  | 26.9 |
| labs_ptt_sd | Laboratory | SD of PTT values | SCM EMR | numeric | Y |  | 0 |
| labs_rdw_max | Laboratory | Max RDW value | SCM EMR | numeric | Y |  | 0.5 |
| labs_rdw_min | Laboratory | Min RDW value | SCM EMR | numeric | Y |  | 0.5 |
| labs_rdw_sd | Laboratory | SD of RDW values | SCM EMR | numeric | Y |  | 0 |
| labs_troponin_max | Laboratory | Max troponin value | SCM EMR | numeric | Y |  | 21.3 |
| labs_troponin_min | Laboratory | Min troponin value | SCM EMR | numeric | Y |  | 21.3 |
| labs_urea_max | Laboratory | Max Urea value | SCM EMR | numeric | Y |  | 18.3 |
| labs_urea_min | Laboratory | Min Urea value | SCM EMR | numeric | Y |  | 18.3 |
| labs_urea_sd | Laboratory | SD of Urea values | SCM EMR | numeric | Y |  | 0 |
| labs_WBC_max | Laboratory | Max WBC value | SCM EMR | numeric | Y |  | 0.5 |
| labs_WBC_min | Laboratory | Min WBC value | SCM EMR | numeric | Y |  | 0.5 |
| resp_culture | Laboratory | Respiratory culture collected | SCM EMR | 0/1 | Y |  | 0 |
| urine_culture | Laboratory | Urine culture collected | SCM EMR | 0/1 | Y |  | 0 |
| urogenital_culture | Laboratory | Urogenital culture collected | SCM EMR | 0/1 | Y |  | 0 |
| wound_abs_culture | Laboratory | Wound-abscess culture collected | SCM EMR | 0/1 | Y |  | 0 |
| ecg | Laboratory | Taken ECG procedure | DAD | 0/1 | N | 2, 4 |  |
| lab_a1c_abnormal | Laboratory | A1C abnormal (Y/N) | SCM EMR | 0/1 | N | 1, 2, 4 |  |
| lab_a1c_high | Laboratory | A1C abnormal high (Y/N) | SCM EMR | 0/1 | N | 1, 2, 4 |  |
| lab_a1c_low | Laboratory | A1C abnormal low (Y/N) | SCM EMR | 0/1 | N | 1, 2, 4 |  |
| lab_a1c_normal | Laboratory | A1C normal (Y/N) | SCM EMR | 0/1 | N | 1, 2, 4 |  |
| lab_albumin_abnormal | Laboratory | Albumin abnormal (Y/N) | SCM EMR | 0/1 | N | 1, 2, 4 |  |
| lab_albumin_high | Laboratory | Albumin abnormal high (Y/N) | SCM EMR | 0/1 | N | 1, 2, 4 |  |
| lab_albumin_low | Laboratory | Albumin abnormal low (Y/N) | SCM EMR | 0/1 | N | 1, 2, 4 |  |
| lab_albumin_normal | Laboratory | Albumin normal (Y/N) | SCM EMR | 0/1 | N | 1, 2, 4 |  |
| lab_alp_abnormal | Laboratory | ALP abnormal (Y/N) | SCM EMR | 0/1 | N | 1, 2, 4 |  |
| lab_alp_high | Laboratory | ALP abnormal high (Y/N) | SCM EMR | 0/1 | N | 1, 2, 4 |  |
| lab_alp_low | Laboratory | ALP abnormal low (Y/N) | SCM EMR | 0/1 | N | 1, 2, 4 |  |
| lab_alp_normal | Laboratory | ALP normal (Y/N) | SCM EMR | 0/1 | N | 1, 2, 4 |  |
| lab_alt_abnormal | Laboratory | ALT abnormal (Y/N) | SCM EMR | 0/1 | N | 1, 2, 4 |  |
| lab_alt_high | Laboratory | ALT abnormal high (Y/N) | SCM EMR | 0/1 | N | 1, 2, 4 |  |
| lab_alt_normal | Laboratory | ALT normal (Y/N) | SCM EMR | 0/1 | N | 1, 2, 4 |  |
| lab_ast_abnormal | Laboratory | AST abnormal (Y/N) | SCM EMR | 0/1 | N | 1, 2, 4 |  |
| lab_ast_high | Laboratory | AST abnormal high (Y/N) | SCM EMR | 0/1 | N | 1, 2, 4 |  |
| lab_ast_low | Laboratory | AST abnormal low (Y/N) | SCM EMR | 0/1 | N | 1, 2, 4 |  |
| lab_ast_normal | Laboratory | AST normal (Y/N) | SCM EMR | 0/1 | N | 1, 2, 4 |  |
| lab_bilirubin_abnormal | Laboratory | Bilirubin abnormal (Y/N) | SCM EMR | 0/1 | N | 1, 2, 4 |  |
| lab_bilirubin_high | Laboratory | Bilirubin abnormal high (Y/N) | SCM EMR | 0/1 | N | 1, 2, 4 |  |
| lab_bilirubin_normal | Laboratory | Bilirubin normal (Y/N) | SCM EMR | 0/1 | N | 1, 2, 4 |  |
| lab_blood_lactate_abnormal | Laboratory | Blood lactate abnormal (Y/N) | SCM EMR | 0/1 | N | 1, 2, 4 |  |
| lab_blood_lactate_high | Laboratory | Blood lactate abnormal high (Y/N) | SCM EMR | 0/1 | N | 1, 2, 4 |  |
| lab_blood_lactate_low | Laboratory | Blood lactate abnormal low (Y/N) | SCM EMR | 0/1 | N | 1, 2, 4 |  |
| lab_blood_lactate_normal | Laboratory | Blood lactate normal (Y/N) | SCM EMR | 0/1 | N | 1, 2, 4 |  |
| lab_calcium_bg_abnormal | Laboratory | Ionized calcium abnormal (Y/N) | SCM EMR | 0/1 | N | 1, 2, 4 |  |
| lab_calcium_bg_high | Laboratory | Ionized calcium abnormal high (Y/N) | SCM EMR | 0/1 | N | 1, 2, 4 |  |
| lab_calcium_bg_low | Laboratory | Ionized calcium abnormal low (Y/N) | SCM EMR | 0/1 | N | 1, 2, 4 |  |
| lab_calcium_bg_normal | Laboratory | Ionized calcium normal (Y/N) | SCM EMR | 0/1 | N | 1, 2, 4 |  |
| lab_calcium_gc_abnormal | Laboratory | Total calcium abnormal (Y/N) | SCM EMR | 0/1 | N | 1, 2, 4 |  |
| lab_calcium_gc_high | Laboratory | Total calcium abnormal high (Y/N) | SCM EMR | 0/1 | N | 1, 2, 4 |  |
| lab_calcium_gc_low | Laboratory | Total calcium abnormal low (Y/N) | SCM EMR | 0/1 | N | 1, 2, 4 |  |
| lab_calcium_gc_normal | Laboratory | Total calcium normal (Y/N) | SCM EMR | 0/1 | N | 1, 2, 4 |  |
| lab_cholesterol_abnormal | Laboratory | Cholesterol abnormal (Y/N) | SCM EMR | 0/1 | N | 1, 2, 4 |  |
| lab_cholesterol_high | Laboratory | Cholesterol abnormal high (Y/N) | SCM EMR | 0/1 | N | 1, 2, 4 |  |
| lab_cholesterol_low | Laboratory | Cholesterol abnormal low (Y/N) | SCM EMR | 0/1 | N | 1, 2, 4 |  |
| lab_cholesterol_normal | Laboratory | Cholesterol normal (Y/N) | SCM EMR | 0/1 | N | 1, 2, 4 |  |
| lab_ck_abnormal | Laboratory | CK abnormal (Y/N) | SCM EMR | 0/1 | N | 1, 2, 4 |  |
| lab_ck_high | Laboratory | CK abnormal high (Y/N) | SCM EMR | 0/1 | N | 1, 2, 4 |  |
| lab_ck_normal | Laboratory | CK normal (Y/N) | SCM EMR | 0/1 | N | 1, 2, 4 |  |
| lab_cl_abnormal | Laboratory | CL abnormal (Y/N) | SCM EMR | 0/1 | N | 1, 2, 4 |  |
| lab_cl_high | Laboratory | CL abnormal high (Y/N) | SCM EMR | 0/1 | N | 1, 2, 4 |  |
| lab_cl_low | Laboratory | CL abnormal low (Y/N) | SCM EMR | 0/1 | N | 1, 2, 4 |  |
| lab_cl_normal | Laboratory | CL normal (Y/N) | SCM EMR | 0/1 | N | 1, 2, 4 |  |
| lab_co2_abnormal | Laboratory | CO2 content abnormal (Y/N) | SCM EMR | 0/1 | N | 1, 2, 4 |  |
| lab_co2_high | Laboratory | CO2 content abnormal high (Y/N) | SCM EMR | 0/1 | N | 1, 2, 4 |  |
| lab_co2_low | Laboratory | CO2 content abnormal low (Y/N) | SCM EMR | 0/1 | N | 1, 2, 4 |  |
| lab_co2_normal | Laboratory | CO2 content normal (Y/N) | SCM EMR | 0/1 | N | 1, 2, 4 |  |
| lab_creatinine_abnormal | Laboratory | Creatinine abnormal (Y/N) | SCM EMR | 0/1 | N | 1, 2, 4 |  |
| lab_creatinine_high | Laboratory | Creatinine abnormal high (Y/N) | SCM EMR | 0/1 | N | 1, 2, 4 |  |
| lab_creatinine_low | Laboratory | Creatinine abnormal low (Y/N) | SCM EMR | 0/1 | N | 1, 2, 4 |  |
| lab_creatinine_normal | Laboratory | Creatinine normal (Y/N) | SCM EMR | 0/1 | N | 1, 2, 4 |  |
| lab_crp_abnormal | Laboratory | C-Reactive Protein abnormal (Y/N) | SCM EMR | 0/1 | N | 1, 2, 4 |  |
| lab_crp_high | Laboratory | C-Reactive Protein abnormal high (Y/N) | SCM EMR | 0/1 | N | 1, 2, 4 |  |
| lab_crp_normal | Laboratory | C-Reactive Protein normal (Y/N) | SCM EMR | 0/1 | N | 1, 2, 4 |  |
| lab_ddimer_abnormal | Laboratory | D-Dimer abnormal (Y/N) | SCM EMR | 0/1 | N | 1, 2, 4 |  |
| lab_ddimer_high | Laboratory | D-Dimer abnormal high (Y/N) | SCM EMR | 0/1 | N | 1, 2, 4 |  |
| lab_ddimer_normal | Laboratory | D-Dimer normal (Y/N) | SCM EMR | 0/1 | N | 1, 2, 4 |  |
| lab_ferritin_abnormal | Laboratory | Ferritin abnormal (Y/N) | SCM EMR | 0/1 | N | 1, 2, 4 |  |
| lab_ferritin_high | Laboratory | Ferritin abnormal high (Y/N) | SCM EMR | 0/1 | N | 1, 2, 4 |  |
| lab_ferritin_low | Laboratory | Ferritin abnormal low (Y/N) | SCM EMR | 0/1 | N | 1, 2, 4 |  |
| lab_ferritin_normal | Laboratory | Ferritin normal (Y/N) | SCM EMR | 0/1 | N | 1, 2, 4 |  |
| lab_fibr_abnormal | Laboratory | Fibrinogen abnormal (Y/N) | SCM EMR | 0/1 | N | 1, 2, 4 |  |
| lab_fibr_high | Laboratory | Fibrinogen abnormal high (Y/N) | SCM EMR | 0/1 | N | 1, 2, 4 |  |
| lab_fibr_low | Laboratory | Fibrinogen abnormal low (Y/N) | SCM EMR | 0/1 | N | 1, 2, 4 |  |
| lab_fibr_normal | Laboratory | Fibrinogen normal (Y/N) | SCM EMR | 0/1 | N | 1, 2, 4 |  |
| lab_FiO2_abnormal | Laboratory | FiO2 abnormal (Y/N) | SCM EMR | 0/1 | N | 1, 2, 4 |  |
| lab_FiO2_high | Laboratory | FiO2 abnormal high (Y/N) | SCM EMR | 0/1 | N | 1, 2, 4 |  |
| lab_FiO2_low | Laboratory | FiO2 abnormal low (Y/N) | SCM EMR | 0/1 | N | 1, 2, 4 |  |
| lab_FiO2_normal | Laboratory | FiO2 normal (Y/N) | SCM EMR | 0/1 | N | 1, 2, 4 |  |
| lab_gfr_abnormal | Laboratory | eGFR abnormal (Y/N) | SCM EMR | 0/1 | N | 1, 2, 4 |  |
| lab_gfr_low | Laboratory | eGFR abnormal low (Y/N) | SCM EMR | 0/1 | N | 1, 2, 4 |  |
| lab_gfr_normal | Laboratory | eGFR normal (Y/N) | SCM EMR | 0/1 | N | 1, 2, 4 |  |
| lab_ggt_abnormal | Laboratory | GGT abnormal (Y/N) | SCM EMR | 0/1 | N | 1, 2, 4 |  |
| lab_ggt_high | Laboratory | GGT abnormal high (Y/N) | SCM EMR | 0/1 | N | 1, 2, 4 |  |
| lab_ggt_low | Laboratory | GGT abnormal low (Y/N) | SCM EMR | 0/1 | N | 1, 2, 4 |  |
| lab_ggt_normal | Laboratory | GGT normal (Y/N) | SCM EMR | 0/1 | N | 1, 2, 4 |  |
| lab_glucose_abnormal | Laboratory | Glucose abnormal (Y/N) | SCM EMR | 0/1 | N | 1, 2, 4 |  |
| lab_glucose_high | Laboratory | Glucose abnormal high (Y/N) | SCM EMR | 0/1 | N | 1, 2, 4 |  |
| lab_glucose_low | Laboratory | Glucose abnormal low (Y/N) | SCM EMR | 0/1 | N | 1, 2, 4 |  |
| lab_glucose_normal | Laboratory | Glucose normal (Y/N) | SCM EMR | 0/1 | N | 1, 2, 4 |  |
| lab_hemato_abnormal | Laboratory | Hematocrit abnormal (Y/N) | SCM EMR | 0/1 | N | 1, 2, 4 |  |
| lab_hemato_high | Laboratory | Hematocrit abnormal high (Y/N) | SCM EMR | 0/1 | N | 1, 2, 4 |  |
| lab_hemato_low | Laboratory | Hematocrit abnormal low (Y/N) | SCM EMR | 0/1 | N | 1, 2, 4 |  |
| lab_hemato_normal | Laboratory | Hematocrit normal (Y/N) | SCM EMR | 0/1 | N | 1, 2, 4 |  |
| lab_hemoglobin_abnormal | Laboratory | Hemoglobin abnormal (Y/N) | SCM EMR | 0/1 | N | 1, 2, 4 |  |
| lab_hemoglobin_high | Laboratory | Hemoglobin abnormal high (Y/N) | SCM EMR | 0/1 | N | 1, 2, 4 |  |
| lab_hemoglobin_low | Laboratory | Hemoglobin abnormal low (Y/N) | SCM EMR | 0/1 | N | 1, 2, 4 |  |
| lab_hemoglobin_normal | Laboratory | Hemoglobin normal (Y/N) | SCM EMR | 0/1 | N | 1, 2, 4 |  |
| lab_inr_abnormal | Laboratory | INR abnormal (Y/N) | SCM EMR | 0/1 | N | 1, 2, 4 |  |
| lab_inr_high | Laboratory | INR abnormal high (Y/N) | SCM EMR | 0/1 | N | 1, 2, 4 |  |
| lab_inr_low | Laboratory | INR abnormal low (Y/N) | SCM EMR | 0/1 | N | 1, 2, 4 |  |
| lab_inr_normal | Laboratory | INR normal (Y/N) | SCM EMR | 0/1 | N | 1, 2, 4 |  |
| lab_k_abnormal | Laboratory | Potassium abnormal (Y/N) | SCM EMR | 0/1 | N | 1, 2, 4 |  |
| lab_k_high | Laboratory | Potassium abnormal high (Y/N) | SCM EMR | 0/1 | N | 1, 2, 4 |  |
| lab_k_low | Laboratory | Potassium abnormal low (Y/N) | SCM EMR | 0/1 | N | 1, 2, 4 |  |
| lab_k_normal | Laboratory | Potassium normal (Y/N) | SCM EMR | 0/1 | N | 1, 2, 4 |  |
| lab_ld_abnormal | Laboratory | LD abnormal (Y/N) | SCM EMR | 0/1 | N | 1, 2, 4 |  |
| lab_ld_high | Laboratory | LD abnormal high (Y/N) | SCM EMR | 0/1 | N | 1, 2, 4 |  |
| lab_ld_low | Laboratory | LD abnormal low (Y/N) | SCM EMR | 0/1 | N | 1, 2, 4 |  |
| lab_ld_normal | Laboratory | LD normal (Y/N) | SCM EMR | 0/1 | N | 1, 2, 4 |  |
| lab_ldl_abnormal | Laboratory | LDL abnormal (Y/N) | SCM EMR | 0/1 | N | 1, 2, 4 |  |
| lab_ldl_high | Laboratory | LDL abnormal high (Y/N) | SCM EMR | 0/1 | N | 1, 2, 4 |  |
| lab_ldl_low | Laboratory | LDL abnormal low (Y/N) | SCM EMR | 0/1 | N | 1, 2, 4 |  |
| lab_ldl_normal | Laboratory | LDL normal (Y/N) | SCM EMR | 0/1 | N | 1, 2, 4 |  |
| lab_leukocyte_abnormal | Laboratory | Leukocyte abnormal (Y/N) | SCM EMR | 0/1 | N | 1, 2, 4 |  |
| lab_leukocyte_normal | Laboratory | Leukocyte normal (Y/N) | SCM EMR | 0/1 | N | 1, 2, 4 |  |
| lab_lymphs_abnormal | Laboratory | Lymphocytes abnormal (Y/N) | SCM EMR | 0/1 | N | 1, 2, 4 |  |
| lab_lymphs_high | Laboratory | Lymphocytes abnormal high (Y/N) | SCM EMR | 0/1 | N | 1, 2, 4 |  |
| lab_lymphs_low | Laboratory | Lymphocytes abnormal low (Y/N) | SCM EMR | 0/1 | N | 1, 2, 4 |  |
| lab_lymphs_normal | Laboratory | Lymphocytes normal (Y/N) | SCM EMR | 0/1 | N | 1, 2, 4 |  |
| lab_mchc_abnormal | Laboratory | MCHC abnormal (Y/N) | SCM EMR | 0/1 | N | 1, 2, 4 |  |
| lab_mchc_high | Laboratory | MCHC abnormal high (Y/N) | SCM EMR | 0/1 | N | 1, 2, 4 |  |
| lab_mchc_low | Laboratory | MCHC abnormal low (Y/N) | SCM EMR | 0/1 | N | 1, 2, 4 |  |
| lab_mchc_normal | Laboratory | MCHC normal (Y/N) | SCM EMR | 0/1 | N | 1, 2, 4 |  |
| lab_mcv_abnormal | Laboratory | MCV abnormal (Y/N) | SCM EMR | 0/1 | N | 1, 2, 4 |  |
| lab_mcv_high | Laboratory | MCV abnormal high (Y/N) | SCM EMR | 0/1 | N | 1, 2, 4 |  |
| lab_mcv_low | Laboratory | MCV abnormal low (Y/N) | SCM EMR | 0/1 | N | 1, 2, 4 |  |
| lab_mcv_normal | Laboratory | MCV normal (Y/N) | SCM EMR | 0/1 | N | 1, 2, 4 |  |
| lab_MRSA_abnormal | Laboratory | MRSA abnormal (Y/N) | SCM EMR | 0/1 | N | 1, 2, 4 |  |
| lab_MRSA_normal | Laboratory | MRSA normal (Y/N) | SCM EMR | 0/1 | N | 1, 2, 4 |  |
| lab_na_abnormal | Laboratory | Sodium abnormal (Y/N) | SCM EMR | 0/1 | N | 1, 2, 4 |  |
| lab_na_high | Laboratory | Sodium abnormal high (Y/N) | SCM EMR | 0/1 | N | 1, 2, 4 |  |
| lab_na_low | Laboratory | Sodium abnormal low (Y/N) | SCM EMR | 0/1 | N | 1, 2, 4 |  |
| lab_na_normal | Laboratory | Sodium normal (Y/N) | SCM EMR | 0/1 | N | 1, 2, 4 |  |
| lab_neutro_abnormal | Laboratory | Neutrophils abnormal (Y/N) | SCM EMR | 0/1 | N | 1, 2, 4 |  |
| lab_neutro_high | Laboratory | Neutrophils abnormal high (Y/N) | SCM EMR | 0/1 | N | 1, 2, 4 |  |
| lab_neutro_low | Laboratory | Neutrophils abnormal low (Y/N) | SCM EMR | 0/1 | N | 1, 2, 4 |  |
| lab_neutro_normal | Laboratory | Neutrophils normal (Y/N) | SCM EMR | 0/1 | N | 1, 2, 4 |  |
| lab_pco2_art_abnormal | Laboratory | PCO2 arterial abnormal (Y/N) | SCM EMR | 0/1 | N | 1, 2, 4 |  |
| lab_pco2_art_high | Laboratory | PCO2 arterial abnormal high (Y/N) | SCM EMR | 0/1 | N | 1, 2, 4 |  |
| lab_pco2_art_low | Laboratory | PCO2 arterial abnormal low (Y/N) | SCM EMR | 0/1 | N | 1, 2, 4 |  |
| lab_pco2_art_normal | Laboratory | PCO2 arterial normal (Y/N) | SCM EMR | 0/1 | N | 1, 2, 4 |  |
| lab_PF_lab_abnormal | Laboratory | P/F ratio abnormal (Y/N) | SCM EMR | 0/1 | N | 1, 2, 4 |  |
| lab_PF_lab_low | Laboratory | P/F ratio abnormal low (Y/N) | SCM EMR | 0/1 | N | 1, 2, 4 |  |
| lab_PF_lab_normal | Laboratory | P/F ratio normal (Y/N) | SCM EMR | 0/1 | N | 1, 2, 4 |  |
| lab_pltct_abnormal | Laboratory | Platelet counts abnormal (Y/N) | SCM EMR | 0/1 | N | 1, 2, 4 |  |
| lab_pltct_high | Laboratory | Platelet counts abnormal high (Y/N) | SCM EMR | 0/1 | N | 1, 2, 4 |  |
| lab_pltct_low | Laboratory | Platelet counts abnormal low (Y/N) | SCM EMR | 0/1 | N | 1, 2, 4 |  |
| lab_pltct_normal | Laboratory | Platelet counts normal (Y/N) | SCM EMR | 0/1 | N | 1, 2, 4 |  |
| lab_protein_total_abnormal | Laboratory | Protein total abnormal (Y/N) | SCM EMR | 0/1 | N | 1, 2, 4 |  |
| lab_protein_total_high | Laboratory | Protein total abnormal high (Y/N) | SCM EMR | 0/1 | N | 1, 2, 4 |  |
| lab_protein_total_low | Laboratory | Protein total abnormal low (Y/N) | SCM EMR | 0/1 | N | 1, 2, 4 |  |
| lab_protein_total_normal | Laboratory | Protein total normal (Y/N) | SCM EMR | 0/1 | N | 1, 2, 4 |  |
| lab_ptt_abnormal | Laboratory | PTT abnormal (Y/N) | SCM EMR | 0/1 | N | 1, 2, 4 |  |
| lab_ptt_high | Laboratory | PTT abnormal high (Y/N) | SCM EMR | 0/1 | N | 1, 2, 4 |  |
| lab_ptt_low | Laboratory | PTT abnormal low (Y/N) | SCM EMR | 0/1 | N | 1, 2, 4 |  |
| lab_ptt_normal | Laboratory | PTT normal (Y/N) | SCM EMR | 0/1 | N | 1, 2, 4 |  |
| lab_rdw_abnormal | Laboratory | RDW abnormal (Y/N) | SCM EMR | 0/1 | N | 1, 2, 4 |  |
| lab_rdw_high | Laboratory | RDW abnormal high (Y/N) | SCM EMR | 0/1 | N | 1, 2, 4 |  |
| lab_rdw_low | Laboratory | RDW abnormal low (Y/N) | SCM EMR | 0/1 | N | 1, 2, 4 |  |
| lab_rdw_normal | Laboratory | RDW normal (Y/N) | SCM EMR | 0/1 | N | 1, 2, 4 |  |
| lab_triglycerides_abnormal | Laboratory | Triglycerides abnormal (Y/N) | SCM EMR | 0/1 | N | 1, 2, 4 |  |
| lab_triglycerides_high | Laboratory | Triglycerides abnormal high (Y/N) | SCM EMR | 0/1 | N | 1, 2, 4 |  |
| lab_triglycerides_low | Laboratory | Triglycerides abnormal low (Y/N) | SCM EMR | 0/1 | N | 1, 2, 4 |  |
| lab_triglycerides_normal | Laboratory | Triglycerides normal (Y/N) | SCM EMR | 0/1 | N | 1, 2, 4 |  |
| lab_troponin_abnormal | Laboratory | Troponin abnormal (Y/N) | SCM EMR | 0/1 | N | 1, 2, 4 |  |
| lab_troponin_high | Laboratory | Troponin abnormal high (Y/N) | SCM EMR | 0/1 | N | 1, 2, 4 |  |
| lab_troponin_normal | Laboratory | Troponin normal (Y/N) | SCM EMR | 0/1 | N | 1, 2, 4 |  |
| lab_urea_abnormal | Laboratory | Urea abnormal (Y/N) | SCM EMR | 0/1 | N | 1, 2, 4 |  |
| lab_urea_high | Laboratory | Urea abnormal high (Y/N) | SCM EMR | 0/1 | N | 1, 2, 4 |  |
| lab_urea_low | Laboratory | Urea abnormal low (Y/N) | SCM EMR | 0/1 | N | 1, 2, 4 |  |
| lab_urea_normal | Laboratory | Urea normal (Y/N) | SCM EMR | 0/1 | N | 1, 2, 4 |  |
| labs_a1c_max | Laboratory | Max A1C value | SCM EMR | numeric | N | 1, 2, 4 |  |
| labs_a1c_mean | Laboratory | Mean A1C value | SCM EMR | numeric | N | 1, 2, 4 |  |
| labs_a1c_min | Laboratory | Min A1C value | SCM EMR | numeric | N | 1, 2, 4 |  |
| labs_a1c_sd | Laboratory | SD of A1C values | SCM EMR | numeric | N | 1, 2, 4 |  |
| labs_albumin_max | Laboratory | Max albumin value | SCM EMR | numeric | N | 1, 2, 4 |  |
| labs_albumin_mean | Laboratory | Mean albumin value | SCM EMR | numeric | N | 1, 2, 4 |  |
| labs_albumin_min | Laboratory | Min albumin value | SCM EMR | numeric | N | 1, 2, 4 |  |
| labs_albumin_sd | Laboratory | SD of albumin values | SCM EMR | numeric | N | 1, 2, 4 |  |
| labs_alp_max | Laboratory | Max ALP value | SCM EMR | numeric | N | 1, 2, 4 |  |
| labs_alp_mean | Laboratory | Mean ALP value | SCM EMR | numeric | N | 1, 2, 4 |  |
| labs_alp_min | Laboratory | Min ALP value | SCM EMR | numeric | N | 1, 2, 4 |  |
| labs_alt_max | Laboratory | Max ALT value | SCM EMR | numeric | N | 1, 2, 4 |  |
| labs_alt_mean | Laboratory | Mean ALT value | SCM EMR | numeric | N | 1, 2, 4 |  |
| labs_alt_min | Laboratory | Min ALT value | SCM EMR | numeric | N | 1, 2, 4 |  |
| labs_ast_max | Laboratory | Max AST value | SCM EMR | numeric | N | 1, 2, 4 |  |
| labs_ast_mean | Laboratory | Mean AST value | SCM EMR | numeric | N | 1, 2, 4 |  |
| labs_ast_min | Laboratory | Min AST value | SCM EMR | numeric | N | 1, 2, 4 |  |
| labs_ast_sd | Laboratory | SD of AST values | SCM EMR | numeric | N | 1, 2, 4 |  |
| labs_bilirubin_max | Laboratory | Max bilirubin value | SCM EMR | numeric | N | 1, 2, 4 |  |
| labs_bilirubin_mean | Laboratory | Mean bilirubin value | SCM EMR | numeric | N | 1, 2, 4 |  |
| labs_bilirubin_min | Laboratory | Min bilirubin value | SCM EMR | numeric | N | 1, 2, 4 |  |
| labs_blood_lactate_mean | Laboratory | Mean blood lactate value | SCM EMR | numeric | N | 1, 2, 4 |  |
| labs_blood_lactate_min | Laboratory | Min blood lactate value | SCM EMR | numeric | N | 1, 2, 4 |  |
| labs_calcium_bg_max | Laboratory | Max ionized calcium value | SCM EMR | numeric | N | 1, 2, 4 |  |
| labs_calcium_bg_mean | Laboratory | Mean ionized calcium value | SCM EMR | numeric | N | 1, 2, 4 |  |
| labs_calcium_bg_min | Laboratory | Min ionized calcium value | SCM EMR | numeric | N | 1, 2, 4 |  |
| labs_calcium_bg_sd | Laboratory | SD of ionized calcium values | SCM EMR | numeric | N | 1, 2, 4 |  |
| labs_calcium_gc_max | Laboratory | Max total calcium value | SCM EMR | numeric | N | 1, 2, 4 |  |
| labs_calcium_gc_mean | Laboratory | Mean total calcium value | SCM EMR | numeric | N | 1, 2, 4 |  |
| labs_calcium_gc_min | Laboratory | Min total calcium value | SCM EMR | numeric | N | 1, 2, 4 |  |
| labs_calcium_gc_sd | Laboratory | SD of total calcium values | SCM EMR | numeric | N | 1, 2, 4 |  |
| labs_cholesterol_max | Laboratory | Max cholesterol value | SCM EMR | numeric | N | 1, 2, 4 |  |
| labs_cholesterol_mean | Laboratory | Mean cholesterol value | SCM EMR | numeric | N | 1, 2, 4 |  |
| labs_cholesterol_min | Laboratory | Min cholesterol value | SCM EMR | numeric | N | 1, 2, 4 |  |
| labs_cholesterol_sd | Laboratory | SD of cholesterol values | SCM EMR | numeric | N | 1, 2, 4 |  |
| labs_ck_max | Laboratory | Max CK value | SCM EMR | numeric | N | 1, 2, 4 |  |
| labs_ck_mean | Laboratory | Mean CK value | SCM EMR | numeric | N | 1, 2, 4 |  |
| labs_ck_min | Laboratory | Min CK value | SCM EMR | numeric | N | 1, 2, 4 |  |
| labs_ck_sd | Laboratory | SD of CK values | SCM EMR | numeric | N | 1, 2, 4 |  |
| labs_cl_mean | Laboratory | Mean chloride value | SCM EMR | numeric | N | 1, 2, 4 |  |
| labs_cl_sd | Laboratory | SD of chloride values | SCM EMR | numeric | N | 1, 2, 4 |  |
| labs_co2_mean | Laboratory | Mean CO2 content value | SCM EMR | numeric | N | 1, 2, 4 |  |
| labs_co2_sd | Laboratory | SD of CO2 content values | SCM EMR | numeric | N | 1, 2, 4 |  |
| labs_creatinine_mean | Laboratory | Mean creatinine value | SCM EMR | numeric | N | 1, 2, 4 |  |
| labs_crp_max | Laboratory | Max C-Reactive Protein value | SCM EMR | numeric | N | 1, 2, 4 |  |
| labs_crp_mean | Laboratory | Mean C-Reactive Protein value | SCM EMR | numeric | N | 1, 2, 4 |  |
| labs_crp_min | Laboratory | Min C-Reactive Protein value | SCM EMR | numeric | N | 1, 2, 4 |  |
| labs_crp_sd | Laboratory | SD of C-Reactive Protein values | SCM EMR | numeric | N | 1, 2, 4 |  |
| labs_ddimer_max | Laboratory | Max D-Dimer value | SCM EMR | numeric | N | 1, 2, 4 |  |
| labs_ddimer_mean | Laboratory | Mean D-Dimer value | SCM EMR | numeric | N | 1, 2, 4 |  |
| labs_ddimer_min | Laboratory | Min D-Dimer value | SCM EMR | numeric | N | 1, 2, 4 |  |
| labs_ddimer_sd | Laboratory | SD of D-Dimer values | SCM EMR | numeric | N | 1, 2, 4 |  |
| labs_ferritin_max | Laboratory | Max ferritin value | SCM EMR | numeric | N | 1, 2, 4 |  |
| labs_ferritin_mean | Laboratory | Mean ferritin value | SCM EMR | numeric | N | 1, 2, 4 |  |
| labs_ferritin_min | Laboratory | Min ferritin value | SCM EMR | numeric | N | 1, 2, 4 |  |
| labs_ferritin_sd | Laboratory | SD of ferritin values | SCM EMR | numeric | N | 1, 2, 4 |  |
| labs_fibr_max | Laboratory | Max fibrinogen value | SCM EMR | numeric | N | 1, 2, 4 |  |
| labs_fibr_mean | Laboratory | Mean fibrinogen value | SCM EMR | numeric | N | 1, 2, 4 |  |
| labs_fibr_min | Laboratory | Min fibrinogen value | SCM EMR | numeric | N | 1, 2, 4 |  |
| labs_fibr_sd | Laboratory | SD of fibrinogen values | SCM EMR | numeric | N | 1, 2, 4 |  |
| labs_gfr_mean | Laboratory | Mean eGFR value | SCM EMR | numeric | N | 1, 2, 4 |  |
| labs_ggt_max | Laboratory | Max GGT value | SCM EMR | numeric | N | 1, 2, 4 |  |
| labs_ggt_mean | Laboratory | Mean GGT value | SCM EMR | numeric | N | 1, 2, 4 |  |
| labs_ggt_min | Laboratory | Min GGT value | SCM EMR | numeric | N | 1, 2, 4 |  |
| labs_ggt_sd | Laboratory | SD of GGT values | SCM EMR | numeric | N | 1, 2, 4 |  |
| labs_glucose_mean | Laboratory | Mean glucose value | SCM EMR | numeric | N | 1, 2, 4 |  |
| labs_glucose_sd | Laboratory | SD of glucose values | SCM EMR | numeric | N | 1, 2, 4 |  |
| labs_hemato_mean | Laboratory | Mean hematocrit value | SCM EMR | numeric | N | 1, 2, 4 |  |
| labs_hemato_sd | Laboratory | SD of hematocrit values | SCM EMR | numeric | N | 1, 2, 4 |  |
| labs_hemoglobin_mean | Laboratory | Mean hemoglobin value | SCM EMR | numeric | N | 1, 2, 4 |  |
| labs_inr_mean | Laboratory | Mean INR value | SCM EMR | numeric | N | 1, 2, 4 |  |
| labs_k_mean | Laboratory | Mean potassium value | SCM EMR | numeric | N | 1, 2, 4 |  |
| labs_ld_max | Laboratory | Max LD value | SCM EMR | numeric | N | 1, 2, 4 |  |
| labs_ld_mean | Laboratory | Mean LD value | SCM EMR | numeric | N | 1, 2, 4 |  |
| labs_ld_min | Laboratory | Min LD value | SCM EMR | numeric | N | 1, 2, 4 |  |
| labs_ld_sd | Laboratory | SD of LD values | SCM EMR | numeric | N | 1, 2, 4 |  |
| labs_ldl_max | Laboratory | Max LDL value | SCM EMR | numeric | N | 1, 2, 4 |  |
| labs_ldl_mean | Laboratory | Mean LDL value | SCM EMR | numeric | N | 1, 2, 4 |  |
| labs_ldl_min | Laboratory | Min LDL value | SCM EMR | numeric | N | 1, 2, 4 |  |
| labs_ldl_sd | Laboratory | SD of LDL values | SCM EMR | numeric | N | 1, 2, 4 |  |
| labs_leukocyte_max | Laboratory | Max leukocyte value | SCM EMR | numeric | N | 1, 2, 4 |  |
| labs_leukocyte_mean | Laboratory | Mean leukocyte value | SCM EMR | numeric | N | 1, 2, 4 |  |
| labs_leukocyte_min | Laboratory | Min leukocyte value | SCM EMR | numeric | N | 1, 2, 4 |  |
| labs_leukocyte_sd | Laboratory | SD of leukocyte values | SCM EMR | numeric | N | 1, 2, 4 |  |
| labs_lymphs_max | Laboratory | Max lymphocytes value | SCM EMR | numeric | N | 1, 2, 4 |  |
| labs_lymphs_mean | Laboratory | Mean lymphocytes value | SCM EMR | numeric | N | 1, 2, 4 |  |
| labs_lymphs_min | Laboratory | Min lymphocytes value | SCM EMR | numeric | N | 1, 2, 4 |  |
| labs_lymphs_sd | Laboratory | SD of lymphocytes values | SCM EMR | numeric | N | 1, 2, 4 |  |
| labs_mchc_mean | Laboratory | Mean MCHC value | SCM EMR | numeric | N | 1, 2, 4 |  |
| labs_mcv_mean | Laboratory | Mean MCV value | SCM EMR | numeric | N | 1, 2, 4 |  |
| labs_na_mean | Laboratory | Mean sodium value | SCM EMR | numeric | N | 1, 2, 4 |  |
| labs_neutro_mean | Laboratory | Mean neutrophils value | SCM EMR | numeric | N | 1, 2, 4 |  |
| labs_pco2_art_max | Laboratory | Max PCO2 arterial value | SCM EMR | numeric | N | 1, 2, 4 |  |
| labs_pco2_art_mean | Laboratory | Mean PCO2 arterial value | SCM EMR | numeric | N | 1, 2, 4 |  |
| labs_pco2_art_min | Laboratory | Min PCO2 arterial value | SCM EMR | numeric | N | 1, 2, 4 |  |
| labs_PF_lab_max | Laboratory | Max P/F ratio in lab data | SCM EMR | numeric | N | 1, 2, 4 |  |
| labs_PF_lab_mean | Laboratory | Mean P/F ratio in lab data | SCM EMR | numeric | N | 1, 2, 4 |  |
| labs_PF_lab_min | Laboratory | Min P/F ratio in lab data | SCM EMR | numeric | N | 1, 2, 4 |  |
| labs_pltct_mean | Laboratory | Mean platelet counts | SCM EMR | numeric | N | 1, 2, 4 |  |
| labs_protein_total_max | Laboratory | Max protein total value | SCM EMR | numeric | N | 1, 2, 4 |  |
| labs_protein_total_mean | Laboratory | Mean protein total value | SCM EMR | numeric | N | 1, 2, 4 |  |
| labs_protein_total_min | Laboratory | Min protein total value | SCM EMR | numeric | N | 1, 2, 4 |  |
| labs_protein_total_sd | Laboratory | SD of protein total values | SCM EMR | numeric | N | 1, 2, 4 |  |
| labs_ptt_mean | Laboratory | Mean PTT value | SCM EMR | numeric | N | 1, 2, 4 |  |
| labs_rdw_mean | Laboratory | Mean RDW value | SCM EMR | numeric | N | 1, 2, 4 |  |
| labs_triglycerides_max | Laboratory | Max triglycerides value | SCM EMR | numeric | N | 1, 2, 4 |  |
| labs_triglycerides_mean | Laboratory | Mean triglycerides value | SCM EMR | numeric | N | 1, 2, 4 |  |
| labs_triglycerides_min | Laboratory | Min triglycerides value | SCM EMR | numeric | N | 1, 2, 4 |  |
| labs_triglycerides_sd | Laboratory | SD of triglycerides values | SCM EMR | numeric | N | 1, 2, 4 |  |
| labs_troponin_mean | Laboratory | Mean troponin value | SCM EMR | numeric | N | 1, 2, 4 |  |
| labs_troponin_sd | Laboratory | SD of troponin values | SCM EMR | numeric | N | 1, 2, 4 |  |
| labs_urea_mean | Laboratory | Mean Urea value | SCM EMR | numeric | N | 1, 2, 4 |  |
| labs_WBC_mean | Laboratory | Mean WBC value | SCM EMR | numeric | N | 1, 2, 4 |  |
| labs_WBC_sd | Laboratory | SD of WBC values | SCM EMR | numeric | N | 1, 2, 4 |  |
| med_aminoglycosides | Medications | Taken aminoglycoside drugs | SCM EMR | 0/1 | Y |  | 0 |
| med_anthelmintics | Medications | Taken anthelmintic drugs | SCM EMR | 0/1 | Y |  | 0 |
| med_antifungals | Medications | Taken antifungal drugs | SCM EMR | 0/1 | Y |  | 0 |
| med_antimalarial | Medications | Taken antimalarial drugs | SCM EMR | 0/1 | Y |  | 0 |
| med_antituberculosis | Medications | Taken antituberculosis drugs | SCM EMR | 0/1 | Y |  | 0 |
| med_antiviral | Medications | Taken antiviral drugs | SCM EMR | 0/1 | Y |  | 0 |
| med_carbapenems | Medications | Taken carbapenem drugs | SCM EMR | 0/1 | Y |  | 0 |
| med_cephalosporins | Medications | Taken cephalosporin drugs | SCM EMR | 0/1 | Y |  | 0 |
| med_glycopeptide | Medications | Taken glycopeptide drugs | SCM EMR | 0/1 | Y |  | 0 |
| med_glycylcyclines | Medications | Taken glycylcycline drugs | SCM EMR | 0/1 | Y |  | 0 |
| med_leprostatics | Medications | Taken leprostatic drugs | SCM EMR | 0/1 | Y |  | 0 |
| med_lincomycin | Medications | Taken lincomycin drugs | SCM EMR | 0/1 | Y |  | 0 |
| med_macrolide | Medications | Taken macrolide drugs | SCM EMR | 0/1 | Y |  | 0 |
| med_miscellaneous | Medications | Taken miscellaneous drugs | SCM EMR | 0/1 | Y |  | 0 |
| med_oxazolidinone | Medications | Taken oxazolidinone drugs | SCM EMR | 0/1 | Y |  | 0 |
| med_penicillins | Medications | Taken penicillin drugs | SCM EMR | 0/1 | Y |  | 0 |
| med_quinolones | Medications | Taken quinolone drugs | SCM EMR | 0/1 | Y |  | 0 |
| med_sulfonamides | Medications | Taken sulfonamide drugs | SCM EMR | 0/1 | Y |  | 0 |
| med_tetracyclines | Medications | Taken tetracycline drugs | SCM EMR | 0/1 | Y |  | 0 |
| med_urinary | Medications | Taken urinary system-related drugs | SCM EMR | 0/1 | Y |  | 0 |
| vasop_ind | Medications | Taken vasopressor drugs | SCM EMR | 0/1 | Y |  | 0 |
| pf_ratio_min | Physiology | Min P/F ratio in ICU data | eCritical | numeric | N | 1, 4 |  |
| abx_2day | Treatment | Taken antimicrobials for 2 or more consecutive days | SCM EMR | 0/1 | Y |  | 0 |
| abx_ind | Treatment | Taken antimicrobials (anytime) | SCM EMR | 0/1 | Y |  | 0 |
| imv | Treatment | Received invasive mechanical ventilation | eCritical | 0/1 | Y |  | 0 |
| anybloodprod | Treatment | Received any blood products | SCM EMR | 0/1 | N | 1, 4 |  |
| bloodprod_1 | Treatment | RBC transfusion | SCM EMR | 0/1 | N | 1, 4 |  |
| bloodprod_2 | Treatment | Platelet transfusion | SCM EMR | 0/1 | N | 1, 4 |  |
| bloodprod_3 | Treatment | Plasma transfusion | SCM EMR | 0/1 | N | 1, 4 |  |
| bloodprod_4 | Treatment | Cryoprecipitate transfusion | SCM EMR | 0/1 | N | 1, 4 |  |
| bloodprod_5 | Treatment | IVIG infusion | SCM EMR | 0/1 | N | 1, 4 |  |
| bloodprod_6 | Treatment | SCIG infusion | SCM EMR | 0/1 | N | 1, 4 |  |
| bloodprod_7 | Treatment | Albumin infusion | SCM EMR | 0/1 | N | 1, 4 |  |
| bloodprod_8 | Treatment | Factor product infusion | SCM EMR | 0/1 | N | 1, 4 |  |
| bloodprod_9 | Treatment | Other blood products | SCM EMR | 0/1 | N | 1, 4 |  |
| dialysis | Treatment | Received dialysis | DAD | 0/1 | N | 2, 4 |  |
| imv_cci_endDT | Treatment | IMV start date and time | DAD | date and time | N | 3 |  |
| imv_cci_startDT | Treatment | IMV end date and time | DAD | date and time | N | 3 |  |
| labs_FiO2_max | Treatment | Max FiO2 value | SCM EMR | numeric | N | 1, 2, 4 |  |
| labs_FiO2_mean | Treatment | Mean FiO2 value | SCM EMR | numeric | N | 1, 2, 4 |  |
| labs_FiO2_min | Treatment | Min FiO2 value | SCM EMR | numeric | N | 1, 2, 4 |  |
| multibloodprod | Treatment | Received multiple blood products | SCM EMR | 0/1 | N | 1, 4 |  |
| nbloodprod_1 | Treatment | Number of RBC transfusions | SCM EMR | numeric | N | 1, 4 |  |
| nbloodprod_2 | Treatment | Number of platelet transfusions | SCM EMR | numeric | N | 1, 4 |  |
| nbloodprod_3 | Treatment | Number of plasma transfusions | SCM EMR | numeric | N | 1, 4 |  |
| nbloodprod_4 | Treatment | Number of Cryoprecipitate transfusions | SCM EMR | numeric | N | 1, 4 |  |
| nbloodprod_5 | Treatment | Number of IVIG infusions | SCM EMR | numeric | N | 1, 4 |  |
| nbloodprod_6 | Treatment | Number of SCIG infusions | SCM EMR | numeric | N | 1, 4 |  |
| nbloodprod_7 | Treatment | Number of Albumin infusions | SCM EMR | numeric | N | 1, 4 |  |
| nbloodprod_8 | Treatment | Number of factor product infusions | SCM EMR | numeric | N | 1, 4 |  |
| nbloodprod_9 | Treatment | Number of other blood products | SCM EMR | numeric | N | 1, 4 |  |

DAD: Discharge Abstract Database; NACRS: National Ambulatory Care Reporting System; SCM EMR: Sunrise Clinical Manager (SCM) Electronic Medical Record (EMR) database; SCU: Special care unit; ED: Emergency department; ICU: Intensive care unit; SD: Standard deviation, calculated by two or more measures during the entire hospitalization per patient. For lab tests with only one result value, the SD was set to 0; IMV: Invasive mechanical ventilation; RBC: Red blood cell; IVIG: Intravenous immunoglobin; SCIG: Subcutaneous immunoglobin; ALP: Alkaline phosphatase; ALT: Alanine transaminase; eGFR: Estimated glomerular filtration rate; INR: International normalized ratio; MCHC: Mean corpuscular hemoglobin concentration; MCV: Mean corpuscular volume; PTT: Partial thromboplastin time; RDW: Red cell distribution width; WBC: White blood count; ECG: Electrocardiogram; CK: Creatine kinase; CL: Chloride; GGT: Gamma-glutamyl transferase.

The presence of the 31 chronic comorbid conditions was determined using the Elixhauser Comorbidity Index, defined from ICD-10-CA codes as per the methodology outlined by Quan *et al.*^22^. The ICD-10 chapters were defined by the most responsible diagnosis^25^ according to the user guide of ICD-10-CA^26^.

Reason for exclusion: 1, <1% prevalence (e.g., AIDS/HIV); 2, High collinearity with others; 3, Date/time variables since their derived variables were included; 4, Deemed irrelevant or duplicative by clinical experts in our investigator group; 5, Direct outcome variables.

The percentages of missing values for the variables included in the analysis were reported.

# References

1. Canadian Institute for Health Information. Discharge Abstract Database (DAD) metadata. 2020; <https://www.cihi.ca/en/discharge-abstract-database-metadata-dad>. Accessed November 14, 2022.

2. Canadian Institute for Health Information. National Ambulatory Care Reporting System (NACRS) metadata. <https://www.cihi.ca/en/national-ambulatory-care-reporting-system-nacrs-metadata>. Accessed November 14, 2023.

3. Alberta Health Services. Sunrise Clinical Manager Training for Physicians. <https://www.albertahealthservices.ca/webapps/elearning/SCM/PhysicianLaunch/index.html?jmptopg=a001_scm_training_for_physicians.html>. Accessed November 26, 2023.

4. Bowker SL, Stelfox HT, Bagshaw SM, Critical Care Strategic Clinical N. Critical Care Strategic Clinical Network: Information infrastructure ensures a learning health system. *CMAJ.* 2019;191(Suppl):S22-S23.

5. Selim SZ, Ismail MA. K-means-type algorithms: a generalized convergence theorem and characterization of local optimality. *IEEE Trans Pattern Anal Mach Intell.* 1984;6(1):81-87.

6. Reynolds D. Gaussian Mixture Models. In: Li SZ, Jain A, eds. *Encyclopedia of Biometrics.* Boston, MA: Springer US; 2009:659-663.

7. Kaufman L, Rousseeuw P. Clustering Large Applications (Program CLARA). In: Kaufman L, Rousseeuw P, eds. *Finding Groups in Data.*1990.

8. Hicks SC, Liu R, Ni Y, Purdom E, Risso D. mbkmeans: Fast clustering for single cell data using mini-batch k-means. *PLoS Comput Biol.* 2021;17(1):e1008625.

9. Jing L, Ng MK, Huang JZ. An Entropy Weighting k-Means Algorithm for Subspace Clustering of High-Dimensional Sparse Data. *IEEE Transactions on Knowledge and Data Engineering.* 2007;19(8):1026-1041.

10. Brodinová Š, Filzmoser P, Ortner T, Breiteneder C, Rohm M. Robust and sparse k-means clustering for high-dimensional data. *Advances in Data Analysis and Classification.* 2019;13(4):905-932.

11. Kaski S. Self-Organizing Maps. In: Sammut C, Webb GI, eds. *Encyclopedia of Machine Learning.* Boston, MA: Springer US; 2010:886-888.

12. Shao W, Luo X, Zhang Z, et al. Application of unsupervised deep learning algorithms for identification of specific clusters of chronic cough patients from EMR data. *BMC Bioinformatics.* 2022;23(Suppl 3):140.

13. Jolley RJ, Quan H, Jette N, et al. Validation and optimisation of an ICD-10-coded case definition for sepsis using administrative health data. *BMJ Open.* 2015;5(12):e009487.

14. Centers for Disease Control and Prevention. Hospital Toolkit for Adult Sepsis Surveillance. 2018; <https://www.cdc.gov/sepsis/pdfs/Sepsis-Surveillance-Toolkit-Mar-2018_508.pdf>. Accessed December 02, 2023.

15. Vellinga NAR, Boerma EC, Koopmans M, et al. Mildly elevated lactate levels are associated with microcirculatory flow abnormalities and increased mortality: a microSOAP post hoc analysis. *Crit Care.* 2017;21(1):255.

16. Kidney Disease: Improving Global Outcomes (KDIGO) Acute Kidney Injury Work Group. KDIGO Clinical Practice Guideline for Acute Kidney Injury. *Kidney International Supplements.* 2012;2(1):1-138.

17. Moreno R, Rhodes A, Piquilloud L, et al. The Sequential Organ Failure Assessment (SOFA) Score: has the time come for an update? *Critical Care.* 2023;27(1):15.

18. Oliveira GB, Crespo EM, Becker RC, et al. Incidence and prognostic significance of thrombocytopenia in patients treated with prolonged heparin therapy. *Arch Intern Med.* 2008;168(1):94-102.

19. Cuker A, Cines DB. How I treat heparin-induced thrombocytopenia. *Blood.* 2012;119(10):2209-2218.

20. Vincent JL, de Mendonca A, Cantraine F, et al. Use of the SOFA score to assess the incidence of organ dysfunction/failure in intensive care units: results of a multicenter, prospective study. Working group on "sepsis-related problems" of the European Society of Intensive Care Medicine. *Crit Care Med.* 1998;26(11):1793-1800.

21. Vincent JL, Moreno R, Takala J, et al. The SOFA (Sepsis-related Organ Failure Assessment) score to describe organ dysfunction/failure. On behalf of the Working Group on Sepsis-Related Problems of the European Society of Intensive Care Medicine. *Intensive Care Med.* 1996;22(7):707-710.

22. Quan H, Sundararajan V, Halfon P, et al. Coding algorithms for defining comorbidities in ICD-9-CM and ICD-10 administrative data. *Med Care.* 2005;43(11):1130-1139.

23. Eisenman RL. A profit-sharing interpretation of Shapley value for N-person games. *Behav Sci.* 1967;12(5):396-398.

24. Chen T, Guestrin C. XGBoost: A scalable tree boosting system. KDD '16: The 22nd ACM SIGKDD International Conference on Knowledge Discovery and Data Mining; 2016; USA.

25. Canadian Institute for Health Information. Indicator Library: Diagnosis type definitions. 2016; <https://www.cihi.ca/sites/default/files/document/diagnosis-type-definitions-en.pdf>. Accessed November 28, 2023.

26. Canadian Institute for Health Information. International Statistical Classification of Diseases and Related Health Problems, Tenth Revision, Canada. In:2018:1-761.

27. Elixhauser A, Owens P. Reasons for Being Admitted to the Hospital through the Emergency Department, 2003. Paper presented at: Healthcare Cost and Utilization Project (HCUP) Statistical Briefs2006; Rockville (MD).

28. Garland A, Olafson K, Ramsey CD, Yogendran M, Fransoo R. Epidemiology of critically ill patients in intensive care units: a population-based observational study. *Crit Care.* 2013;17(5):R212.
